# Supplementary material for: PEPhub: a database, web interface, and API for editing, sharing, and validating biological sample metadata
Source: Gigascience. 2024 Jul 11;13:giae033. doi: 10.1093/gigascience/giae033 (PMC11238423; doi:10.1093/gigascience/giae033)
Supplement: giae033_GIGA-D-23-00246_Original_Submission [file giae033_giga-d-23-00246_original_submission.pdf]

GigaScience

PEPhub: a database, web interface, and API for editing, sharing, and validating biological sample metadata

--Manuscript Draft--

**Manuscript Number:**	GIGA-D-23-00246
**Full Title:**	PEPhub: a database, web interface, and API for editing, sharing, and validating biological sample metadata
**Article Type:**	Technical Note
**Funding Information:**	NIGMS (GM128636) — Dr. Nathan C. Sheffield<br>National Human Genome Research Institute (R01-HG012558) — Dr. Nathan C. Sheffield
**Abstract:**	Background<br><br>As biological data increases, we need additional infrastructure to share it and promote interoperability. While major effort has been put into sharing data, relatively less emphasis is placed on sharing metadata. Yet, sharing metadata is also important, and in some ways has a wider scope than sharing data itself.<br><br>Results<br><br>Here, we present PEPhub, an approach to improve sharing and interoperability of biological metadata. PEPhub provides an API, natural language search, and user-friendly web-based sharing and editing of sample metadata tables. We used PEPhub to process more than 100,000 published biological research projects and index them with fast semantic natural language search. PEPhub thus provides a fast and user-friendly way to finding existing biological research data, or to share new data.<br><br>Availability<br><br>https://pephub.databio.org
**Corresponding Author:**	Nathan C. Sheffield<br><br>UNITED STATES
**Corresponding Author Secondary Information:**	
**Corresponding Author's Institution:**	
**Corresponding Author's Secondary Institution:**	
**First Author:**	Nathan J. LeRoy
**First Author Secondary Information:**	
**Order of Authors:**	Nathan J. LeRoy<br>Oleksandr Khoroshevskyi<br>Aaron O’Brien<br>Rafal Stepien<br>Alip Arslan<br>Nathan C. Sheffield
**Order of Authors Secondary Information:**	
**Additional Information:**	

Powered by Editorial Manager® and ProduXion Manager® from Aries Systems Corporation

Question	Response
Are you submitting this manuscript to a special series or article collection?	No
**Experimental design and statistics**<br><br>Full details of the experimental design and statistical methods used should be given in the Methods section, as detailed in our Minimum Standards Reporting Checklist. Information essential to interpreting the data presented should be made available in the figure legends.<br><br>Have you included all the information requested in your manuscript?	No
If not, please give reasons for any omissions below.<br><br>as follow-up to "**Experimental design and statistics**<br><br>Full details of the experimental design and statistical methods used should be given in the Methods section, as detailed in our Minimum Standards Reporting Checklist. Information essential to interpreting the data presented should be made available in the figure legends.<br><br>Have you included all the information requested in your manuscript?<br><br>"	No experimental design or statistical methods are used.
**Resources**<br><br>A description of all resources used, including antibodies, cell lines, animals and software tools, with enough information to allow them to be uniquely identified, should be included in the Methods section. Authors are strongly encouraged to cite Research Resource Identifiers (RRIDs) for antibodies, model	Yes

Powered by Editorial Manager® and ProduXion Manager® from Aries Systems Corporation

organisms and tools, where possible.<br><br>Have you included the information requested as detailed in our Minimum Standards Reporting Checklist?	
**Availability of data and materials**<br><br>All datasets and code on which the conclusions of the paper rely must be either included in your submission or deposited in publicly available repositories (where available and ethically appropriate), referencing such data using a unique identifier in the references and in the “Availability of Data and Materials” section of your manuscript.<br><br>Have you have met the above requirement as detailed in our Minimum Standards Reporting Checklist?	Yes

Powered by Editorial Manager® and ProduXion Manager® from Aries Systems Corporation

Manuscript

Click here to access/download;Manuscript;manuscript-2023-08-15.pdf

PEPhub: a database, web interface, and API for editing, sharing, and validating biological sample metadata

Nathan J. LeRoy[1, 3], Oleksandr Khoroshevskyi[1], Aaron O'Brien[1], Rafał Stepień[1], Alip Arslan[7], and Nathan C. Sheffield[1-6,✉]

[1]Center for Public Health Genomics, School of Medicine, University of Virginia, 22908, Charlottesville VA
[2]School of Data Science, University of Virginia, Charlottesville VA 22904, Charlottesville VA
[3]Department of Biomedical Engineering, School of Medicine, University of Virginia, 22904, Charlottesville VA
[4]Department of Public Health Sciences, School of Medicine, University of Virginia, 22908, Charlottesville VA
[5]Department of Biochemistry and Molecular Genetics, School of Medicine, University of Virginia, 22908, Charlottesville VA
[6]Child Health Research Center, School of Medicine, University of Virginia, 22908, Charlottesville VA
[7]Department of Computer Science, School of Engineering, University of Virginia, 22908, Charlottesville VA
✉ Correspondence: nsheffield@virginia.edu

Background: As biological data increases, we need additional infrastructure to share it and promote interoperability. While major effort has been put into sharing data, relatively less emphasis is placed on sharing metadata. Yet, sharing metadata is also important, and in some ways has a wider scope than sharing data itself. **Results:** Here, we present PEPhub, an approach to improve sharing and interoperability of biological metadata. PEPhub provides an API, natural language search, and user-friendly web-based sharing and editing of sample metadata tables. We used PEPhub to process more than 100,000 published biological research projects and index them with fast semantic natural language search. PEPhub thus provides a fast and user-friendly way to finding existing biological research data, or to share new data. **Availability:** https://pephub.databio.org

Background

The rapid pace of biological data generation has led to challenges with data sharing, storage, and integration [1–4]. Growing interest in data reusability and interoperability [5, 6] has led to new effort in improving biological data sharing and accessibility [7–9]. However, most effort has focused on biological *data*. Less emphasis has been placed on increasing the availability of biological *metadata* [10, 11].

As such, it is helpful to distinguish between *data* and *metadata*. In biology, *data* consists of experimental measurements or observations, while *metadata* describes the biological sample from which the measurements were derived. The sample metadata may include inherent, experimental, or analytical attributes about the sample. It might also describe the biology, treatments, experimental conditions, and data analysis parameters. Sharing complete biological *metadata* is important not only for integrated analysis, but also for discoverability [6]. There is a critical need for better tools and frameworks for sharing biological metadata.

To this end, tools and repositories have been developed to work with biological metadata [12–16]. However, they suffer from four main limitations: First, while metadata databases exist, they tend to focus on storage and retrieval [12]; none focus on simplifying user upload and editing of their own data. Second, metadata generally lacks a well-defined and supported structure. Previous methods tend to use a structure for a specific tool and data source [16–18], or leave the structure undefined altogether. Third, their search functionality is limited. Metadata search is generally limited to string matching or ontology searches. Finally, existing metadata services cannot easily be rebuilt and redeployed efficiently for custom use [19].

A recent advancement in biological metadata interoperability is Portable Encapsulated Projects (PEP), a framework that provides a standardized metadata structure, metadata validation, and programmatic metadata modifiers [20]. A PEP is a standardized sample table. The PEP framework provides a common infrastructure that links sample tables to analytical tools by removing the need for tedious and manual data preparation, mitigating the problem of metadata interoperability. However, there is no user-friendly web interface and API for sharing sample tables in the PEP ecosystem.

Here, we address these limitations with PEPhub: a database, web interface, search engine, and API for sharing, retrieving, and validating biological sample metadata. PEPhub provides several features that improve biological metadata interoperability, including: user- and machine-oriented interfaces, user editing and sharing, format conversion, metadata validation, natural language search, and containers for custom deployment.

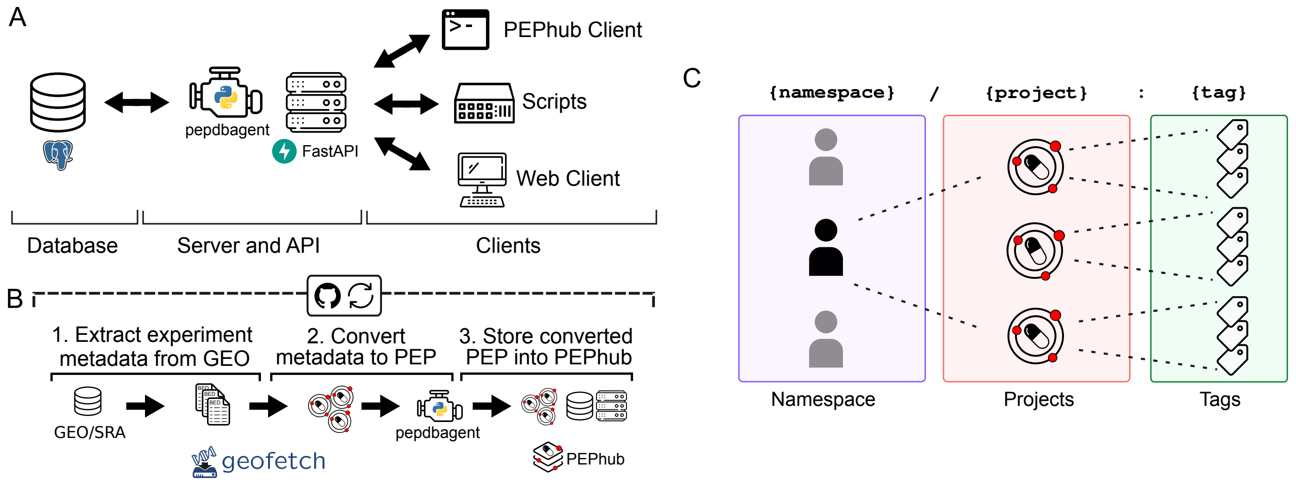

Figure 1. PEPhub high-level architecture and project identification strategy. ***A.*** *PEPhub is backed by a Postgres database (left). It interfaces with the PEPhub server through a companion package called `pepdbagent` (middle). Web requests made by the web client or command-line interface are made via HTTP (right).* ***B.*** *Workflow for automated GEO-to-PEPhub transfer using GEOfetch. We take advantage of scheduled Github Actions to automate new discovery of GEO accessions to upload.* ***C.*** *PEPhub employs a {namespace}/{project}:{tag} nomenclature for sample table identification. Namespaces contain projects, which can be further distinguished with tags.*

PEPhub advances the accessibility, discoverability, and reusability of biological sample metadata.

Results and Discussion

PEPhub instance and user interface

Public PEPhub instance

PEPhub is a web service for biological sample metadata. It is implemented as three major components that work together as modules: 1) a FastAPI web service; 2) a PostgreSQL database; 3) the PEPhubClient Python package, which provides Python and command-line interfaces to PEPhub (Figure 1A; Methods). To showcase the PEPhub software, we deployed a publicly available instance at https://pephub.databio.org. We used GEOfetch [21] to populate this public instance with over 150,000 projects (PEPs) derived from the Gene Expression Omnibus(GEO), with automated updates (Figure 1B, S1A; see Methods). PEPhub organizes projects by namespaces, corresponding to a user or organization on GitHub, thereby grouping related projects. PEPs are identified using a registry path in the form of <namespace>/<project_name>:<tag> (Figure 1C). The project name identifies a sample table. This naming convention allows convenient reference and versioning of sample metadata tables.

User- and machine-oriented interfaces

There are two primary interfaces by which users may interact with a PEPhub instance. First, the web interface provides access to PEP metadata for human browsing. It encourages data exploration and collaboration, making it easier for researchers to browse, search, submit, and edit PEPs. Second, the programmatic API allows other programs and scripts to interact with the server through HTTP requests. The API emphasizes the modularity of the PEPhub architecture and promotes interoperability with external software and services.

Format conversion

PEPhub provides programmatic interfaces to convert metadata into multiple formats. By default, PEPhub offers the ability to convert metadata into JSON, YAML, CSV, and plain-text formats (Figure 2A). To achieve this, PEPhub takes advantage of `eido`, a metadata validation engine written in Python [20]. Metadata conversion increases the interoperability of metadata, allowing it to fit into any analysis pipeline. Further, `eido` lets you write your own conversion functions, expanding the capabilities of a custom PEPhub deployment.

Natural language search

To improve biological metadata discovery, PEPhub provides a powerful natural-language search engine. The search engine is powered by pre-trained sentence transformers and a Qdrant vector database (Methods). We first use a sentence transformer to create low-dimensional vector representations of each PEP from the project-level and sample-level metadata attributes and descriptions. We store the resulting vectors inside a Qdrant vector database instance (Figure 2B). When a user provides a natural language search query, PEPhub transforms the query using the same sentence transformer in real-time, then queries the Qdrant API to retrieve the most semantically similar PEP vectors. Qdrant identifies similar PEPs by calculating nearest neighbors in vector space. PEPhub then returns the results to the client with their associated description and registry path (Figure 2C). PEPhub's search engine is superior to previous methods since the search is *semantic*. A semantic search system has three main advantages: First, the system understands the meaning of the search query and returns results with similar

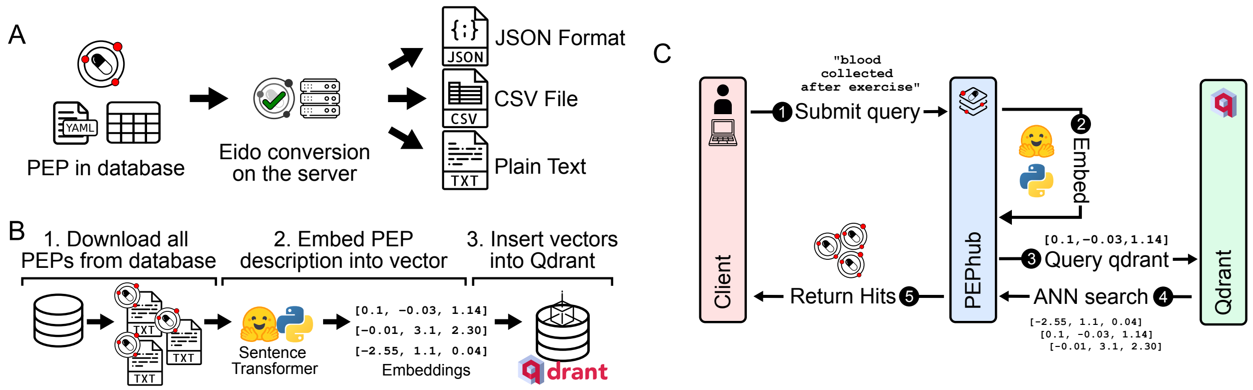

Figure 2. Metadata sharing, discovery, and accessibility features. A. *PEPhub can convert metadata into* `JSON`*,* `csv`*, and* `txt` *output.* ***B.*** *Using a pre-trained sentence transformer, we periodically compute low-dimensional embeddings of all PEPs in PEPhub by mining text descriptions from the metadata. The resulting embeddings are then stored in Qdrant: a vector similarity engine and vector database. These embeddings are then compared against user-submitted queries.* ***C.*** *Searching for a PEP in pephub using vector search happens in five steps. First, the user submits a natural language query. Second, this query is embedded in real-time on the server. Third, the resultant vector is used to query Qdrant for nearest neighbors. Fourth, Qdrant responds with the most similar vectors it has stored. Finally, the hits are returned to the client submitting the query.*

meaning whether or not they include the terms of the original query. Second, it's robust. The search system is tolerant of misspellings and is not limited to any ontology or taxonomy. Finally, the search is fast. Because each PEP is indexed and represented as a vector, we can use high-speed nearest-neighbor algorithms to identify PEPs similar to a given query [22]. This method scales to millions of PEPs, and the speed is limited only by network speeds. Our search system also allows the tuning of results with limits, offsets, and relevance score cutoffs (Methods). To demonstrate the value of PEPhub's semantic search, we show how some possible search terms like "childhood blood cancer" are able to retrieve more specialized related datasets (Figure S1B).

Private and collaborative metadata upload and editing

While the natural language search and API access to standard structured metadata from GEO is valuable, one of the most important features of PEPhub is the ability for users to submit and edit their own PEPs. Users can submit and then edit their own PEPs on PEPhub through the API or through the web interface. To facilitate this, PEPhub also provides a robust authentication system. Users authenticate with PEPhub using GitHub, which provides user and organization namespaces. Users have read access to all namespaces but write access only to their namespaces. For PEPs with write access, users may mark them as *private* to restrict read access to only users with write access (Figure 3A). For example, **user1234** can edit all PEPs in the **user1234/** namespace. They may also edit all PEPs in the **org1234** namespace if they are a public member on GitHub. This ensures that only authorized users can access and modify private PEPs. By integrating authentication and authorization features, PEPhub provides a secure and controlled environment for users to interact with and manage their own PEPs while also facilitating the sharing and discovery of public PEPs to support collaborative research efforts.

Metadata validation

PEPhub also provides metadata validation. We use `eido`, a PEP validation tool based on `jsonschema`, to validate on the server [20]. There are three ways to validate metadata through PEPhub. First, you may use the web-validator UI built with the server. With the web-validator UI, you may upload your own PEP or use PEPs stored on PEPhub and validate them against either PEPhub schemas or custom schemas. Custom schemas can be uploaded or pasted directly on the UI. Second, you can take advantage of the built-in metadata builder. When editing your PEPs, PEPhub validates the PEP after each save. The interface will propagate any errors to the user. Finally, there are validation endpoints at `/eido/validate`. These allow programmatic validation of PEPs (Figure 3B).

Comparison to other tools

Currently, several biological metadata management solutions exist to help alleviate the issue of metadata accessibility and interoperability; however, these solutions suffer from one or more limitations. One example is OMeta [19]. While it shares some features with PEPhub, it is not actively maintained, it lacks a public instance, and it lacks any documentation to start a private instance. Another example is RNASeqMetaDB [23]. Like PEPhub, RNASeqMetaDB aims to solve the problem of disorganized and limited access to sample metadata that are often published alongside the data itself; however, it lacks a currently running public instance and the source code is not available to reproduce the results. Finally, Bernasconi2021 [18] and GenoSurf [12] are comprehensive genomics metadata search servers. However, they are limited in three ways: first, they don't permit users to submit their own metadata and the database

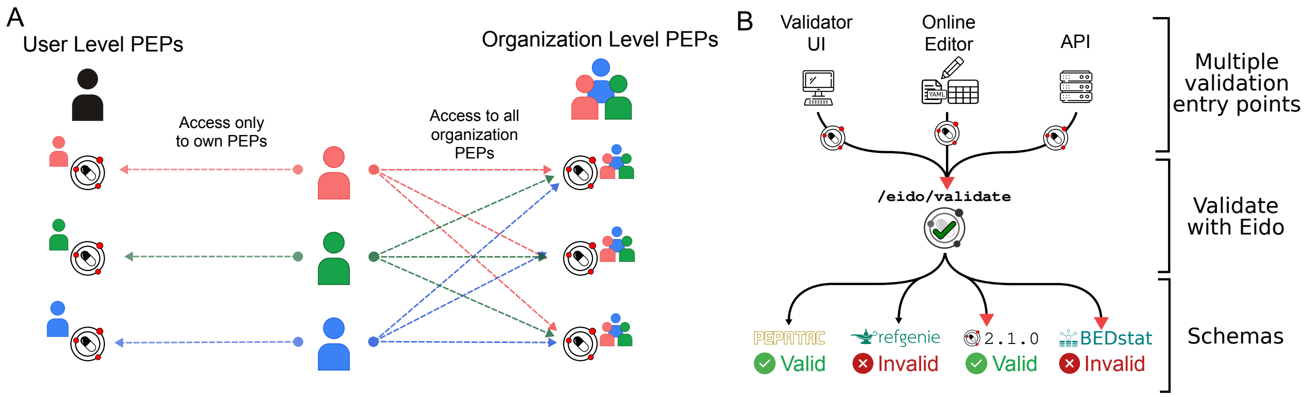

Figure 3. Metadata privacy and validation features. A. *Users have read access to all namespaces but write access only to their namespaces (left). Other users are not permitted to modify a PEP in any user namespace other than their own. PEPhub implements organizations through GitHub. Members of an organization are automatically granted write access to all PEPs that belong to that organization (right).* ***B.*** *Validation on PEPhub is made easy with the integration of* `eido`*. PEPs in PEPub can be validated using either the web-based validator UI, the metadata builder, or programmatic endpoints.*

	Open-Sourced	Self-Hosted	API	Editable Metadata	Command Line Client	ML-Based Search	Has Public Data	Actively Maintained	Public Instance
PEPhub	✓	✓	✓	✓	✓	✓	✓	✓	✓
GenoSurf	✓	✗ [a]	✓	✗	✗	✗	✓	✗	✓
EpiSurf	✓	✗ [a]	✓	✗	✗	✗	✓	✗	✓
OMeta	✓	✗ [a]	✗	✓	✗	✗	✗	✗	✗
GEO	✗	✗	✓	✗	✗	✗	✓	✓	✓
RNASeqMetaDB	✗	✗	✗	✗	✗	✗	✓	✗	✗
LabPipe	✓	✓	✓	✓	✗	✗	✗	✗	✗
METAGENOTE	✓	✗	✗	✓	✗	✗	✗	✗	✓

Figure 4. Metadata management comparison chart. *PEPhub compares favorably to alternative metadata management systems. *a: While open-source, no clear documentation exists for self hosting an instance.*

doesn't appear to be regularly updated. Second, the software is not easily deployable. Third, their search system is based solely on biological ontologies, limiting the search space and flexibility of the search system.

PEPhub has a variety of features that make it unique: First, it prioritizes allowing users to edit sample metadata stored on the server. This critical design decision positions PEPhub as not just as a place to *find* metadata, but as a place to manage and share your own. Second, it provides a full database, web API, and user interface for metadata management. Third, it provides the only metadata search engine that takes advantage of pre-trained sentence transformers for a powerful semantic search system. Fourth, it is the only tool that is open-sourced, automatically updated, actively maintained, and provides clear instructions to deploy a private instance. Together, these features make PEPhub a unique, flexible, tool that promotes the accessibility, findability, and interoperability of biological sample metadata.

Methods: Implementation and deployment details

FastAPI web service

The PEPhub server is built with FastAPI, a web framework optimized for speed and high-performance. FastAPI is specifically designed for developing APIs. We chose FastAPI for its automatic data validation capabilities, built-in API documentation, and because using Python allows us to interface with existing Python infrastructure for metadata management we developed previously [24]. The FastAPI application uses our companion package `pepdbagent` to interface with a Postgres database. The user interface is built using React.js and TypeScript, and is packaged with the server.

`pepdbagent` companion package

To manage project creation, fetching, deletion and insertion into the database, we developed a companion package called `pepdbagent`. `pepdbagent` acts as a simple wrapper around the popular Python object-relational mapper (ORM) `SQLAlchemy` to provide a convenient API for managing projects in our database. Both PEPhub itself and all maintenance scripts use `pepdbagent` to manage the PEPs stored inside the database.

PostgreSQL database

Postgres is well-suited for storing the structured and unstructured data found in PEPs because it excels at both relational and document storage. The PEPhub database is comprised of three tables. The first table, `projects` stores the PEPs metadata and PEPs configuration. The `projects` table consists of twelve columns to store data like the project timestamp, project id, and the project configuration as a JSON blob. The other two tables: `samples`, and `subsamples` store samples and subsamples that are linked with `project.id` to a specific project in the `project` table. We host the public PEPhub database instance on the Amazon Web Services Relational Database Service (AWS RDS).

PEPhubClient Python and command-line interface

To facilitate command-line interaction and third-party tools using PEPhub, we have developed PEPhubClient. PEPhubClient is a command-line interface and Python API that leverages the machine-oriented interfaces of PEPhub. Namely, the public API. PEPhubClient makes it easy to push and pull PEPs to any PEPhub instance. The command-line interface supports authentication to make authorized requests. This includes working with private PEPs, downloading PEPs, editing PEPs, and submitting PEPs. The PEPhubClient CLI is implemented in Python using the `typer` library.

Containers for custom deployment

To standardize deployment and promote interoperability, we've packaged the PEPhub server and database as docker containers. These containers are made available on dockerhub. This makes it incredibly easy to launch your own instance of PEPhub.

Populating PEPhub with biological sample metadata from GEO

To populate PEPhub, we developed a pipeline to ingest sample metadata from GEO. Our pipeline uploads PEPs from GEO in two steps. First, it identifies experiments that were added or updated in certain period of time to the Gene Expression Omnibus[7] using the GEOfetch [21] Python API. Second, GEOfetch downloads, formats and produces PEPs from GEO experiments that are later uploaded to the PEPhub database. Our database now stores more than 150,000 high-throughput sample tables from the last 10 years from Gene Expression Omnibus. We developed a pipeline that uses GitHub `schedule` actions to automate the download, formatting, and upload or re-upload of new project releases on GEO. Moreover, the pipeline includes an automatic check for successful previous uploads, ensuring that all GEO projects are consistently updated on PEPhub without the need for manual intervention.

Natural language search

To support the text mining and embedding pipeline, we developed a companion tool called `pepembed` that embeds a database of PEPs and inserts them into a Qdrant database instance. We leverage GitHub actions to run indexing tasks periodically to ensure that all PEPs stay properly indexed, even if their data change.

Authentication and authorization

PEPhub supports two authentication flows: authorization code flow and device code flow. Both take advantage of GitHub's OAuth services. In addition, both authentication flows require users to login with GitHub via a web browser, upon which a code is returned. This code is then exchanged for a JSON Web Token (JWT) via a `POST` request which can be used to make subsequently authorized requests. While very similar, both flows exist to make it as easy as possible to integrate third-party software with a PEPhub instance.

Funding

This work was supported by the National Institute of General Medical Sciences grant R35-GM128636 (NCS) and National Human Genome Research Institute grant R01-HG012558 (NCS). Funders had no role in study design, data collection, analysis, or publication.

Availability and requirements

Project name: PEPhub
Project home page: https://pephub.databio.org
Operating system: Platform independent
Programming language: Python
License: BSD-2

Conflict of interest statement

NCS is a consultant for InVitro Cell Research, LLC.

References

1. Volchenboum SL, Cox SM, Heath A, Resnick A, Cohn SL, Grossman R. Data commons to support pediatric cancer research. American Society of Clinical Oncology Educational Book. 2017;37:746–52. doi:10.14694/edbk_175029.

2. Bui AAT, Van Horn JD, Consortium NBC. Envisioning the future of 'big data' biomedicine. J Biomed Inform. 2017;69:115–7. doi:10.1016/j.jbi.2017.03.017.

3. Armit C, Tuli MA, Hunter CI. A decade of GigaScience: GigaDB and the open data movement. GigaScience. 2022;11. doi:10.1093/gigascience/giac053.

4. Xue B, Khoroshevskyi O, Gomez RA, Sheffield NC. Opportunities and challenges in sharing and reusing genomic interval data. Frontiers in Genetics. 2023;14. doi:10.3389/fgene.2023.1155809.

5. Wilkinson MD, Dumontier M, Aalbersberg IjJ, Appleton G, Axton M, Baak A, et al. The FAIR Guiding Principles for scientific data management and stewardship. Scientific Data. 2016;3:160018. doi:10.1038/sdata.2016.18.

6. Sheffield NC, Bonazzi VR, Bourne PE, Burdett T, Clark T, Grossman RL, et al. From biomedical cloud platforms to microservices: Next steps in FAIR data and analysis. Scientific Data. 2022;9:553. doi:10.1038/s41597-022-01619-5.

7. Edgar R, Domrachev M, Lash AE. Gene Expression Omnibus: NCBI gene expression and hybridization array data repository. Nucleic Acids Research. 2002;30:207–10.

8. Sloan CA, Chan ET, Davidson JM, Malladi VS, Strattan JS, Hitz BC, et al. ENCODE data at the ENCODE portal. Nucleic Acids Research. 2016;44:D726–32. doi:10.1093/nar/gkv1160.

9. Bourne PE, Bonazzi V, Dunn M, Green ED, Guyer M, Komatsoulis G, et al. The NIH Big Data to Knowledge (BD2K) initiative. Journal of the American Medical Informatics Association : JAMIA. 2015;22:1114. doi:10.1093/jamia/ocv136.

10. Leipzig J, Nüst D, Hoyt CT, Ram K, Greenberg J. The role of metadata in reproducible computational research. Patterns. 2021;2:100322. doi:10.1016/j.patter.2021.100322.

11. Sheffield N, LeRoy N, Khoroshevskyi O. Challenges to sharing sample metadata in computational genomics. Frontiers in Genetics. 2023;14.

12. Canakoglu A, Bernasconi A, Colombo A, Masseroli M, Ceri S. GenoSurf: Metadata driven semantic search system for integrated genomic datasets. Database. 2019;2019:baz132. doi:10.1093/database/baz132.

13. Serna Garcia G, Leone M, Bernasconi A, Carman MJ. GeMI: Interactive interface for transformer-based Genomic Metadata Integration. Database. 2022;2022:baac036. doi:10.1093/database/baac036.

14. Masseroli M, Pinoli P, Venco F, Kaitoua A, Jalili V, Palluzzi F, et al. GenoMetric Query Language: A novel approach to large-scale genomic data management. Bioinformatics. 2015;31:1881–8. doi:10.1093/bioinformatics/btv048.

15. Davis S, Meltzer PS. GEOquery: A bridge between the Gene Expression Omnibus (GEO) and BioConductor. Bioinformatics. 2007;23:1846–7. doi:10.1093/bioinformatics/btm254.

16. Quiñones M, Liou DT, Shyu C, Kim W, Vujkovic-Cvijin I, Belkaid Y, et al. “METAGENOTE: A simplified web platform for metadata annotation of genomic samples and streamlined submission to NCBI’s sequence read archive.” BMC Bioinformatics. 2020;21:378. doi:10.1186/s12859-020-03694-0.

17. Cappelli E, Cumbo F, Bernasconi A, Canakoglu A, Ceri S, Masseroli M, et al. OpenGDC: Unifying, Modeling, Integrating Cancer Genomic Data and Clinical Metadata. Applied Sciences. 2020;10:6367. doi:10.3390/app10186367.

18. Bernasconi A, Cilibrasi L, Al Khalaf R, Alfonsi T, Ceri S, Pinoli P, et al. EpiSurf: Metadata-driven search server for analyzing amino acid changes within epitopes of SARS-CoV-2 and other viral species. Database. 2021;2021:baab059. doi:10.1093/database/baab059.

19. Singh I, Kuscuoglu M, Harkins DM, Sutton G, Fouts DE, Nelson KE. OMeta: An ontology-based, data-driven metadata tracking system. BMC bioinformatics. 2019;20:8. doi:10.1186/s12859-018-2580-9.

20. Sheffield NC, Stolarczyk M, Reuter VP, Rendeiro AF. Linking big biomedical datasets to modular analysis with Portable Encapsulated Projects. 2021;2020.10.08.331322. doi:10.1101/2020.10.08.331322.

21. Khoroshevskyi O, LeRoy N, Reuter VP, Sheffield NC. GEOfetch: A command-line tool for downloading data and standardized metadata from GEO and SRA. Bioinformatics. 2023;btad069. doi:10.1093/bioinformatics/btad069.

22. Malkov YA, Yashunin DA. Efficient and robust approximate nearest neighbor search using Hierarchical Navigable Small World graphs. 2018. doi:10.48550/arXiv.1603.09320.

23. Guo Z, Tzvetkova B, Bassik JM, Bodziak T, Wojnar BM, Qiao W, et al. RNASeqMetaDB: A database and web server for navigating metadata of publicly available mouse RNA-Seq datasets. Bioinformatics (Oxford, England). 2015;31:4038–40. doi:10.1093/bioinformatics/btv503.

24. Sheffield NC, Stolarczyk M, Reuter VP, Rendeiro AF. Linking big biomedical datasets to modular analysis with Portable Encapsulated Projects. 2021;2020.10.08.331322. doi:10.1101/2020.10.08.331322.

Supplemental figures

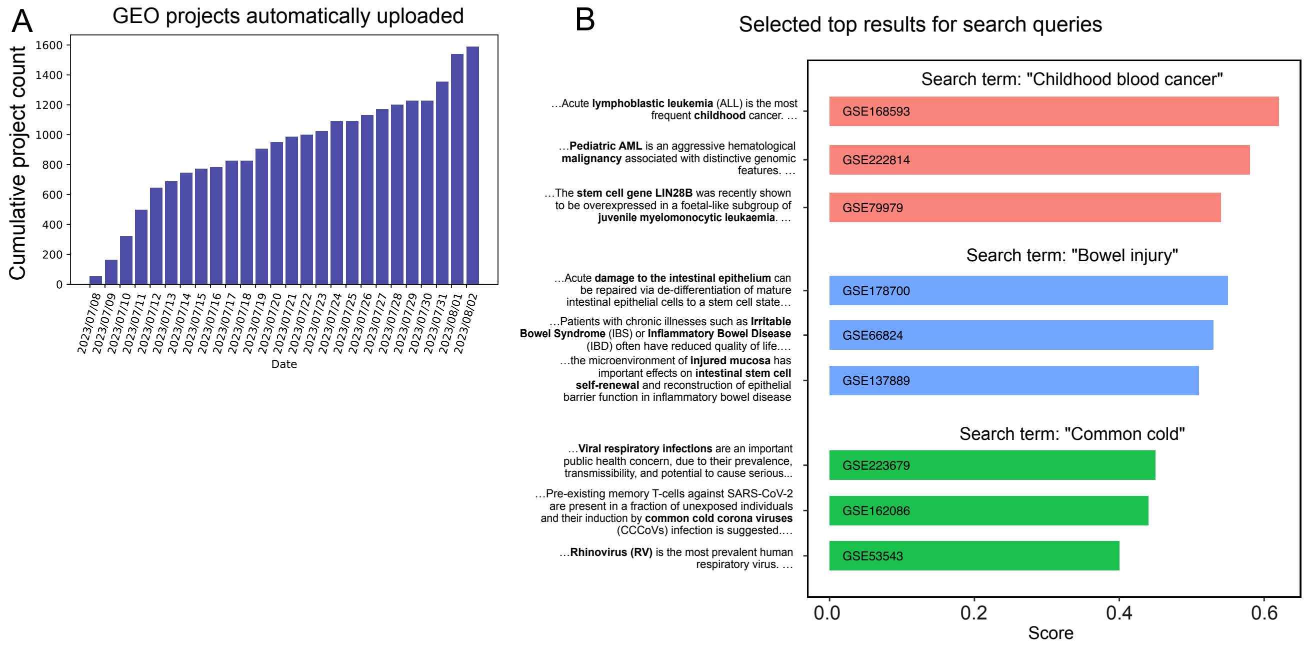

Supplementary Figure S1. Analysis of PEP metadata and search. *A) Barchart showing the cumulative number of new biological sample tables added to PEPhub from GEO, automatically. PEPhub automatically indexed more than 1000 new projects during this 3-week demo span. B) Illustrative search result scores selected from the top 10 responses returned by the PEPhub semantic search engine for biological search terms shown*
