# Supplementary material for: PEPhub: a database, web interface, and API for editing, sharing, and validating biological sample metadata
Source: Gigascience. 2024 Jul 11;13:giae033. doi: 10.1093/gigascience/giae033 (PMC11238423; doi:10.1093/gigascience/giae033)
Supplement: giae033_GIGA-D-23-00246_Revision_1 [file giae033_giga-d-23-00246_revision_1.pdf]

GigaScience

PEPhub: a database, web interface, and API for editing, sharing, and validating biological sample metadata

--Manuscript Draft--

**Manuscript Number:**	GIGA-D-23-00246R1
**Full Title:**	PEPhub: a database, web interface, and API for editing, sharing, and validating biological sample metadata
**Article Type:**	Technical Note
**Funding Information:**	NIGMS (GM128636) — Dr. Nathan C. Sheffield<br>National Human Genome Research Institute (R01-HG012558) — Dr. Nathan C. Sheffield
**Abstract:**	Background<br><br>As biological data increases, we need additional infrastructure to share it and promote interoperability. While major effort has been put into sharing data, relatively less emphasis is placed on sharing metadata. Yet, sharing metadata is also important, and in some ways has a wider scope than sharing data itself.<br><br>Results<br><br>Here, we present PEPhub, an approach to improve sharing and interoperability of biological metadata. PEPhub provides an API, natural language search, and user-friendly web-based sharing and editing of sample metadata tables. We used PEPhub to process more than 100,000 published biological research projects and index them with fast semantic natural language search. PEPhub thus provides a fast and user-friendly way to finding existing biological research data, or to share new data.<br><br>Availability<br><br>https://pephub.databio.org
**Corresponding Author:**	Nathan C. Sheffield<br><br>UNITED STATES
**Corresponding Author Secondary Information:**	
**Corresponding Author's Institution:**	
**Corresponding Author's Secondary Institution:**	
**First Author:**	Nathan J. LeRoy
**First Author Secondary Information:**	
**Order of Authors:**	Nathan J. LeRoy<br>Oleksandr Khoroshevskyi<br>Aaron O’Brien<br>Rafal Stepien<br>Alip Arslan<br>Nathan C. Sheffield
**Order of Authors Secondary Information:**	
**Response to Reviewers:**	Response to review uploaded as PDF file.

Powered by Editorial Manager® and ProduXion Manager® from Aries Systems Corporation

Additional Information:	
**Question**	**Response**
Are you submitting this manuscript to a special series or article collection?	No
**Experimental design and statistics**<br><br>Full details of the experimental design and statistical methods used should be given in the Methods section, as detailed in our Minimum Standards Reporting Checklist. Information essential to interpreting the data presented should be made available in the figure legends.<br><br>Have you included all the information requested in your manuscript?	No
If not, please give reasons for any omissions below.<br><br>as follow-up to "**Experimental design and statistics**<br><br>Full details of the experimental design and statistical methods used should be given in the Methods section, as detailed in our Minimum Standards Reporting Checklist. Information essential to interpreting the data presented should be made available in the figure legends.<br><br>Have you included all the information requested in your manuscript?<br><br>"	No experimental design or statistical methods are used.
**Resources**<br><br>A description of all resources used, including antibodies, cell lines, animals and software tools, with enough information to allow them to be uniquely identified, should be included in the Methods section. Authors are strongly encouraged to cite Research Resource	Yes

Powered by Editorial Manager® and ProduXion Manager® from Aries Systems Corporation

Identifiers (RRIDs) for antibodies, model organisms and tools, where possible.<br><br>Have you included the information requested as detailed in our Minimum Standards Reporting Checklist?	
**Availability of data and materials**<br><br>All datasets and code on which the conclusions of the paper rely must be either included in your submission or deposited in publicly available repositories (where available and ethically appropriate), referencing such data using a unique identifier in the references and in the “Availability of Data and Materials” section of your manuscript.<br><br>Have you have met the above requirement as detailed in our Minimum Standards Reporting Checklist?	Yes

Manuscript

Click here to access/download;Manuscript;manuscript-2024-02-07.pdf

RESEARCH ARTICLE

PEPhub: a database, web interface, and API for editing, sharing, and validating biological sample metadata

Nathan J. LeRoy[1, 3], Oleksandr Khoroshevskyi[1], Aaron O'Brien[1], Rafał Stepień[1], Alip Arslan[7], and Nathan C. Sheffield[1-6,✉]

[1]Center for Public Health Genomics, School of Medicine, University of Virginia, 22908, Charlottesville VA
[2]School of Data Science, University of Virginia, Charlottesville VA 22904, Charlottesville VA
[3]Department of Biomedical Engineering, School of Medicine, University of Virginia, 22904, Charlottesville VA
[4]Department of Public Health Sciences, School of Medicine, University of Virginia, 22908, Charlottesville VA
[5]Department of Biochemistry and Molecular Genetics, School of Medicine, University of Virginia, 22908, Charlottesville VA
[6]Child Health Research Center, School of Medicine, University of Virginia, 22908, Charlottesville VA
[7]Department of Computer Science, School of Engineering, University of Virginia, 22908, Charlottesville VA
✉ Correspondence: nsheffield@virginia.edu

Background: As biological data increases, we need additional infrastructure to share it and promote interoperability. While major effort has been put into sharing data, relatively less emphasis is placed on sharing metadata. Yet, sharing metadata is also important, and in some ways has a wider scope than sharing data itself. **Results:** Here, we present PEPhub, an approach to improve sharing and interoperability of biological metadata. PEPhub provides an API, natural language search, and user-friendly web-based sharing and editing of sample metadata tables. We used PEPhub to process more than 100,000 published biological research projects and index them with fast semantic natural language search. PEPhub thus provides a fast and user-friendly way to finding existing biological research data, or to share new data. **Availability:** https://pephub.databio.org

Background

The rapid pace of biological data generation has led to challenges with data sharing, storage, and integration [1–4]. Growing interest in data reusability and interoperability [5, 6] has led to new effort in improving biological data sharing and accessibility [7–9]. However, most effort has focused on biological *data*. Less emphasis has been placed on increasing the availability of biological *metadata* [10, 11].

As such, it is helpful to distinguish between *data* and *metadata*. In biology, *data* consists of experimental measurements or observations, while *metadata* describes the biological sample from which the measurements were derived. The sample metadata may include inherent, experimental, or analytical attributes about the sample. It might also describe the biology, treatments, experimental conditions, and data analysis parameters. Sharing complete biological *metadata* is important not only for integrated analysis, but also for discoverability [6]. There is a critical need for better tools and frameworks for sharing biological metadata.

To this end, tools and repositories have been developed to work with biological metadata [12–16]. However, they suffer from four main limitations: First, while metadata databases exist, they tend to focus on storage and retrieval [12]; none focus on simplifying user upload and editing of their own data. Second, metadata generally lacks a well-defined and supported structure. Previous methods tend to use a structure for a specific tool and data source [16–18], or leave the structure undefined altogether. Third, their search functionality is limited. Metadata search is generally limited to string matching or ontology searches. Finally, existing metadata services cannot easily be rebuilt and redeployed efficiently for custom use [19].

A recent advancement in biological metadata interoperability is Portable Encapsulated Projects (PEP), a framework that provides a standardized metadata structure, metadata validation, and programmatic metadata modifiers [20]. A PEP is a standardized sample table. The PEP framework provides a common infrastructure that links sample tables to analytical tools by removing the need for tedious and manual data preparation, mitigating the problem of metadata interoperability. However, there is no user-friendly web interface and API for sharing sample tables in the PEP ecosystem.

Here, we address these limitations with PEPhub: a database, web interface, search engine, and API for sharing, retrieving, and validating biological sample metadata. PEPhub provides several features that improve biological metadata interoperability, including: user- and machine-oriented interfaces, user editing and sharing, format conversion, metadata validation, natural language search, and containers for custom deployment.

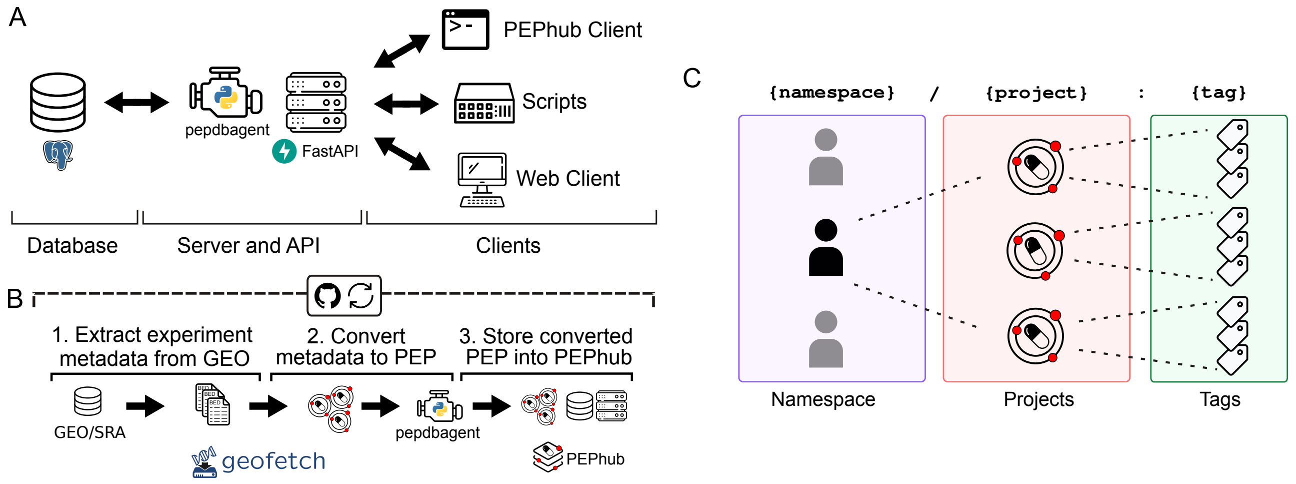

*Figure 1. **PEPhub high-level architecture and project identification strategy. A.** PEPhub is backed by a Postgres database (left). It interfaces with the PEPhub server through a companion package called `pepdbagent` (middle). Web requests made by the web client or command-line interface are made via HTTP (right). **B.** Workflow for automated GEO-to-PEPhub transfer using GEOfetch. We take advantage of scheduled Github Actions to automate new discovery of GEO accessions to upload. **C.** PEPhub employs a {namespace}/{project}:{tag} nomenclature for sample table identification. Namespaces contain projects, which can be further distinguished with tags.*

PEPhub advances the accessibility, discoverability, and reusability of biological sample metadata.

Results and Discussion

PEPhub instance and user interface

Public PEPhub instance

PEPhub is a web service for biological sample metadata. It is implemented as three major components that work together as modules: 1) a FastAPI web service; 2) a PostgreSQL database; 3) the PEPhubClient Python package, which provides Python and command-line interfaces to PEPhub (Figure 1A; Methods). To showcase the PEPhub software, we deployed a publicly available instance at https://pephub.databio.org. We used GEOfetch [21] to populate this public instance with over 150,000 projects (PEPs) derived from the Gene Expression Omnibus (GEO), with automated updates (Figure 1B, S1A; see Methods). PEPhub organizes projects by namespaces, corresponding to a user or organization on GitHub, thereby grouping related projects. PEPs are identified using a registry path in the form of <namespace>/<project_name>:<tag> (Figure 1C). The project name identifies a sample table. This naming convention allows convenient reference and versioning of sample metadata tables.

User- and machine-oriented interfaces

There are two primary interfaces by which users may interact with a PEPhub instance. First, the web interface provides access to PEP metadata for human browsing. It encourages data exploration and collaboration, making it easier for researchers to browse, search, submit, and edit PEPs. Second, the programmatic API allows other programs and scripts to interact with the server through HTTP requests. The API emphasizes the modularity of the PEPhub architecture and promotes interoperability with external software and services.

Format conversion

PEPhub provides programmatic interfaces to convert metadata into multiple formats. The standard PEP structure includes project-level attributes, a sample table, and a subsample table, which allows users to encode sample attributes with multiple values, such as sequencing reads with multiple file paths. By default, PEPhub offers the ability to convert this metadata into JSON, YAML, CSV, and plain-text formats (Figure 2A). To achieve this, PEPhub takes advantage of `eido`, a metadata validation engine written in Python [20]. Metadata conversion increases the interoperability of metadata, allowing it to fit into any analysis pipeline. Further, `eido` lets you write your own conversion functions, expanding the capabilities of a custom PEPhub deployment.

Natural language search

To improve biological metadata discovery, PEPhub provides a powerful natural-language search engine. The search engine is powered by pre-trained sentence transformers and a Qdrant vector database (Methods). We first use a sentence transformer to create low-dimensional vector representations of each PEP from the project-level and sample-level metadata attributes and descriptions. We store the resulting vectors inside a Qdrant vector database instance (Figure 2B). When a user provides a natural language search query, PEPhub transforms the query using the same sentence transformer in real-time, then queries the Qdrant API to retrieve the most semantically similar PEP vectors. Qdrant identifies similar PEPs by calculating nearest neighbors in vector space. PEPhub then returns the results to the client with their associated description

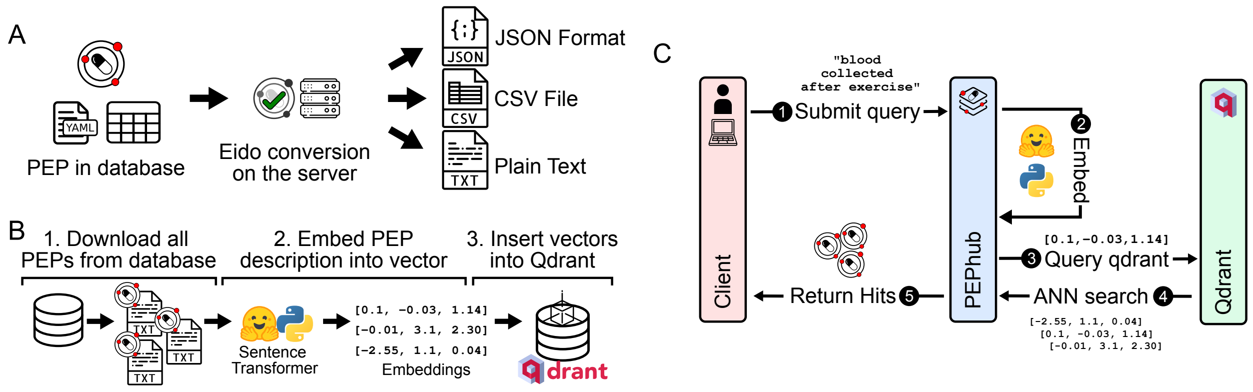

Figure 2. ***Metadata sharing, discovery, and accessibility features.*** ***A.*** *PEPhub can convert metadata into* `JSON`, `csv`, *and* `txt` *output.* ***B.*** *Using a pre-trained sentence transformer, we periodically compute low-dimensional embeddings of all PEPs in PEPhub by mining text descriptions from the metadata. The resulting embeddings are then stored in Qdrant: a vector similarity engine and vector database. These embeddings are then compared against user-submitted queries.* ***C.*** *Searching for a PEP in pephub using vector search happens in five steps. First, the user submits a natural language query. Second, this query is embedded in real-time on the server. Third, the resultant vector is used to query Qdrant for nearest neighbors. Fourth, Qdrant responds with the most similar vectors it has stored. Finally, the hits are returned to the client submitting the query.*

and registry path (Figure 2C). PEPhub's search engine is superior to previous methods since the search is *semantic*. A semantic search system has three main advantages: First, the system understands the meaning of the search query and returns results with similar meaning whether or not they include the terms of the original query. Second, it's robust. The search system is tolerant of misspellings and is not limited to any ontology or taxonomy. Finally, the search is fast. Because each PEP is indexed and represented as a vector, we can use high-speed nearest-neighbor algorithms to identify PEPs similar to a given query [22]. This method scales to millions of PEPs, and the speed is limited only by network speeds. Our search system also allows the tuning of results with limits, offsets, and relevance score cutoffs (Methods). To demonstrate the value of PEPhub's semantic search, we show how some possible search terms like "childhood blood cancer" are able to retrieve more specialized related datasets (Figure S1B, Supplementary Table 1).

Private and collaborative metadata upload and editing

While the natural language search and API access to standard structured metadata from GEO is valuable, one of the most important features of PEPhub is the ability for users to submit and edit their own PEPs. Users can submit and then edit their own PEPs on PEPhub through the API or through the web interface. To facilitate this, PEPhub also provides a robust authentication system. Users authenticate with PEPhub using GitHub, which provides user and organization namespaces. Users have read access to all namespaces but write access only to their namespaces. For PEPs with write access, users may mark them as *private* to restrict read access to only users with write access (Figure 3A). For example, **user1234** can edit all PEPs in the **user1234/** namespace. They may also edit all PEPs in the **org1234** namespace if they are a public member on GitHub. This ensures that only authorized users can access and modify private PEPs. By integrating authentication and authorization features, PEPhub provides a secure and controlled environment for users to interact with and manage their own PEPs while also facilitating the sharing and discovery of public PEPs to support collaborative research efforts.

Metadata validation

PEPhub also provides metadata validation. We use `eido`, a PEP validation tool based on `jsonschema`, to validate on the server [20]. There are three ways to validate metadata through PEPhub. First, you may use the web-validator UI built with the server. With the web-validator UI, you may upload your own PEP or use PEPs stored on PEPhub and validate them against either PEPhub schemas or custom schemas. Custom schemas can be uploaded or pasted directly on the UI. Second, you can take advantage of the built-in metadata builder. When editing your PEPs, PEPhub validates the PEP after each save. The interface will propagate any errors to the user. Finally, there are validation endpoints at `/eido/validate`. These allow programmatic validation of PEPs (Figure 3B).

Comparison to other tools

Currently, several biological metadata management solutions exist to help alleviate the issue of metadata accessibility and interoperability; however, these solutions suffer from one or more limitations. One example is OMeta [19]. While it shares some features with PEPhub, it is not actively maintained, it lacks a public instance, and it lacks any documentation to start a private instance. Another example is RNASeqMetaDB [23]. Like PEPhub, RNASeqMetaDB aims to solve the problem of disorganized and limited access to sample metadata that are often published alongside the data itself; however, it lacks a currently running public instance and the

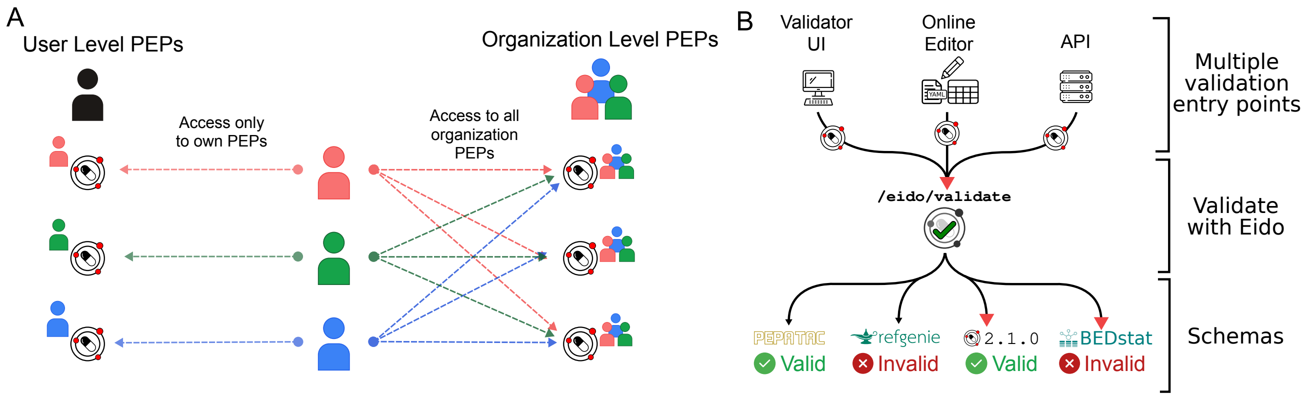

Figure 3. ***Metadata privacy and validation features.*** ***A.*** *Users have read access to all namespaces but write access only to their namespaces (left). Other users are not permitted to modify a PEP in any user namespace other than their own. PEPhub implements organizations through GitHub. Members of an organization are automatically granted write access to all PEPs that belong to that organization (right).* ***B.*** *Validation on PEPhub is made easy with the integration of* `eido`*. PEPs in PEPub can be validated using either the web-based validator UI, the metadata builder, or programmatic endpoints.*

source code is not available to reproduce the results. Finally, EpiSurf [18] and GenoSurf [12] are comprehensive genomics metadata search servers. However, they are limited in three ways: first, they don't permit users to submit their own metadata and the database doesn't appear to be regularly updated. Second, the software is not easily deployable. Third, their search system is based solely on biological ontologies, limiting the search space and flexibility of the search system.

PEPhub has several features that make it unique: First, it prioritizes allowing users to edit sample metadata stored on the server. This critical design decision positions PEPhub as not just as a place to *find* metadata, but as a place to manage and share your own. Second, it provides a full database, web API, and user interface for metadata management. Third, it provides the only metadata search engine that takes advantage of pre-trained sentence transformers for a powerful semantic search system. Fourth, it is the only tool that is open-sourced, automatically updated, actively maintained, and provides clear instructions to deploy a private instance. Together, these features make PEPhub a unique, flexible, tool that promotes the accessibility, findability, and interoperability of biological sample metadata.

PEPhub may also be compared to a Laboratory Information Management System (LIMS), a broad term with several interpretations. One difference is that LIMS tend to target management challenges related to physical sample handling in a wet lab, such as connecting to machinery, ordering reagents, and tracking physical samples through an experimental protocol. PEPhub could fulfill some of these functions, but in general, PEPhub targets a later phase of the experimental process: after data is generated, and the samples need to be analyzed and shared. PEPhub's strengths are the ability to share data easily, in a more universal form, a public API, standardization, and searching. Thus, in many cases, it may make sense for PEPhub to live alongside a LIMS.

	Open-Sourced	Self-Hosted	API	Editable Metadata	Command Line Client	ML-Based Search	Has Public Data	Actively Maintained	Public Instance
PEPhub	✓	✓	✓	✓	✓	✓	✓	✓	✓
GenoSurf	✓	✗ [a]	✓	✗	✗	✗	✓	✗	✓
EpiSurf	✓	✗ [a]	✓	✗	✗	✗	✓	✗	✓
OMeta	✓	✗ [a]	✗	✓	✗	✗	✗	✗	✗
GEO	✗	✗	✓	✗	✗	✗	✓	✓	✓
RNASeqMetaDB	✗	✗	✗	✗	✗	✗	✓	✗	✗
LabPipe	✓	✓	✓	✓	✗	✗	✗	✗	✗
METAGENOTE	✓	✗	✗	✓	✗	✗	✗	✗	✓

Figure 4. ***Metadata management comparison chart.*** *PEPhub compares favorably to alternative metadata management systems. *a: While open-source, no clear documentation exists for self hosting an instance.*

Future development of PEPhub

We have several plans for PEPhub development. First, with some basic adapters, PEPhub could simplify the process of submitting data to public repositories, such as SRA or GEO. We are interested in working with interested parties to explore how to simplify the data submission process. Second, we plan to extend PEPhub to serve as a source for data analysis. We already have adopted our pipeline engine, looper, to retrieve sample tables from PEPhub. We plan to extend this functionality to other pipeline engines, such as Snakemake. Finally, PEPhub has potential to serve as a pipeline management dashboard, wherein processes could send updates to the server as pipelines run. We are currently developing software called `pipestat`, which provides a standardized mechanism for pipelines to report results, and we are exploring ways to link PEPhub with running

pipelines such that they can be monitored directly from the web interface.

Methods: Implementation and deployment details

FastAPI web service

The PEPhub server is built with FastAPI, a web framework optimized for speed and high-performance. FastAPI is specifically designed for developing APIs. We chose FastAPI for its automatic data validation capabilities, built-in API documentation, and because using Python allows us to interface with existing Python infrastructure for metadata management we developed previously [24]. The FastAPI application uses our companion package `pepdbagent` to interface with a Postgres database. The user interface is built using React.js and TypeScript, and is packaged with the server.

`pepdbagent` companion package

To manage project creation, fetching, deletion and insertion into the database, we developed a companion package called `pepdbagent`. `pepdbagent` acts as a simple wrapper around the popular Python object-relational mapper (ORM) `SQLAlchemy` to provide a convenient API for managing projects in our database. Both PEPhub itself and all maintenance scripts use `pepdbagent` to manage the PEPs stored inside the database.

PostgreSQL database

Postgres is well-suited for storing the structured and unstructured data found in PEPs because it excels at both relational and document storage. The PEPhub database is comprised of three tables. The first table, `projects` stores the PEPs metadata and PEPs configuration. The `projects` table consists of twelve columns to store data like the project timestamp, project id, and the project configuration as a JSON blob. The other two tables: `samples`, and `subsamples` store samples and subsamples that are linked with `project.id` to a specific project in the `project` table. We host the public PEPhub database instance on the Amazon Web Services Relational Database Service (AWS RDS).

PEPhubClient Python and command-line interface

To facilitate command-line interaction and third-party tools using PEPhub, we have developed PEPhubClient. PEPhubClient is a command-line interface and Python API that leverages the machine-oriented interfaces of PEPhub. Namely, the public API. PEPhubClient makes it easy to push and pull PEPs to any PEPhub instance. The command-line interface supports authentication to make authorized requests. This includes working with private PEPs, downloading PEPs, editing PEPs, and submitting PEPs. The PEPhubClient CLI is implemented in Python using the `typer` library.

Containers for custom deployment

To standardize deployment and promote interoperability, we've packaged the PEPhub server and database as docker containers. These containers are made available on dockerhub. This makes it easy to launch your own instance of PEPhub.

Populating PEPhub with biological sample metadata from GEO

To populate PEPhub, we developed a pipeline to ingest sample metadata from GEO. Our pipeline uploads PEPs from GEO in two steps. First, it identifies experiments that were added or updated in certain period of time to the Gene Expression Omnibus[7] using the GEOfetch Python API [21]. Second, GEOfetch downloads, formats and produces PEPs from GEO experiments that are later uploaded to the PEPhub database. Our database now stores more than 150,000 high-throughput sample tables from the last 10 years from Gene Expression Omnibus. We developed a pipeline that uses GitHub `schedule` actions to automate the download, formatting, and upload or re-upload of new project releases on GEO. Moreover, the pipeline includes an automatic check for successful previous uploads, ensuring that all GEO projects are consistently updated on PEPhub without the need for manual intervention.

Natural language search

To support the text mining and embedding pipeline, we developed a companion tool called `pepembed` that embeds a database of PEPs and inserts them into a Qdrant database instance. For each PEP in the database `pepembed` does three things: first, it *flattens* the `yaml` representation of the sample metdata to create a continuous string; second, it utilizes an embedding model (e.g. sentence transformer) to produce a low-dimensional vector representation of this text; finally, `pepembed` will insert this vector in a Qdrant instance along with that PEPs namespace, name, and tag. We leverage GitHub actions to run indexing tasks periodically to ensure that all PEPs stay properly indexed, even if their data change. `pepembed` is open-source and available on GitHub: https://github.com/pepkit/pepembed.

Authentication and authorization

PEPhub supports two authentication flows: authorization code flow and device code flow. Both take advantage of GitHub's OAuth services. In addition, both authentication flows require users to login with GitHub via a web browser, upon which a code is returned. This code is then exchanged for a JSON Web Token (JWT) via a `POST` request which can be used to make subsequently authorized requests. While very similar, both flows exist to make it as easy as possible to integrate third-party software with a PEPhub instance.

Funding

This work was supported by the National Institute of General Medical Sciences grant R35-GM128636 (NCS) and National Human Genome Research Institute grant R01-HG012558 (NCS). Funders had no role in study design, data collection, analysis, or publication.

Availability and requirements

Project name: PEPhub
Project home page: https://pephub.databio.org
Operating system: Platform independent
Programming language: Python
License: BSD-2
bio.tools ID: pephub
SciCrunch RRID: SCR_024892

Conflict of interest statement

NCS is a consultant for InVitro Cell Research, LLC.

References

1. Volchenboum SL, Cox SM, Heath A, Resnick A, Cohn SL, Grossman R. Data commons to support pediatric cancer research. American Society of Clinical Oncology Educational Book. 2017;37:746–52. doi:10.14694/edbk_175029.

2. Bui AAT, Van Horn JD, Consortium NBC. Envisioning the future of 'big data' biomedicine. J Biomed Inform. 2017;69:115–7. doi:10.1016/j.jbi.2017.03.017.

3. Armit C, Tuli MA, Hunter CI. A decade of GigaScience: GigaDB and the open data movement. GigaScience. 2022;11. doi:10.1093/gigascience/giac053.

4. Xue B, Khoroshevskyi O, Gomez RA, Sheffield NC. Opportunities and challenges in sharing and reusing genomic interval data. Frontiers in Genetics. 2023;14. doi:10.3389/fgene.2023.1155809.

5. Wilkinson MD, Dumontier M, Aalbersberg IjJ, Appleton G, Axton M, Baak A, et al. The FAIR Guiding Principles for scientific data management and stewardship. Scientific Data. 2016;3:160018. doi:10.1038/sdata.2016.18.

6. Sheffield NC, Bonazzi VR, Bourne PE, Burdett T, Clark T, Grossman RL, et al. From biomedical cloud platforms to microservices: Next steps in FAIR data and analysis. Scientific Data. 2022;9:553. doi:10.1038/s41597-022-01619-5.

7. Edgar R, Domrachev M, Lash AE. Gene Expression Omnibus: NCBI gene expression and hybridization array data repository. Nucleic Acids Research. 2002;30:207–10.

8. Sloan CA, Chan ET, Davidson JM, Malladi VS, Strattan JS, Hitz BC, et al. ENCODE data at the ENCODE portal. Nucleic Acids Research. 2016;44:D726–32. doi:10.1093/nar/gkv1160.

9. Bourne PE, Bonazzi V, Dunn M, Green ED, Guyer M, Komatsoulis G, et al. The NIH Big Data to Knowledge (BD2K) initiative. Journal of the American Medical Informatics Association : JAMIA. 2015;22:1114. doi:10.1093/jamia/ocv136.

10. Leipzig J, Nüst D, Hoyt CT, Ram K, Greenberg J. The role of metadata in reproducible computational research. Patterns. 2021;2:100322. doi:10.1016/j.patter.2021.100322.

11. Sheffield N, LeRoy N, Khoroshevskyi O. Challenges to sharing sample metadata in computational genomics. Frontiers in Genetics. 2023;14.

12. Canakoglu A, Bernasconi A, Colombo A, Masseroli M, Ceri S. GenoSurf: Metadata driven semantic search system for integrated genomic datasets. Database. 2019;2019:baz132. doi:10.1093/database/baz132.

13. Serna Garcia G, Leone M, Bernasconi A, Carman MJ. GeMI: Interactive interface for transformer-based Genomic Metadata Integration. Database. 2022;2022:baac036. doi:10.1093/database/baac036.

14. Masseroli M, Pinoli P, Venco F, Kaitoua A, Jalili V, Palluzzi F, et al. GenoMetric Query Language: A novel approach to large-scale genomic data management. Bioinformatics. 2015;31:1881–8. doi:10.1093/bioinformatics/btv048.

15. Davis S, Meltzer PS. GEOquery: A bridge between the Gene Expression Omnibus (GEO) and BioConductor. Bioinformatics. 2007;23:1846–7. doi:10.1093/bioinformatics/btm254.

16. Quiñones M, Liou DT, Shyu C, Kim W, Vujkovic-Cvijin I, Belkaid Y, et al. "METAGENOTE: A simplified web platform for metadata annotation of genomic samples and streamlined submission to NCBI's sequence read archive." BMC Bioinformatics. 2020;21:378. doi:10.1186/s12859-020-03694-0.

17. Cappelli E, Cumbo F, Bernasconi A, Canakoglu A, Ceri S, Masseroli M, et al. OpenGDC: Unifying, Modeling, Integrating Cancer Genomic Data and Clinical Metadata. Applied Sciences. 2020;10:6367. doi:10.3390/app10186367.

18. Bernasconi A, Cilibrasi L, Al Khalaf R, Alfonsi T, Ceri S, Pinoli P, et al. EpiSurf: Metadata-driven search server for analyzing amino acid changes within epitopes of SARS-CoV-2 and other viral species. Database. 2021;2021:baab059. doi:10.1093/database/baab059.

19. Singh I, Kuscuoglu M, Harkins DM, Sutton G, Fouts DE, Nelson KE. OMeta: An ontology-based, data-driven metadata tracking system. BMC bioinformatics. 2019;20:8. doi:10.1186/s12859-018-2580-9.

20. Sheffield NC, Stolarczyk M, Reuter VP, Rendeiro AF. Linking big biomedical datasets to modular analysis with Portable Encapsulated Projects. 2021;2020.10.08.331322. doi:10.1101/2020.10.08.331322.

21. Khoroshevskyi O, LeRoy N, Reuter VP, Sheffield NC. GEOfetch: A command-line tool for downloading data and standardized metadata from GEO and SRA. Bioinformatics. 2023;btad069. doi:10.1093/bioinformatics/btad069.

22. Malkov YA, Yashunin DA. Efficient and robust approximate nearest neighbor search using Hierarchical Navigable Small World graphs. 2018. doi:10.48550/arXiv.1603.09320.

23. Guo Z, Tzvetkova B, Bassik JM, Bodziak T, Wojnar BM, Qiao W, et al. RNASeqMetaDB: A database and web server for navigating metadata of publicly available mouse RNA-Seq datasets. Bioinformatics (Oxford, England). 2015;31:4038–40. doi:10.1093/bioinformatics/btv503.

24. Sheffield NC, Stolarczyk M, Reuter VP, Rendeiro AF. Linking big biomedical datasets to modular analysis with Portable Encapsulated Projects. 2021;2020.10.08.331322. doi:10.1101/2020.10.08.331322.

Supplemental figures

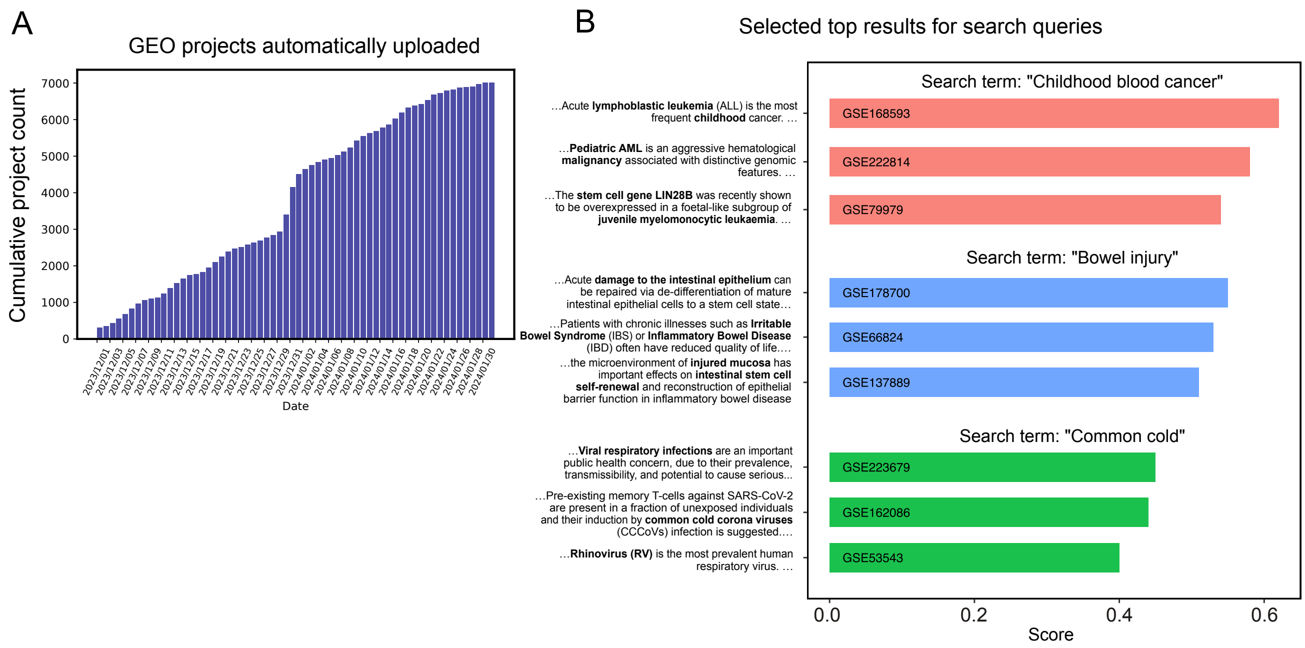

Supplementary Figure S1. Analysis of PEP metadata and search. *A) Barchart showing the cumulative number of new biological sample tables added to PEPhub from GEO, automatically. PEPhub automatically indexed more than 1000 new projects during this 3-week demo span. B) Illustrative search result scores selected from the top 10 responses returned by the PEPhub semantic search engine for biological search terms shown*

Supplemental text

Search result examples

Illustrative search results from PEPhub. Search results from GEO and PEPhub for common queries. The PEPhub search engine returns results that are more relevant to the query than the GEO search engine. Moreover, the results returned by PEPhub are more diverse.

Search term: "Childhood blood cancer"

GEO results:

- . . . effects of MZ1 on multiple molecular subtypes of B-cell acute lymphoblastic leukemia cells (GSE217540)
- . . . PAF1 and FACT to drive high density enhancer interactions in leukemia (GSE202451)
- . . . PAF1 and FACT to drive high density enhancer interactions in leukemia. . . (GSE202450)
- . . . PAF1 and FACT to drive high density enhancer interactions in leukemia. . . (GSE202449)

PEPhub results:

- . . . genomic alterations in radiation-related breast cancer among childhood cancer survivors. . . (GSE62940)
- . . . analysis of CD10+/CD19+ pre B lymphoblasts from bone marrow and peripheral blood of B-ALL patients (GSE168593)
- . . . DNA methylation profiling predicts relapse in childhood B-cell acute lymphoblastic leukemia (GSE39141)
- . . . analysis of pediatric histiocytic sarcomas and anteceding hematologic malignancies (GSE109904)

Search term: "Bowel injury"

GEO results:

- tumors from mice fed diets excluding methionine/tryptophan/niacin (GSE246627)
- tumors from mice fed diets excluding methionine/tryptophan/niacin (GSE246626)
- Airway Microfold (M) Cells Emerge in the Post-IAV Lung (GSE244279)

PEPhub results:

- Colonic mucosal injury responses (GSE164918)

Search term: “Common cold”

GEO results:

- ATAC-Seq of Batf-deficient pDC Transcriptomes... (GSE178410)
- Deterministic reprogramming of neutrophils in tumors (GSE244536)
- ...Pancreatic Tumors Reveal Distinct Compartmentalisation of Neutrophil Subsets (GSE244534)
- ...of neutrophil subsets in a mouse model of pancreatic cancer (GSE244531)

PEPhub results:

- A longitudinal study of natural respiratory viral infections (GSE223679)
- ...temperature variation controls pre-mRNA processing and transcription of anti-viral genes (GSE193639)
- Influenzavirus serotype association to global whole blood transcriptional changes (GSE29385)
- The immune response and microbiota profiles during co-infection with P. vivax... (GSE144792)

Response to PEPhub reviews

Editor's comments

Your manuscript "PEPhub: a database, web interface, and API for editing, sharing, and validating biological sample metadata" (GIGA-D-23-00246) has been assessed by our reviewers. Although it is of interest, we are unable to consider it for publication in its current form. The reviewers have raised a number of points which we believe would improve the manuscript and may allow a revised version to be published in GigaScience.

Their reports, together with any other comments, are below. Please also take a moment to check our website at https://www.editorialmanager.com/giga/ for any additional comments that were saved as attachments.

In addition, please register any new software application in the bio.tools and SciCrunch.org databases to receive RRID (Research Resource Identification Initiative ID) and biotoolsID identifiers, and include these in your manuscript. Computational workflows should be registered in workflowhub.eu and the DOIs cited in the relevant places in the manuscript. These will facilitate tracking, reproducibility and re-use of your tool.

. . .

Please include a point-by-point within the 'Response to Reviewers' box in the submission system. Please ensure you describe additional experiments that were carried out and include a detailed rebuttal of any criticisms or requested revisions that you disagreed with. Please also ensure that your revised manuscript conforms to the journal style, which can be found in the Instructions for Authors on the journal homepage. If the data and code has been modified in the revision process please be sure to update the public versions of this too.

> *Dear Hongling,*
>
> *We thank you for your feedback and for the helpful comments of the reviewers. We would now like to submit a revised version of our manuscript for your review. We have addressed all your comments, as well as the comments of the reviewers, and included a point-by-point response below.*
>
> *The Biotools ID is pephub, and SciCrunch.org database ID is SCR_024892. We have added these to the manuscript.*
>
> *We appreciate the opportunity to revise our manuscript and how you will find these revisions satisfactory.*
>
> *Thank you,*
> *The authors*

Reviewer #1

Metadata describes the who, what, where, when, and why of an experiment. Sample metadata is arguably the most important of these, but not the only type. LeRoy et al describes a user-centric sample metadata management system with extensibility, support for multiple interface modalities, and fuzzy semantic search.

This system and portal, PEPHub, bridges the gaps between LIMS, which are tightly bound to the wet lab, metadata fetchers like GEOfetch (from the same group) or pysradb, and public portals like MetaSRA and the others listed in . Then and both of which don't allow you to roll your own portal internally, and whose search criteria are not fuzzy or semantic.

People have been storing metadata in bespoke databases for decades, but not in an interoperable mature fashion. The PepHUB portal builds on some existing Pep standards by the same group, introducing a restful API and GUI.

I find this paper a novel and compelling submission but would like the following minor revisions:

1. Typically in SRA a sample refers to a dna sample drawn from a tissue sample (ie BioSample) and then runs describe sequencing attempts on those dna samples, and files are produced from each of the runs. It is unclear to me how someone working in an internal lab using PEPHub would know how to extract the file locations of sequence files associated with a sample if these are many-to-one. In the GEO example provided I can click on the SRX link to see the runs and files but how would this work for an internally generated entry? I need the authors to explain this either as a response or in the text.

   PEP accommodates many-to-one sample attributes, which are called 'subsamples' in PEP parlance. These attributes can be edited in the 'Subsample' tab, and are available as objects on the samples retrieved from the PEPhub API. So, basically, you would just choose an attribute for file locations, and these file paths would go under subsamples. The PEPhub API correctly provides these as a list of values for the corresponding attribute. Users may thus use these attributes in a way similar to attributes with a single value, it's just that the attribute value will be an object. Alternatively, PEPhub also provides 'filters' that allow a user to retrieve these as independent rows in a table, but we don't recommend this approach. A section in the PEP documentation discusses this here: https://pep.databio.org/spec/howto-multi-value-attributes/

   To clarify this, we've added a sentence to the manuscript to reference attributes with multiple values.

2. I think the paper has to briefly describe how the authors envision how PEPhub should interface with or replaces a LIMS for labs that are producing their own data and describe how it can help accelerate the SRA submission process for these data generating labs.

   Good point! Indeed, PEPhub does share some similarities with a LIMS, though it comes from a different angle than a typical LIMS. We added the following paragraph to the mansucript comparing PEPhub to LIMS:

   PEPhub may also be compared to a Laboratory Information Management System (LIMS), a broad term with several interpretations. One difference is that LIMS tend to target management challenges related to physical sample handling in a wet lab, such as connecting to machinery, ordering reagents, and tracking physical samples through an experimental protocol. PEPhub could fulfill some of these functions, but in general, PEPhub targets a later phase of the experimental process: after data is generated, and the samples need to be analyzed and shared. PEPhub's strengths are the ability to share data easily, in a more universal form, a public API, standardization, and searching. Thus, many cases, it may make sense for PEPhub to live alongside a LIMS.

3. Change "Bernasconi2021" to META-BASE in the text

   Thanks for catching this error. We replaced it with EpiSurf*, the tool this paper refers to.*

4. Some of the search confidence measures show an absurd level of significant digits (e.g. 56.99999999999999% Please round that as it's only used for sorting.

   Good point, we have fixed this so it's much cleaner. This is done in the most recent version of the software.

Reviewer #2:

This manuscript by LeRoy et al. introduces PEPhub, a database aimed at enhancing the sharing and interoperability of biological metadata using the PEP framework. One of the key highlights of this manuscript is the visualization of the PEP framework, which improves the adoption of the PEP framework, facilitating the reuse of metadata. Additionally, PEPhub integrates data from GEO, making it convenient for users to access and utilize. Furthermore, PEPhub offers metadata validation, allowing users to quickly compare their PEP with other PEPhub schemas. Another notable feature is the natural language search, which further enhances the user experience. Overall, PEPhub provides a comprehensive solution that promotes efficient metadata sharing, while leveraging the impact of the PEP framework in organizing large-scale biological research projects.

While this manuscript was interesting to read, I have several concerns regarding its "semantic" search system and the interaction of PEPHub.

1. The authors mentioned their use of a tool called "pepembed" to embed PEP descriptions into vectors. However, I was unable to locate the tool on GitHub, and there is limited information in the Method section regarding this. Could the authors provide additional details regarding the process of embedding vectors?

   We have made the `pepembed` tool public and it can be found at https://github.com/pepkit/pepembed. In addition, we have expanded the methods section for PEP embeddings to provide more clarity on the embedding process. We also added documentation on pepembed to the new PEPhub documentation website, which can be found at https://pep.databio.org/pephub.

2. The authors implemented semantic search as an advantage of PEPhub. However, they did not evaluate the effectiveness of their natural language search engine, such as assessing accuracy, recall rate, or F1 score. It would be beneficial for the authors to perform an evaluation of their natural language search engine and provide metrics to demonstrate its performance. This would enhance the credibility and reliability of their claims regarding the advantages of natural language search in PEPhub.

   Thank you for raising this concern. You're right that it would be useful to show evaluation to show why we're happy with the sentence transformer. To address this, we have added to the paper some example searches and what the searches yield. This will better showcase the search engine's capabilities. Specifically, we added 1) a new supplemental figure depicting different search queries and relevant results, with the score from the PEPhub vector search; and 2) a table comparing some results from our semantic search system with GEO's native search engine. While subjective, this table shows that our search system returns relevant results while also being more diverse. The table is reproduced here:

**Query**	**GEO result descriptions**	**PEPhub result descriptions**
"Childhood blood cancer"	...effects of MZ1 on multiple molecular subtypes of B-cell acute lymphoblastic leukemia cells (GSE217540)	...genomic alterations in radiation-related breast cancer among childhood cancer survivors... (GSE62940)
	...PAF1 and FACT to drive high density enhancer interactions in leukemia (GSE202451)	...analysis of CD10+/CD19+ pre B lymphoblasts from bone marrow and peripheral blood of B-ALL patients (GSE168593)
	...PAF1 and FACT to drive high density enhancer interactions in leukemia... (GSE202450)	...DNA methylation profiling predicts relapse in childhood B-cell acute lymphoblastic leukemia (GSE39141)

Query	GEO result descriptions	PEPhub result descriptions
	. . . PAF1 and FACT to drive high density enhancer interactions in leukemia. . . (GSE202449)	. . . analysis of pediatric histiocytic sarcomas and anteceding hematologic malignancies (GSE109904)
"Bowel injury"	tumors from mice fed diets excluding methionine/tryptophan/niacin (GSE246627)	Colonic mucosal injury responses (GSE164918)
	tumors from mice fed diets excluding methionine/tryptophan/niacin (GSE246626)	
	Airway Microfold (M) Cells Emerge in the Post-IAV Lung (GSE244279)	
"Common cold"	ATAC-Seq of Batf-deficient pDC Transcriptomes. . . (GSE178410)	A longitudinal study of natural respiratory viral infections (GSE223679)
	Deterministic reprogramming of neutrophils in tumors (GSE244536)	. . . temperature variation controls pre-mRNA processing and transcription of anti-viral genes (GSE193639)
	. . . Pancreatic Tumors Reveal Distinct Compartmentalisation of Neutrophil Subsets (GSE244534)	Influenzavirus serotype association to global whole blood transcriptional changes (GSE29385)
	. . . of neutrophil subsets in a mouse model of pancreatic cancer (GSE244531)	The immune response and microbiota profiles during co-infection with P. vivax. . . (GSE144792)

Given that PEPhub is a user, rather than a creator of an NLP model, the novelty of PEPhub is in the engineering approach – the application of the NLP model. Our natural language search leverages an existing sentence transformer that has been published and evaluated elsewhere by the NLP community, and to some degree we are relying on this evaluation in our selection of the model. We can switch this model whenever a better one is made – our contribution is really in the deployment of the model, which is part of PEPhub. Therefore, a more comprehensive analysis of this particular model is outside the scope of a manuscript describing PEPhub. To better reflect this emphasis and what we view as the contribution of PEPhub, we also reduced the amount of text in the manuscript discussing the advantages of the semantic search.

3. It would be more beneficial to include the metadata in the search system rather than solely relying on the project description. For instance, when I searched for SRX17165287 (https://pephub.databio.org/geo/gse211736?tag=default), no results were returned.

This is a good point; you are right, it is not possible to search for sample names and get exact matches. To address this, we made three updates. First, we made changes to Qdrant vector database to return exact string text matches when performing semantic search. Second, we made provisions to bubble-up hits that contained these exact matches. Third, we updated the web interface to allow users to search through project names and descriptions. We recommend that if the user is searching for a specific PEP in a namespace that they use the search box provided on the /{namespace} page as this performs a more conventional SQL-based search on the project titles and descriptions. These have improved the search function in our hands.

However, even with these changes, it's still not possible to search for an exact name of a particular sample name *within a project, which is what your original example showed. We agree with that an ideal system would allow users to find exact matches to sample metadata*

like this. We have thought of approaches that would enable this, but they would all explode the resource requirements of the server. Our current approach uses only a tiny amount of resources for a very powerful search; we are running the API on an ECS instance, consuming <25% of a virtual CPU and <1GB of ram. Our vector search is running on a free-tier qdrant service, consuming < 50% of a vCPU and 1GB of RAM. Given that this project has limited resources, keeping the cost to almost nothing will allow us to maintain PEPhub into the future. But despite this low cost, we are extremely happy with the quality of the search – even though this search isn't perfect, it is useful. We are working on other solutions that will improve the search further, and we're trying to come up with a way to make exact matching of sample names possible, but in the short term, we can't figure out how to do this without expanding the resource requirements beyond what we're capable of sustaining.

4. When creating a new PEP, it appears that I can submit two samples with identical values. According to the PEP framework guidelines, it is mentioned that "Typically, samples should have unique values in the sample table index column". Therefore, the authors should enhance their metadata validation system to enforce this uniqueness constraint. Additionally, if I enter two identical values in the sample field and then attempt to add a SUBSAMPLE, an error occurs. However, when I modify one of the samples, I am able to save it successfully.

   In fact, this is the correct behavior. In the PEP 2.1.0 specification, within the sample_table, users can provide multiple rows with the same sample_name. These are combined into a single sample in the initialized PEP. This is a way to provide attributes with multiple values, and the provided columns are treated as subsamples. In contrast, if a user provides subsamples, then *the sample_table must contain only unique values, because subsamples will be provided in subsamples table. So, though this was unintuitive, the system is actually behaving correctly. There were some poorly worded sections of the PEP documentation, which we have corrected. We revised the section completely, which now makes it more clear how the multi-value attribute works. To see the corrected documentation, please visit: - https://pep.databio.org/spec/specification/#subsample-table-specification - https://pep.databio.org/spec/howto-multi-value-attributes/*

5. The error messages should provide more specific guidance. Currently, when attempting to save metadata with an incorrect format, all error messages are displayed as: "Unknown error occurred: Unknown".

   Thank you for pointing our the lack of good error messaging. We have updated our error messages to provide much more informative responses that help users quickly diagnose their problems.

6. PEPhub should consider providing user guidelines or examples on how to fill in subsample metadata and any relevant rules associated with it.

   Thanks for the suggestion. We have tried to clean up the documentation and the connection between PEP and PEPhub with a new documentation framework, and improved organization and links to that documentation from PEPhub. This includes new PEPhub-specific instructions that articulate how to use the web appliation, and improvements on the PEP specification itself.

7. In the Validation module, what are the rules for validation? Does it only check for the required column names in the schema, or does it also validate the content of the metadata, such as whether the metadata is in the correct format (e.g., int or string)? Additionally, it would be beneficial to provide an option to download the relevant schema and clearly specify the required column names in the schema. This would enable users to better organize their PEP to comply with the schema format and ensure that their metadata is accurately validated.

Thank you for pointing out a clear gap in our application! We had no explanation for the validator as well as no way to download schema one wishes to validate against. We've addressed this in two ways: 1) we added a description to the validator form, and 2) we now allow users to download the schema as a `yaml` file.

8. This version of PEPHub primarily focuses on metadata. Have the authors considered any plans to expand this database to include data/pipeline management within the PEP framework? It would be valuable for the authors to discuss their future plans for PEPHub in this manuscript.

   Indeed, this is a good idea, and definitely something we have been thinking about and planning for. We are working on ideas for this and intend this to be the direction for PEPhub. We added a future plan section to the mansucript, which says this:

   We have several plans for PEPhub development. First, with some basic adapters, PEPhub could simplify the process of submitting data to public repositories, such as SRA or GEO. We are interested in working with interested parties to explore how to simplify the data submission process. Second, we plan to extend PEPhub to serve as a source for data analysis. We already have adopter our pipeline engine, looper, to retrieve sample tables from PEPhub. We plan to extend this functionality to other pipeline engines, such as Snakemake. Finally, PEPhub has potential to serve as a pipeline management dashboard, wherein processes could send updates to the server as pipelines run. We are exploring ways to link PEPhub with running pipelines such that they can be monitored directly from the web interface.

Some minor concerns:

1. When searching for content within a specific namespace, it would be beneficial for the pagination bar at the bottom of the webpage to display the number of pages. Now there are only Previous/Next buttons.

   To address this gap, we've added a "number of results" in our pagination components on the namespace page.

2. As a web service, it is better to show the supporting browsers, such as Google Chrome (version xxx and above), Firefox (version xxx and above). I failed to open PEPHub website using an old version of Chrome.

   Good point. We have added a link on the about page specifying compatible browsers. This page will show which browser versions will work with pephub: https://browsersl.ist/#q=defaults – PEPhub is currently usable by >88% of a global audience.
