# Supplementary material for: PEPhub: a database, web interface, and API for editing, sharing, and validating biological sample metadata
Source: Gigascience. 2024 Jul 11;13:giae033. doi: 10.1093/gigascience/giae033 (PMC11238423; doi:10.1093/gigascience/giae033)
Supplement: giae033_GIGA-D-23-00246_Revision_3 [file giae033_giga-d-23-00246_revision_3.pdf]

GigaScience

PEPhub: a database, web interface, and API for editing, sharing, and validating biological sample metadata

--Manuscript Draft--

**Manuscript Number:**	GIGA-D-23-00246R3
**Full Title:**	PEPhub: a database, web interface, and API for editing, sharing, and validating biological sample metadata
**Article Type:**	Technical Note
**Funding Information:**	NIGMS (GM128636) — Dr. Nathan C. Sheffield<br>National Human Genome Research Institute (R01-HG012558) — Dr. Nathan C. Sheffield
**Abstract:**	Background<br><br>As biological data increases, we need additional infrastructure to share it and promote interoperability. While major effort has been put into sharing data, relatively less emphasis is placed on sharing metadata. Yet, sharing metadata is also important, and in some ways has a wider scope than sharing data itself.<br><br>Results<br><br>Here, we present PEPhub, an approach to improve sharing and interoperability of biological metadata. PEPhub provides an API, natural language search, and user-friendly web-based sharing and editing of sample metadata tables. We used PEPhub to process more than 100,000 published biological research projects and index them with fast semantic natural language search. PEPhub thus provides a fast and user-friendly way to finding existing biological research data, or to share new data.<br><br>Availability<br><br>https://pephub.databio.org
**Corresponding Author:**	Nathan C. Sheffield<br><br>UNITED STATES
**Corresponding Author Secondary Information:**	
**Corresponding Author's Institution:**	
**Corresponding Author's Secondary Institution:**	
**First Author:**	Nathan J. LeRoy
**First Author Secondary Information:**	
**Order of Authors:**	Nathan J. LeRoy<br>Oleksandr Khoroshevskyi<br>Aaron O’Brien<br>Rafal Stepien<br>Alip Arslan<br>Nathan C. Sheffield
**Order of Authors Secondary Information:**	
**Response to Reviewers:**	ok

Powered by Editorial Manager® and ProduXion Manager® from Aries Systems Corporation

Additional Information:	
**Question**	**Response**
Are you submitting this manuscript to a special series or article collection?	No
**Experimental design and statistics**<br><br>Full details of the experimental design and statistical methods used should be given in the Methods section, as detailed in our Minimum Standards Reporting Checklist. Information essential to interpreting the data presented should be made available in the figure legends.<br><br>Have you included all the information requested in your manuscript?	No
If not, please give reasons for any omissions below.<br><br>as follow-up to "**Experimental design and statistics**<br><br>Full details of the experimental design and statistical methods used should be given in the Methods section, as detailed in our Minimum Standards Reporting Checklist. Information essential to interpreting the data presented should be made available in the figure legends.<br><br>Have you included all the information requested in your manuscript?<br><br>"	No experimental design or statistical methods are used.
**Resources**<br><br>A description of all resources used, including antibodies, cell lines, animals and software tools, with enough information to allow them to be uniquely identified, should be included in the Methods section. Authors are strongly encouraged to cite Research Resource	Yes

Identifiers (RRIDs) for antibodies, model organisms and tools, where possible.<br><br>Have you included the information requested as detailed in our Minimum Standards Reporting Checklist?	
**Availability of data and materials**<br><br>All datasets and code on which the conclusions of the paper rely must be either included in your submission or deposited in publicly available repositories (where available and ethically appropriate), referencing such data using a unique identifier in the references and in the "Availability of Data and Materials" section of your manuscript.<br><br>Have you have met the above requirement as detailed in our Minimum Standards Reporting Checklist?	Yes

Click here to access/download;LaTeX - Main Document (TeX file);manuscript_2024-05-10.tex

```
This is pdfTeX, Version 3.141592653-2.6-1.40.25 (TeX Live 2023)
(preloaded format=pdflatex 2024.3.8)  21 MAY 2024 01:40
entering extended mode
 restricted \write18 enabled.
 %&-line parsing enabled.
**manuscript_2024-05-10.tex
(./manuscript_2024-05-10.tex
LaTeX2e <2023-11-01> patch level 1
L3 programming layer <2024-02-20>
(c:/texlive/2023/texmf-dist/tex/latex/base/article.cls
Document Class: article 2023/05/17 v1.4n Standard LaTeX document class
(c:/texlive/2023/texmf-dist/tex/latex/base/size10.clo
File: size10.clo 2023/05/17 v1.4n Standard LaTeX file (size option)
)
\c@part=\count188
\c@section=\count189
\c@subsection=\count190
\c@subsubsection=\count191
\c@paragraph=\count192
\c@subparagraph=\count193
\c@figure=\count194
\c@table=\count195
\abovecaptionskip=\skip48
\belowcaptionskip=\skip49
\bibindent=\dimen140
) (c:/texlive/2023/texmf-dist/tex/latex/geometry/geometry.sty
Package: geometry 2020/01/02 v5.9 Page Geometry
(c:/texlive/2023/texmf-dist/tex/latex/graphics/keyval.sty
Package: keyval 2022/05/29 v1.15 key=value parser (DPC)
\KV@toks@=\toks17
) (c:/texlive/2023/texmf-dist/tex/generic/iftex/ifvtex.sty
Package: ifvtex 2019/10/25 v1.7 ifvtex legacy package. Use iftex instead.
(c:/texlive/2023/texmf-dist/tex/generic/iftex/iftex.sty
Package: iftex 2022/02/03 v1.0f TeX engine tests
))
\Gm@cnth=\count196
\Gm@cntv=\count197
\c@Gm@tempcnt=\count198
\Gm@bindingoffset=\dimen141
\Gm@wd@mp=\dimen142
\Gm@odd@mp=\dimen143
\Gm@even@mp=\dimen144
\Gm@layoutwidth=\dimen145
\Gm@layoutheight=\dimen146
\Gm@layouthoffset=\dimen147
\Gm@layoutvoffset=\dimen148
\Gm@dimlist=\toks18
) (c:/texlive/2023/texmf-dist/tex/latex/algorithms/algorithm.sty
Package: algorithm 2009/08/24 v0.1 Document Style `algorithm' - floating
enviro
nment
(c:/texlive/2023/texmf-dist/tex/latex/float/float.sty
Package: float 2001/11/08 v1.3d Float enhancements (AL)
\c@float@type=\count199
```

```
\float@exts=\toks19
\float@box=\box51
\@float@everytoks=\toks20
\@floatcapt=\box52
) (c:/texlive/2023/texmf-dist/tex/latex/base/ifthen.sty
Package: ifthen 2022/04/13 v1.1d Standard LaTeX ifthen package (DPC)
)
\@float@every@algorithm=\toks21
\c@algorithm=\count266
) (c:/texlive/2023/texmf-dist/tex/latex/algorithmicx/algpseudocode.sty
Package: algpseudocode
(c:/texlive/2023/texmf-dist/tex/latex/algorithmicx/algorithmicx.sty
Package: algorithmicx 2005/04/27 v1.2 Algorithmicx
Document Style algorithmicx 1.2 - a greatly improved `algorithmic' style
\c@ALG@line=\count267
\c@ALG@rem=\count268
\c@ALG@nested=\count269
\ALG@tlm=\skip50
\ALG@thistlm=\skip51
\c@ALG@Lnr=\count270
\c@ALG@blocknr=\count271
\c@ALG@storecount=\count272
\c@ALG@tmpcounter=\count273
\ALG@tmplength=\skip52
)
Document Style - pseudocode environments for use with the `algorithmicx'
style
) (c:/texlive/2023/texmf-dist/tex/generic/iftex/ifxetex.sty
Package: ifxetex 2019/10/25 v0.7 ifxetex legacy package. Use iftex
instead.
) (c:/texlive/2023/texmf-dist/tex/generic/iftex/ifluatex.sty
Package: ifluatex 2019/10/25 v1.5 ifluatex legacy package. Use iftex
instead.
) (c:/texlive/2023/texmf-dist/tex/latex/hyperref/hyperref.sty
Package: hyperref 2024-01-20 v7.01h Hypertext links for LaTeX
(c:/texlive/2023/texmf-dist/tex/latex/kvsetkeys/kvsetkeys.sty
Package: kvsetkeys 2022-10-05 v1.19 Key value parser (HO)
) (c:/texlive/2023/texmf-dist/tex/generic/kvdefinekeys/kvdefinekeys.sty
Package: kvdefinekeys 2019-12-19 v1.6 Define keys (HO)
) (c:/texlive/2023/texmf-dist/tex/generic/pdfescape/pdfescape.sty
Package: pdfescape 2019/12/09 v1.15 Implements pdfTeX's escape features
(HO)
(c:/texlive/2023/texmf-dist/tex/generic/ltxcmds/ltxcmds.sty
Package: ltxcmds 2023-12-04 v1.26 LaTeX kernel commands for general use
(HO)
) (c:/texlive/2023/texmf-dist/tex/generic/pdftexcmds/pdftexcmds.sty
Package: pdftexcmds 2020-06-27 v0.33 Utility functions of pdfTeX for
LuaTeX (HO
)
(c:/texlive/2023/texmf-dist/tex/generic/infwarerr/infwarerr.sty
Package: infwarerr 2019/12/03 v1.5 Providing info/warning/error messages
(HO)
)
Package pdftexcmds Info: \pdf@primitive is available.
```

```
Package pdftexcmds Info: \pdf@ifprimitive is available.
Package pdftexcmds Info: \pdfdraftmode found.
)) (c:/texlive/2023/texmf-dist/tex/latex/hycolor/hycolor.sty
Package: hycolor 2020-01-27 v1.10 Color options for hyperref/bookmark
(HO)
) (c:/texlive/2023/texmf-dist/tex/latex/auxhook/auxhook.sty
Package: auxhook 2019-12-17 v1.6 Hooks for auxiliary files (HO)
) (c:/texlive/2023/texmf-dist/tex/latex/hyperref/nameref.sty
Package: nameref 2023-11-26 v2.56 Cross-referencing by name of section
(c:/texlive/2023/texmf-dist/tex/latex/refcount/refcount.sty
Package: refcount 2019/12/15 v3.6 Data extraction from label references
(HO)
) (c:/texlive/2023/texmf-
dist/tex/generic/gettitlestring/gettitlestring.sty
Package: gettitlestring 2019/12/15 v1.6 Cleanup title references (HO)
(c:/texlive/2023/texmf-dist/tex/latex/kvoptions/kvoptions.sty
Package: kvoptions 2022-06-15 v3.15 Key value format for package options
(HO)
))
\c@section@level=\count274
) (c:/texlive/2023/texmf-dist/tex/latex/etoolbox/etoolbox.sty
Package: etoolbox 2020/10/05 v2.5k e-TeX tools for LaTeX (JAW)
\etb@tempcnta=\count275
)
\@linkdim=\dimen149
\Hy@linkcounter=\count276
\Hy@pagecounter=\count277
(c:/texlive/2023/texmf-dist/tex/latex/hyperref/pd1enc.def
File: pd1enc.def 2024-01-20 v7.01h Hyperref: PDFDocEncoding definition
(HO)
Now handling font encoding PD1 ...
... no UTF-8 mapping file for font encoding PD1
) (c:/texlive/2023/texmf-dist/tex/generic/intcalc/intcalc.sty
Package: intcalc 2019/12/15 v1.3 Expandable calculations with integers
(HO)
)
\Hy@SavedSpaceFactor=\count278
(c:/texlive/2023/texmf-dist/tex/latex/hyperref/puenc.def
File: puenc.def 2024-01-20 v7.01h Hyperref: PDF Unicode definition (HO)
Now handling font encoding PU ...
... no UTF-8 mapping file for font encoding PU
)
Package hyperref Info: Option `unicode' set `true' on input line 4062.
Package hyperref Info: Hyper figures OFF on input line 4179.
Package hyperref Info: Link nesting OFF on input line 4184.
Package hyperref Info: Hyper index ON on input line 4187.
Package hyperref Info: Plain pages OFF on input line 4194.
Package hyperref Info: Backreferencing OFF on input line 4199.
Package hyperref Info: Implicit mode ON; LaTeX internals redefined.
Package hyperref Info: Bookmarks ON on input line 4446.
\c@Hy@tempcnt=\count279
(c:/texlive/2023/texmf-dist/tex/latex/url/url.sty
\Urlmuskip=\muskip16
Package: url 2013/09/16  ver 3.4  Verb mode for urls, etc.
```

```
)
LaTeX Info: Redefining \url on input line 4784.
\XeTeXLinkMargin=\dimen150
(c:/texlive/2023/texmf-dist/tex/generic/bitset/bitset.sty
Package: bitset 2019/12/09 v1.3 Handle bit-vector datatype (HO)
(c:/texlive/2023/texmf-dist/tex/generic/bigintcalc/bigintcalc.sty
Package: bigintcalc 2019/12/15 v1.5 Expandable calculations on big
integers (HO
)
))
\Fld@menulength=\count280
\Field@Width=\dimen151
\Fld@charsize=\dimen152
Package hyperref Info: Hyper figures OFF on input line 6063.
Package hyperref Info: Link nesting OFF on input line 6068.
Package hyperref Info: Hyper index ON on input line 6071.
Package hyperref Info: backreferencing OFF on input line 6078.
Package hyperref Info: Link coloring OFF on input line 6083.
Package hyperref Info: Link coloring with OCG OFF on input line 6088.
Package hyperref Info: PDF/A mode OFF on input line 6093.
(c:/texlive/2023/texmf-dist/tex/latex/base/atbegshi-ltx.sty
Package: atbegshi-ltx 2021/01/10 v1.0c Emulation of the original atbegshi
package with kernel methods
)
\Hy@abspage=\count281
\c@Item=\count282
\c@Hfootnote=\count283
)
Package hyperref Info: Driver (autodetected): hpdftex.
(c:/texlive/2023/texmf-dist/tex/latex/hyperref/hpdftex.def
File: hpdftex.def 2024-01-20 v7.01h Hyperref driver for pdfTeX
(c:/texlive/2023/texmf-dist/tex/latex/base/atveryend-ltx.sty
Package: atveryend-ltx 2020/08/19 v1.0a Emulation of the original
atveryend pac
kage
with kernel methods
)
\Fld@listcount=\count284
\c@bookmark@seq@number=\count285
(c:/texlive/2023/texmf-dist/tex/latex/rerunfilecheck/rerunfilecheck.sty
Package: rerunfilecheck 2022-07-10 v1.10 Rerun checks for auxiliary files
(HO)
(c:/texlive/2023/texmf-dist/tex/generic/uniquecounter/uniquecounter.sty
Package: uniquecounter 2019/12/15 v1.4 Provide unlimited unique counter
(HO)
)
Package uniquecounter Info: New unique counter `rerunfilecheck' on input
line 2
85.
)
\Hy@SectionHShift=\skip53
)
Package hyperref Info: Option `breaklinks' set `true' on input line 40.
(c:/texlive/2023/texmf-dist/tex/latex/wrapfig/wrapfig.sty
```

```
\wrapoverhang=\dimen153
\WF@size=\dimen154
\c@WF@wrappedlines=\count286
\WF@box=\box53
\WF@everypar=\toks22
Package: wrapfig 2003/01/31  v 3.6
) (c:/texlive/2023/texmf-dist/tex/latex/dblfloatfix/dblfloatfix.sty
Package: dblfloatfix 2012/12/31 v1.0a (JAW)
(c:/texlive/2023/texmf-dist/tex/latex/base/fixltx2e.sty
Package: fixltx2e 2016/12/29 v2.1a fixes to LaTeX (obsolete)
Applying: [2015/01/01] Old fixltx2e package on input line 46.

Package fixltx2e Warning: fixltx2e is not required with releases after
2015
(fixltx2e)                All fixes are now in the LaTeX kernel.
(fixltx2e)                See the latexrelease package for details.

Already applied: [0000/00/00] Old fixltx2e package on input line 53.
)
\@dblbotnum=\count287
\c@dblbotnumber=\count288
) (c:/texlive/2023/texmf-dist/tex/latex/graphics/graphicx.sty
Package: graphicx 2021/09/16 v1.2d Enhanced LaTeX Graphics (DPC,SPQR)
(c:/texlive/2023/texmf-dist/tex/latex/graphics/graphics.sty
Package: graphics 2022/03/10 v1.4e Standard LaTeX Graphics (DPC,SPQR)
(c:/texlive/2023/texmf-dist/tex/latex/graphics/trig.sty
Package: trig 2021/08/11 v1.11 sin cos tan (DPC)
) (c:/texlive/2023/texmf-dist/tex/latex/graphics-cfg/graphics.cfg
File: graphics.cfg 2016/06/04 v1.11 sample graphics configuration
)
Package graphics Info: Driver file: pdftex.def on input line 107.
(c:/texlive/2023/texmf-dist/tex/latex/graphics-def/pdftex.def
File: pdftex.def 2022/09/22 v1.2b Graphics/color driver for pdftex
))
\Gin@req@height=\dimen155
\Gin@req@width=\dimen156
) (c:/texlive/2023/texmf-dist/tex/latex/tools/enumerate.sty
Package: enumerate 2023/07/04 v3.00 enumerate extensions (DPC)
\@enLab=\toks23
)
\cslhangindent=\skip54
\csllabelwidth=\skip55
(c:/texlive/2023/texmf-dist/tex/latex/tools/calc.sty
Package: calc 2023/07/08 v4.3 Infix arithmetic (KKT,FJ)
\calc@Acount=\count289
\calc@Bcount=\count290
\calc@Adimen=\dimen157
\calc@Bdimen=\dimen158
\calc@Askip=\skip56
\calc@Bskip=\skip57
LaTeX Info: Redefining \setlength on input line 80.
LaTeX Info: Redefining \addtolength on input line 81.
\calc@Ccount=\count291
\calc@Cskip=\skip58
```

```
) (c:/texlive/2023/texmf-dist/tex/latex/tools/longtable.sty
Package: longtable 2023-11-01 v4.19 Multi-page Table package (DPC)
\LTleft=\skip59
\LTright=\skip60
\LTpre=\skip61
\LTpost=\skip62
\LTchunksize=\count292
\LTcapwidth=\dimen159
\LT@head=\box54
\LT@firsthead=\box55
\LT@foot=\box56
\LT@lastfoot=\box57
\LT@gbox=\box58
\LT@cols=\count293
\LT@rows=\count294
\c@LT@tables=\count295
\c@LT@chunks=\count296
\LT@p@ftn=\toks24
) (c:/texlive/2023/texmf-dist/tex/latex/pgf/frontendlayer/tikz.sty
(c:/texlive/
2023/texmf-dist/tex/latex/pgf/basiclayer/pgf.sty (c:/texlive/2023/texmf-
dist/te
x/latex/pgf/utilities/pgfrcs.sty (c:/texlive/2023/texmf-
dist/tex/generic/pgf/ut
ilities/pgfutil-common.tex
\pgfutil@everybye=\toks25
\pgfutil@tempdima=\dimen160
\pgfutil@tempdimb=\dimen161
) (c:/texlive/2023/texmf-dist/tex/generic/pgf/utilities/pgfutil-latex.def
\pgfutil@abb=\box59
) (c:/texlive/2023/texmf-dist/tex/generic/pgf/utilities/pgfrcs.code.tex
(c:/tex
live/2023/texmf-dist/tex/generic/pgf/pgf.revision.tex)
Package: pgfrcs 2023-01-15 v3.1.10 (3.1.10)
))
Package: pgf 2023-01-15 v3.1.10 (3.1.10)
(c:/texlive/2023/texmf-dist/tex/latex/pgf/basiclayer/pgfcore.sty
(c:/texlive/20
23/texmf-dist/tex/latex/pgf/systemlayer/pgfsys.sty
(c:/texlive/2023/texmf-dist/
tex/generic/pgf/systemlayer/pgfsys.code.tex
Package: pgfsys 2023-01-15 v3.1.10 (3.1.10)
(c:/texlive/2023/texmf-dist/tex/generic/pgf/utilities/pgfkeys.code.tex
\pgfkeys@pathtoks=\toks26
\pgfkeys@temptoks=\toks27

(c:/texlive/2023/texmf-
dist/tex/generic/pgf/utilities/pgfkeyslibraryfiltered.co
de.tex
\pgfkeys@tmptoks=\toks28
))
\pgf@x=\dimen162
\pgf@y=\dimen163
\pgf@xa=\dimen164
```

```
\pgf@ya=\dimen165
\pgf@xb=\dimen166
\pgf@yb=\dimen167
\pgf@xc=\dimen168
\pgf@yc=\dimen169
\pgf@xd=\dimen170
\pgf@yd=\dimen171
\w@pgf@writea=\write3
\r@pgf@reada=\read2
\c@pgf@counta=\count297
\c@pgf@countb=\count298
\c@pgf@countc=\count299
\c@pgf@countd=\count300
\t@pgf@toka=\toks29
\t@pgf@tokb=\toks30
\t@pgf@tokc=\toks31
\pgf@sys@id@count=\count301
(c:/texlive/2023/texmf-dist/tex/generic/pgf/systemlayer/pgf.cfg
File: pgf.cfg 2023-01-15 v3.1.10 (3.1.10)
)
Driver file for pgf: pgfsys-pdftex.def
(c:/texlive/2023/texmf-dist/tex/generic/pgf/systemlayer/pgfsys-pdftex.def
File: pgfsys-pdftex.def 2023-01-15 v3.1.10 (3.1.10)
(c:/texlive/2023/texmf-dist/tex/generic/pgf/systemlayer/pgfsys-common-
pdf.def
File: pgfsys-common-pdf.def 2023-01-15 v3.1.10 (3.1.10)
)))
(c:/texlive/2023/texmf-
dist/tex/generic/pgf/systemlayer/pgfsyssoftpath.code.tex
File: pgfsyssoftpath.code.tex 2023-01-15 v3.1.10 (3.1.10)
\pgfsyssoftpath@smallbuffer@items=\count302
\pgfsyssoftpath@bigbuffer@items=\count303
)
(c:/texlive/2023/texmf-
dist/tex/generic/pgf/systemlayer/pgfsysprotocol.code.tex
File: pgfsysprotocol.code.tex 2023-01-15 v3.1.10 (3.1.10)
)) (c:/texlive/2023/texmf-dist/tex/latex/xcolor/xcolor.sty
Package: xcolor 2023/11/15 v3.01 LaTeX color extensions (UK)
(c:/texlive/2023/texmf-dist/tex/latex/graphics-cfg/color.cfg
File: color.cfg 2016/01/02 v1.6 sample color configuration
)
Package xcolor Info: Driver file: pdftex.def on input line 274.
(c:/texlive/2023/texmf-dist/tex/latex/graphics/mathcolor.ltx)
Package xcolor Info: Model `cmy' substituted by `cmy0' on input line
1350.
Package xcolor Info: Model `hsb' substituted by `rgb' on input line 1354.
Package xcolor Info: Model `RGB' extended on input line 1366.
Package xcolor Info: Model `HTML' substituted by `rgb' on input line
1368.
Package xcolor Info: Model `Hsb' substituted by `hsb' on input line 1369.
Package xcolor Info: Model `tHsb' substituted by `hsb' on input line
1370.
Package xcolor Info: Model `HSB' substituted by `hsb' on input line 1371.
```

```
Package xcolor Info: Model `Gray' substituted by `gray' on input line
1372.
Package xcolor Info: Model `wave' substituted by `hsb' on input line
1373.
) (c:/texlive/2023/texmf-dist/tex/generic/pgf/basiclayer/pgfcore.code.tex
Package: pgfcore 2023-01-15 v3.1.10 (3.1.10)
(c:/texlive/2023/texmf-dist/tex/generic/pgf/math/pgfmath.code.tex
(c:/texlive/2
023/texmf-dist/tex/generic/pgf/math/pgfmathutil.code.tex)
(c:/texlive/2023/texm
f-dist/tex/generic/pgf/math/pgfmathparser.code.tex
\pgfmath@dimen=\dimen172
\pgfmath@count=\count304
\pgfmath@box=\box60
\pgfmath@toks=\toks32
\pgfmath@stack@operand=\toks33
\pgfmath@stack@operation=\toks34
) (c:/texlive/2023/texmf-
dist/tex/generic/pgf/math/pgfmathfunctions.code.tex)
(c:/texlive/2023/texmf-
dist/tex/generic/pgf/math/pgfmathfunctions.basic.code.te
x)
(c:/texlive/2023/texmf-
dist/tex/generic/pgf/math/pgfmathfunctions.trigonometric
.code.tex)
(c:/texlive/2023/texmf-
dist/tex/generic/pgf/math/pgfmathfunctions.random.code.t
ex)
(c:/texlive/2023/texmf-
dist/tex/generic/pgf/math/pgfmathfunctions.comparison.co
de.tex)
(c:/texlive/2023/texmf-
dist/tex/generic/pgf/math/pgfmathfunctions.base.code.tex
)
(c:/texlive/2023/texmf-
dist/tex/generic/pgf/math/pgfmathfunctions.round.code.te
x)
(c:/texlive/2023/texmf-
dist/tex/generic/pgf/math/pgfmathfunctions.misc.code.tex
)
(c:/texlive/2023/texmf-
dist/tex/generic/pgf/math/pgfmathfunctions.integerarithm
etics.code.tex) (c:/texlive/2023/texmf-
dist/tex/generic/pgf/math/pgfmathcalc.co
de.tex) (c:/texlive/2023/texmf-
dist/tex/generic/pgf/math/pgfmathfloat.code.tex
\c@pgfmathroundto@lastzeros=\count305
)) (c:/texlive/2023/texmf-dist/tex/generic/pgf/math/pgfint.code.tex)
(c:/texliv
e/2023/texmf-dist/tex/generic/pgf/basiclayer/pgfcorepoints.code.tex
File: pgfcorepoints.code.tex 2023-01-15 v3.1.10 (3.1.10)
\pgf@picminx=\dimen173
\pgf@picmaxx=\dimen174
\pgf@picminy=\dimen175
```

```
\pgf@picmaxy=\dimen176
\pgf@pathminx=\dimen177
\pgf@pathmaxx=\dimen178
\pgf@pathminy=\dimen179
\pgf@pathmaxy=\dimen180
\pgf@xx=\dimen181
\pgf@xy=\dimen182
\pgf@yx=\dimen183
\pgf@yy=\dimen184
\pgf@zx=\dimen185
\pgf@zy=\dimen186
)
(c:/texlive/2023/texmf-
dist/tex/generic/pgf/basiclayer/pgfcorepathconstruct.cod
e.tex
File: pgfcorepathconstruct.code.tex 2023-01-15 v3.1.10 (3.1.10)
\pgf@path@lastx=\dimen187
\pgf@path@lasty=\dimen188
)
(c:/texlive/2023/texmf-
dist/tex/generic/pgf/basiclayer/pgfcorepathusage.code.te
x
File: pgfcorepathusage.code.tex 2023-01-15 v3.1.10 (3.1.10)
\pgf@shorten@end@additional=\dimen189
\pgf@shorten@start@additional=\dimen190
) (c:/texlive/2023/texmf-
dist/tex/generic/pgf/basiclayer/pgfcorescopes.code.tex
File: pgfcorescopes.code.tex 2023-01-15 v3.1.10 (3.1.10)
\pgfpic=\box61
\pgf@hbox=\box62
\pgf@layerbox@main=\box63
\pgf@picture@serial@count=\count306
)
(c:/texlive/2023/texmf-
dist/tex/generic/pgf/basiclayer/pgfcoregraphicstate.code
.tex
File: pgfcoregraphicstate.code.tex 2023-01-15 v3.1.10 (3.1.10)
\pgflinewidth=\dimen191
)
(c:/texlive/2023/texmf-
dist/tex/generic/pgf/basiclayer/pgfcoretransformations.c
ode.tex
File: pgfcoretransformations.code.tex 2023-01-15 v3.1.10 (3.1.10)
\pgf@pt@x=\dimen192
\pgf@pt@y=\dimen193
\pgf@pt@temp=\dimen194
) (c:/texlive/2023/texmf-
dist/tex/generic/pgf/basiclayer/pgfcorequick.code.tex
File: pgfcorequick.code.tex 2023-01-15 v3.1.10 (3.1.10)
) (c:/texlive/2023/texmf-
dist/tex/generic/pgf/basiclayer/pgfcoreobjects.code.te
x
File: pgfcoreobjects.code.tex 2023-01-15 v3.1.10 (3.1.10)
)
```

```
(c:/texlive/2023/texmf-
dist/tex/generic/pgf/basiclayer/pgfcorepathprocessing.co
de.tex
File: pgfcorepathprocessing.code.tex 2023-01-15 v3.1.10 (3.1.10)
) (c:/texlive/2023/texmf-
dist/tex/generic/pgf/basiclayer/pgfcorearrows.code.tex
File: pgfcorearrows.code.tex 2023-01-15 v3.1.10 (3.1.10)
\pgfarrowsep=\dimen195
) (c:/texlive/2023/texmf-
dist/tex/generic/pgf/basiclayer/pgfcoreshade.code.tex
File: pgfcoreshade.code.tex 2023-01-15 v3.1.10 (3.1.10)
\pgf@max=\dimen196
\pgf@sys@shading@range@num=\count307
\pgf@shadingcount=\count308
) (c:/texlive/2023/texmf-
dist/tex/generic/pgf/basiclayer/pgfcoreimage.code.tex
File: pgfcoreimage.code.tex 2023-01-15 v3.1.10 (3.1.10)
)
(c:/texlive/2023/texmf-
dist/tex/generic/pgf/basiclayer/pgfcoreexternal.code.tex
File: pgfcoreexternal.code.tex 2023-01-15 v3.1.10 (3.1.10)
\pgfexternal@startupbox=\box64
) (c:/texlive/2023/texmf-
dist/tex/generic/pgf/basiclayer/pgfcorelayers.code.tex
File: pgfcorelayers.code.tex 2023-01-15 v3.1.10 (3.1.10)
)
(c:/texlive/2023/texmf-
dist/tex/generic/pgf/basiclayer/pgfcoretransparency.code
.tex
File: pgfcoretransparency.code.tex 2023-01-15 v3.1.10 (3.1.10)
)
(c:/texlive/2023/texmf-
dist/tex/generic/pgf/basiclayer/pgfcorepatterns.code.tex
File: pgfcorepatterns.code.tex 2023-01-15 v3.1.10 (3.1.10)
) (c:/texlive/2023/texmf-
dist/tex/generic/pgf/basiclayer/pgfcorerdf.code.tex
File: pgfcorerdf.code.tex 2023-01-15 v3.1.10 (3.1.10)
))) (c:/texlive/2023/texmf-
dist/tex/generic/pgf/modules/pgfmoduleshapes.code.te
x
File: pgfmoduleshapes.code.tex 2023-01-15 v3.1.10 (3.1.10)
\pgfnodeparttextbox=\box65
) (c:/texlive/2023/texmf-
dist/tex/generic/pgf/modules/pgfmoduleplot.code.tex
File: pgfmoduleplot.code.tex 2023-01-15 v3.1.10 (3.1.10)
)
(c:/texlive/2023/texmf-dist/tex/latex/pgf/compatibility/pgfcomp-version-
0-65.st
y
Package: pgfcomp-version-0-65 2023-01-15 v3.1.10 (3.1.10)
\pgf@nodesepstart=\dimen197
\pgf@nodesepend=\dimen198
)
```

```
(c:/texlive/2023/texmf-dist/tex/latex/pgf/compatibility/pgfcomp-version-
1-18.st
y
Package: pgfcomp-version-1-18 2023-01-15 v3.1.10 (3.1.10)
)) (c:/texlive/2023/texmf-dist/tex/latex/pgf/utilities/pgffor.sty
(c:/texlive/2
023/texmf-dist/tex/latex/pgf/utilities/pgfkeys.sty
(c:/texlive/2023/texmf-dist/
tex/generic/pgf/utilities/pgfkeys.code.tex)) (c:/texlive/2023/texmf-
dist/tex/la
tex/pgf/math/pgfmath.sty (c:/texlive/2023/texmf-
dist/tex/generic/pgf/math/pgfma
th.code.tex)) (c:/texlive/2023/texmf-
dist/tex/generic/pgf/utilities/pgffor.code
.tex
Package: pgffor 2023-01-15 v3.1.10 (3.1.10)
\pgffor@iter=\dimen199
\pgffor@skip=\dimen256
\pgffor@stack=\toks35
\pgffor@toks=\toks36
)) (c:/texlive/2023/texmf-
dist/tex/generic/pgf/frontendlayer/tikz/tikz.code.tex
Package: tikz 2023-01-15 v3.1.10 (3.1.10)

(c:/texlive/2023/texmf-
dist/tex/generic/pgf/libraries/pgflibraryplothandlers.co
de.tex
File: pgflibraryplothandlers.code.tex 2023-01-15 v3.1.10 (3.1.10)
\pgf@plot@mark@count=\count309
\pgfplotmarksize=\dimen257
)
\tikz@lastx=\dimen258
\tikz@lasty=\dimen259
\tikz@lastxsaved=\dimen260
\tikz@lastysaved=\dimen261
\tikz@lastmovetox=\dimen262
\tikz@lastmovetoy=\dimen263
\tikzleveldistance=\dimen264
\tikzsiblingdistance=\dimen265
\tikz@figbox=\box66
\tikz@figbox@bg=\box67
\tikz@tempbox=\box68
\tikz@tempbox@bg=\box69
\tikztreelevel=\count310
\tikznumberofchildren=\count311
\tikznumberofcurrentchild=\count312
\tikz@fig@count=\count313
(c:/texlive/2023/texmf-
dist/tex/generic/pgf/modules/pgfmodulematrix.code.tex
File: pgfmodulematrix.code.tex 2023-01-15 v3.1.10 (3.1.10)
\pgfmatrixcurrentrow=\count314
\pgfmatrixcurrentcolumn=\count315
\pgf@matrix@numberofcolumns=\count316
)
```

```
\tikz@expandcount=\count317

(c:/texlive/2023/texmf-
dist/tex/generic/pgf/frontendlayer/tikz/libraries/tikzli
brarytopaths.code.tex
File: tikzlibrarytopaths.code.tex 2023-01-15 v3.1.10 (3.1.10)
))) (c:/texlive/2023/texmf-dist/tex/latex/csvsimple/csvsimple.sty
Package: csvsimple 2024/01/19 v2.6.0 LaTeX CSV file processing
) (c:/texlive/2023/texmf-dist/tex/latex/csvsimple/csvsimple-legacy.sty
Package: csvsimple-legacy 2024/01/19 version 2.6.0 LaTeX2e CSV file
processing
(c:/texlive/2023/texmf-dist/tex/latex/tools/shellesc.sty
Package: shellesc 2023/07/08 v1.0d unified shell escape interface for
LaTeX
Package shellesc Info: Restricted shell escape enabled on input line 77.
)
\csv@file=\read3
\c@csvinputline=\count318
\c@csvrow=\count319
\c@csvcol=\count320
\csv@out=\write4
) (c:/texlive/2023/texmf-dist/tex/latex/booktabs/booktabs.sty
Package: booktabs 2020/01/12 v1.61803398 Publication quality tables
\heavyrulewidth=\dimen266
\lightrulewidth=\dimen267
\cmidrulewidth=\dimen268
\belowrulesep=\dimen269
\belowbottomsep=\dimen270
\aboverulesep=\dimen271
\abovetopsep=\dimen272
\cmidrulesep=\dimen273
\cmidrulekern=\dimen274
\defaultaddspace=\dimen275
\@cmidla=\count321
\@cmidlb=\count322
\@aboverulesep=\dimen276
\@belowrulesep=\dimen277
\@thisruleclass=\count323
\@lastruleclass=\count324
\@thisrulewidth=\dimen278
) (c:/texlive/2023/texmf-dist/tex/latex/tools/tabularx.sty
Package: tabularx 2023/07/08 v2.11c `tabularx' package (DPC)
(c:/texlive/2023/texmf-dist/tex/latex/tools/array.sty
Package: array 2023/10/16 v2.5g Tabular extension package (FMi)
\col@sep=\dimen279
\ar@mcellbox=\box70
\extrarowheight=\dimen280
\NC@list=\toks37
\extratabsurround=\skip63
\backup@length=\skip64
\ar@cellbox=\box71
)
\TX@col@width=\dimen281
\TX@old@table=\dimen282
```

```
\TX@old@col=\dimen283
\TX@target=\dimen284
\TX@delta=\dimen285
\TX@cols=\count325
\TX@ftn=\toks38
) (c:/texlive/2023/texmf-dist/tex/latex/ifsym/ifsym.sty
Package: ifsym 2000/04/18 I.Kloeckl
\c@ifsymcnt=\count326
) (c:/texlive/2023/texmf-dist/tex/latex/multirow/multirow.sty
Package: multirow 2021/03/15 v2.8 Span multiple rows of a table
\multirow@colwidth=\skip65
\multirow@cntb=\count327
\multirow@dima=\skip66
\bigstrutjot=\dimen286
) (c:/texlive/2023/texmf-dist/tex/latex/preprint/authblk.sty
Package: authblk 2001/02/27 1.3 (PWD)
\affilsep=\skip67
\@affilsep=\skip68
\c@Maxaffil=\count328
\c@authors=\count329
\c@affil=\count330
) (c:/texlive/2023/texmf-dist/tex/latex/sttools/flushend.sty
Package: flushend 2021/10/04 v4.0 Balancing columns in twocolumn mode
\flushend@@lastskip@a=\skip69
\flushend@@lastskip@b=\skip70
\flushend@@lastnode=\count331
\var@@loop@iter=\count332
\var@@temp@spread=\dimen287
\var@@temp@a=\dimen288
\var@@temp@loop=\dimen289
\flushend@@page@rule=\dimen290
\flushend@@varbox@lastpage=\box72
\flushend@@varbox@a=\box73
\flushend@@varbox@c=\box74
\flushend@@tempbox@a=\box75
\flushend@@tempbox@c=\box76
\flushend@@floatbox=\box77
\@viper=\box78
\hold@viper=\box79
\atColsBreak=\toks39
\atColsEnd=\toks40
\oldbreak@skip=\dimen291
) (c:/texlive/2023/texmf-dist/tex/latex/pdfpages/pdfpages.sty
Package: pdfpages 2024/01/21 v0.5y Insert pages of external PDF documents
(AM)
(c:/texlive/2023/texmf-dist/tex/latex/eso-pic/eso-pic.sty
Package: eso-pic 2023/05/03 v3.0c eso-pic (RN)
\ESO@tempdima=\dimen292
\ESO@tempdimb=\dimen293
)
\AM@pagewidth=\dimen294
\AM@pageheight=\dimen295
\AM@fboxrule=\dimen296
(c:/texlive/2023/texmf-dist/tex/latex/pdfpages/pppdftex.def
```

```
File: pppdftex.def 2024/01/21 v0.5y Pdfpages driver for pdfTeX (AM)
)
\pdfpages@includegraphics@status=\count333
\AM@pagebox=\box80
\AM@global@opts=\toks41
\AM@pagecnt=\count334
\AM@toc@title=\toks42
\AM@lof@heading=\toks43
\c@AM@survey=\count335
\AM@templatesizebox=\box81
) (c:/texlive/2023/texmf-dist/tex/latex/caption/caption.sty
Package: caption 2023/08/05 v3.6o Customizing captions (AR)
(c:/texlive/2023/texmf-dist/tex/latex/caption/caption3.sty
Package: caption3 2023/07/31 v2.4d caption3 kernel (AR)
\caption@tempdima=\dimen297
\captionmargin=\dimen298
\caption@leftmargin=\dimen299
\caption@rightmargin=\dimen300
\caption@width=\dimen301
\caption@indent=\dimen302
\caption@parindent=\dimen303
\caption@hangindent=\dimen304
Package caption Info: Standard document class detected.
)
\c@caption@flags=\count336
\c@continuedfloat=\count337
Package caption Info: float package is loaded.
Package caption Info: hyperref package is loaded.
Package caption Info: longtable package is loaded.
(c:/texlive/2023/texmf-dist/tex/latex/caption/ltcaption.sty
Package: ltcaption 2021/01/08 v1.4c longtable captions (AR)
)
Package caption Info: wrapfig package is loaded.
) (c:/texlive/2023/texmf-dist/tex/latex/amsfonts/amssymb.sty
Package: amssymb 2013/01/14 v3.01 AMS font symbols
(c:/texlive/2023/texmf-dist/tex/latex/amsfonts/amsfonts.sty
Package: amsfonts 2013/01/14 v3.01 Basic AMSFonts support
\@emptytoks=\toks44
\symAMSa=\mathgroup4
\symAMSb=\mathgroup5
LaTeX Font Info:    Redeclaring math symbol \hbar on input line 98.
LaTeX Font Info:    Overwriting math alphabet `\mathfrak' in version
`bold'
(Font)                  U/euf/m/n --> U/euf/b/n on input line 106.
)) (c:/texlive/2023/texmf-dist/tex/latex/amsmath/amsmath.sty
Package: amsmath 2023/05/13 v2.17o AMS math features
\@mathmargin=\skip71
For additional information on amsmath, use the `?' option.
(c:/texlive/2023/texmf-dist/tex/latex/amsmath/amstext.sty
Package: amstext 2021/08/26 v2.01 AMS text
(c:/texlive/2023/texmf-dist/tex/latex/amsmath/amsgen.sty
File: amsgen.sty 1999/11/30 v2.0 generic functions
\@emptytoks=\toks45
\ex@=\dimen305
```

```
)) (c:/texlive/2023/texmf-dist/tex/latex/amsmath/amsbsy.sty
Package: amsbsy 1999/11/29 v1.2d Bold Symbols
\pmbraise@=\dimen306
) (c:/texlive/2023/texmf-dist/tex/latex/amsmath/amsopn.sty
Package: amsopn 2022/04/08 v2.04 operator names
)
\inf@bad=\count338
LaTeX Info: Redefining \frac on input line 234.
\uproot@=\count339
\leftroot@=\count340
LaTeX Info: Redefining \overline on input line 399.
LaTeX Info: Redefining \colon on input line 410.
\classnum@=\count341
\DOTSCASE@=\count342
LaTeX Info: Redefining \ldots on input line 496.
LaTeX Info: Redefining \dots on input line 499.
LaTeX Info: Redefining \cdots on input line 620.
\Mathstrutbox@=\box82
\strutbox@=\box83
LaTeX Info: Redefining \big on input line 722.
LaTeX Info: Redefining \Big on input line 723.
LaTeX Info: Redefining \bigg on input line 724.
LaTeX Info: Redefining \Bigg on input line 725.
\big@size=\dimen307
LaTeX Font Info:    Redeclaring font encoding OML on input line 743.
LaTeX Font Info:    Redeclaring font encoding OMS on input line 744.
\macc@depth=\count343
LaTeX Info: Redefining \bmod on input line 905.
LaTeX Info: Redefining \pmod on input line 910.
LaTeX Info: Redefining \smash on input line 940.
LaTeX Info: Redefining \relbar on input line 970.
LaTeX Info: Redefining \Relbar on input line 971.
\c@MaxMatrixCols=\count344
\dotsspace@=\muskip17
\c@parentequation=\count345
\dspbrk@lvl=\count346
\tag@help=\toks46
\row@=\count347
\column@=\count348
\maxfields@=\count349
\andhelp@=\toks47
\eqnshift@=\dimen308
\alignsep@=\dimen309
\tagshift@=\dimen310
\tagwidth@=\dimen311
\totwidth@=\dimen312
\lineht@=\dimen313
\@envbody=\toks48
\multlinegap=\skip72
\multlinetaggap=\skip73
\mathdisplay@stack=\toks49
LaTeX Info: Redefining \[ on input line 2953.
LaTeX Info: Redefining \] on input line 2954.
) (c:/texlive/2023/texmf-dist/tex/latex/base/inputenc.sty
```

```
Package: inputenc 2021/02/14 v1.3d Input encoding file
\inpenc@prehook=\toks50
\inpenc@posthook=\toks51
)
Package hyperref Info: Option `breaklinks' set `true' on input line 295.
Package hyperref Info: Option `colorlinks' set `true' on input line 295.
(c:/texlive/2023/texmf-dist/tex/latex/tools/bm.sty
Package: bm 2023/07/08 v1.2f Bold Symbol Support (DPC/FMi)
\symboldoperators=\mathgroup6
\symboldletters=\mathgroup7
\symboldsymbols=\mathgroup8
Package bm Info: No bold for \OMX/cmex/m/n, using \pmb.
Package bm Info: No bold for \U/msa/m/n, using \pmb.
Package bm Info: No bold for \U/msb/m/n, using \pmb.
LaTeX Font Info:    Redeclaring math alphabet \mathbf on input line 149.
) (c:/texlive/2023/texmf-dist/tex/latex/fancyhdr/fancyhdr.sty
Package: fancyhdr 2022/11/09 v4.1 Extensive control of page headers and
footers

\f@nch@headwidth=\skip74
\f@nch@O@elh=\skip75
\f@nch@O@erh=\skip76
\f@nch@O@olh=\skip77
\f@nch@O@orh=\skip78
\f@nch@O@elf=\skip79
\f@nch@O@erf=\skip80
\f@nch@O@olf=\skip81
\f@nch@O@orf=\skip82
) (c:/texlive/2023/texmf-dist/tex/latex/mdframed/mdframed.sty
Package: mdframed 2013/07/01 1.9b: mdframed
(c:/texlive/2023/texmf-dist/tex/latex/l3packages/xparse/xparse.sty
(c:/texlive/
2023/texmf-dist/tex/latex/l3kernel/expl3.sty
Package: expl3 2024-02-20 L3 programming layer (loader)
(c:/texlive/2023/texmf-dist/tex/latex/l3backend/l3backend-pdftex.def
File: l3backend-pdftex.def 2024-02-20 L3 backend support: PDF output
(pdfTeX)
\l__color_backend_stack_int=\count350
\l__pdf_internal_box=\box84
))
Package: xparse 2024-02-18 L3 Experimental document command parser
) (c:/texlive/2023/texmf-dist/tex/latex/zref/zref-abspage.sty
Package: zref-abspage 2023-09-14 v2.35 Module abspage for zref (HO)
(c:/texlive/2023/texmf-dist/tex/latex/zref/zref-base.sty
Package: zref-base 2023-09-14 v2.35 Module base for zref (HO)
(c:/texlive/2023/texmf-dist/tex/generic/etexcmds/etexcmds.sty
Package: etexcmds 2019/12/15 v1.7 Avoid name clashes with e-TeX commands
(HO)
)
Package zref Info: New property list: main on input line 767.
Package zref Info: New property: default on input line 768.
Package zref Info: New property: page on input line 769.
)
\c@abspage=\count351
```

```
Package zref Info: New property: abspage on input line 67.
) (c:/texlive/2023/texmf-dist/tex/latex/needspace/needspace.sty
Package: needspace 2010/09/12 v1.3d reserve vertical space
)
\mdf@templength=\skip83
\c@mdf@globalstyle@cnt=\count352
\mdf@skipabove@length=\skip84
\mdf@skipbelow@length=\skip85
\mdf@leftmargin@length=\skip86
\mdf@rightmargin@length=\skip87
\mdf@innerleftmargin@length=\skip88
\mdf@innerrightmargin@length=\skip89
\mdf@innertopmargin@length=\skip90
\mdf@innerbottommargin@length=\skip91
\mdf@splittopskip@length=\skip92
\mdf@splitbottomskip@length=\skip93
\mdf@outermargin@length=\skip94
\mdf@innermargin@length=\skip95
\mdf@linewidth@length=\skip96
\mdf@innerlinewidth@length=\skip97
\mdf@middlelinewidth@length=\skip98
\mdf@outerlinewidth@length=\skip99
\mdf@roundcorner@length=\skip100
\mdf@footenotedistance@length=\skip101
\mdf@userdefinedwidth@length=\skip102
\mdf@needspace@length=\skip103
\mdf@frametitleaboveskip@length=\skip104
\mdf@frametitlebelowskip@length=\skip105
\mdf@frametitlerulewidth@length=\skip106
\mdf@frametitleleftmargin@length=\skip107
\mdf@frametitlerightmargin@length=\skip108
\mdf@shadowsize@length=\skip109
\mdf@extratopheight@length=\skip110
\mdf@subtitleabovelinewidth@length=\skip111
\mdf@subtitlebelowlinewidth@length=\skip112
\mdf@subtitleaboveskip@length=\skip113
\mdf@subtitlebelowskip@length=\skip114
\mdf@subtitleinneraboveskip@length=\skip115
\mdf@subtitleinnerbelowskip@length=\skip116
\mdf@subsubtitleabovelinewidth@length=\skip117
\mdf@subsubtitlebelowlinewidth@length=\skip118
\mdf@subsubtitleaboveskip@length=\skip119
\mdf@subsubtitlebelowskip@length=\skip120
\mdf@subsubtitleinneraboveskip@length=\skip121
\mdf@subsubtitleinnerbelowskip@length=\skip122
(c:/texlive/2023/texmf-dist/tex/latex/mdframed/md-frame-0.mdf
File: md-frame-0.mdf 2013/07/01\ 1.9b: md-frame-0
)
\mdf@frametitlebox=\box85
\mdf@footnotebox=\box86
\mdf@splitbox@one=\box87
\mdf@splitbox@two=\box88
\mdf@splitbox@save=\box89
\mdfsplitboxwidth=\skip123
```

```
\mdfsplitboxtotalwidth=\skip124
\mdfsplitboxheight=\skip125
\mdfsplitboxdepth=\skip126
\mdfsplitboxtotalheight=\skip127
\mdfframetitleboxwidth=\skip128
\mdfframetitleboxtotalwidth=\skip129
\mdfframetitleboxheight=\skip130
\mdfframetitleboxdepth=\skip131
\mdfframetitleboxtotalheight=\skip132
\mdffootnoteboxwidth=\skip133
\mdffootnoteboxtotalwidth=\skip134
\mdffootnoteboxheight=\skip135
\mdffootnoteboxdepth=\skip136
\mdffootnoteboxtotalheight=\skip137
\mdftotallinewidth=\skip138
\mdfboundingboxwidth=\skip139
\mdfboundingboxtotalwidth=\skip140
\mdfboundingboxheight=\skip141
\mdfboundingboxdepth=\skip142
\mdfboundingboxtotalheight=\skip143
\mdf@freevspace@length=\skip144
\mdf@horizontalwidthofbox@length=\skip145
\mdf@verticalmarginwhole@length=\skip146
\mdf@horizontalspaceofbox=\skip147
\mdfsubtitleheight=\skip148
\mdfsubsubtitleheight=\skip149
\c@mdfcountframes=\count353

****** mdframed patching \endmdf@trivlist

****** -- success******

\mdf@envdepth=\count354
\c@mdf@env@i=\count355
\c@mdf@env@ii=\count356
\c@mdf@zref@counter=\count357
Package zref Info: New property: mdf@pagevalue on input line 895.
) (c:/texlive/2023/texmf-dist/tex/latex/titlesec/titlesec.sty
Package: titlesec 2023/10/27 v2.16 Sectioning titles
\ttl@box=\box90
\beforetitleunit=\skip150
\aftertitleunit=\skip151
\ttl@plus=\dimen314
\ttl@minus=\dimen315
\ttl@toksa=\toks52
\titlewidth=\dimen316
\titlewidthlast=\dimen317
\titlewidthfirst=\dimen318
) (c:/texlive/2023/texmf-dist/tex/latex/amscls/amsthm.sty
Package: amsthm 2020/05/29 v2.20.6
\thm@style=\toks53
\thm@bodyfont=\toks54
\thm@headfont=\toks55
\thm@notefont=\toks56
```

```
\thm@headpunct=\toks57
\thm@preskip=\skip152
\thm@postskip=\skip153
\thm@headsep=\skip154
\dth@everypar=\toks58
) (c:/texlive/2023/texmf-dist/tex/latex/enumitem/enumitem.sty
Package: enumitem 2019/06/20 v3.9 Customized lists
\labelindent=\skip155
\enit@outerparindent=\dimen319
\enit@toks=\toks59
\enit@inbox=\box91
\enit@count@id=\count358
\enitdp@description=\count359
) (c:/texlive/2023/texmf-dist/tex/latex/titling/titling.sty
Package: titling 2009/09/04 v2.1d maketitle typesetting
\thanksmarkwidth=\skip156
\thanksmargin=\skip157
\droptitle=\skip158
)
LaTeX Font Info:    Trying to load font information for OT1+bch on input
line 4
79.
(c:/texlive/2023/texmf-dist/tex/latex/psnfss/ot1bch.fd
File: ot1bch.fd 2004/10/18 font definitions for OT1/bch.
) (./manuscript_2024-05-10.aux)
\openout1 = `manuscript_2024-05-10.aux'.

LaTeX Font Info:    Checking defaults for OML/cmm/m/it on input line 479.
LaTeX Font Info:    ... okay on input line 479.
LaTeX Font Info:    Checking defaults for OMS/cmsy/m/n on input line 479.
LaTeX Font Info:    ... okay on input line 479.
LaTeX Font Info:    Checking defaults for OT1/cmr/m/n on input line 479.
LaTeX Font Info:    ... okay on input line 479.
LaTeX Font Info:    Checking defaults for T1/cmr/m/n on input line 479.
LaTeX Font Info:    ... okay on input line 479.
LaTeX Font Info:    Checking defaults for TS1/cmr/m/n on input line 479.
LaTeX Font Info:    ... okay on input line 479.
LaTeX Font Info:    Checking defaults for OMX/cmex/m/n on input line 479.
LaTeX Font Info:    ... okay on input line 479.
LaTeX Font Info:    Checking defaults for U/cmr/m/n on input line 479.
LaTeX Font Info:    ... okay on input line 479.
LaTeX Font Info:    Checking defaults for PD1/pdf/m/n on input line 479.
LaTeX Font Info:    ... okay on input line 479.
LaTeX Font Info:    Checking defaults for PU/pdf/m/n on input line 479.
LaTeX Font Info:    ... okay on input line 479.
*geometry* driver: auto-detecting
*geometry* detected driver: pdftex
*geometry* verbose mode - [ preamble ] result:
* driver: pdftex
* paper: <default>
* layout: <same size as paper>
* layoutoffset:(h,v)=(0.0pt,0.0pt)
* modes: includefoot
* h-part:(L,W,R)=(57.81621pt, 498.66258pt, 57.81621pt)
```

```
* v-part:(T,H,B)=(57.81621pt, 715.47256pt, 21.68121pt)
* \paperwidth=614.295pt
* \paperheight=794.96999pt
* \textwidth=498.66258pt
* \textheight=685.47256pt
* \oddsidemargin=-14.45378pt
* \evensidemargin=-14.45378pt
* \topmargin=-51.45378pt
* \headheight=12.0pt
* \headsep=25.0pt
* \topskip=10.0pt
* \footskip=30.0pt
* \marginparwidth=65.0pt
* \marginparsep=11.0pt
* \columnsep=10.0pt
* \skip\footins=9.0pt plus 4.0pt minus 2.0pt
* \hoffset=0.0pt
* \voffset=0.0pt
* \mag=1000
* \@twocolumnfalse
* \@twosidefalse
* \@mparswitchfalse
* \@reversemarginfalse
* (1in=72.27pt=25.4mm, 1cm=28.453pt)

Package hyperref Info: Link coloring ON on input line 479.
(./manuscript_2024-05-10.out) (./manuscript_2024-05-10.out)
\@outlinefile=\write5
\openout5 = `manuscript_2024-05-10.out'.

(c:/texlive/2023/texmf-dist/tex/context/base/mkii/supp-pdf.mkii
[Loading MPS to PDF converter (version 2006.09.02).]
\scratchcounter=\count360
\scratchdimen=\dimen320
\scratchbox=\box92
\nofMPsegments=\count361
\nofMParguments=\count362
\everyMPshowfont=\toks60
\MPscratchCnt=\count363
\MPscratchDim=\dimen321
\MPnumerator=\count364
\makeMPintoPDFobject=\count365
\everyMPtoPDFconversion=\toks61
) (c:/texlive/2023/texmf-dist/tex/latex/epstopdf-pkg/epstopdf-base.sty
Package: epstopdf-base 2020-01-24 v2.11 Base part for package epstopdf
Package epstopdf-base Info: Redefining graphics rule for `.eps' on input
line 4
85.
(c:/texlive/2023/texmf-dist/tex/latex/latexconfig/epstopdf-sys.cfg
File: epstopdf-sys.cfg 2010/07/13 v1.3 Configuration of (r)epstopdf for
TeX Liv
e
)) (c:/texlive/2023/texmf-dist/tex/latex/pdflscape/pdflscape.sty
Package: pdflscape 2022-10-27 v0.13 Display of landscape pages in PDF
```

```
(c:/texlive/2023/texmf-dist/tex/latex/pdflscape/pdflscape-nometadata.sty
Package: pdflscape-nometadata 2022-10-28 v0.13 Display of landscape pages
in PD
F (HO)
(c:/texlive/2023/texmf-dist/tex/latex/graphics/lscape.sty
Package: lscape 2020/05/28 v3.02 Landscape Pages (DPC)
)
Package pdflscape Info: Auto-detected driver: pdftex on input line 81.
))
Package caption Info: Begin \AtBeginDocument code.
Package caption Info: End \AtBeginDocument code.
LaTeX Font Info:    Trying to load font information for U+msa on input
line 503
.
(c:/texlive/2023/texmf-dist/tex/latex/amsfonts/umsa.fd
File: umsa.fd 2013/01/14 v3.01 AMS symbols A
)
LaTeX Font Info:    Trying to load font information for U+msb on input
line 503
.
(c:/texlive/2023/texmf-dist/tex/latex/amsfonts/umsb.fd
File: umsb.fd 2013/01/14 v3.01 AMS symbols B
)
LaTeX Font Info:    Trying to load font information for U+ifsym on input
line 5
03.
(c:/texlive/2023/texmf-dist/tex/latex/ifsym/uifsym.fd)
Package mdframed Info: mdframed detected package amsthm
 changed the theorem header of amsthm
(mdframed)              on input line 503.
Package mdframed Info: mdframed inside a box
 mdframed uses option nobreak mdframed on input line 503.
LaTeX Font Info:    Trying to load font information for OT1+phv on input
line 5
07.
(c:/texlive/2023/texmf-dist/tex/latex/psnfss/ot1phv.fd
File: ot1phv.fd 2020/03/25 scalable font definitions for OT1/phv.
)
Underfull \hbox (badness 1019) in paragraph at lines 528--539
\OT1/bch/m/n/10 and re-trieval [12]; none fo-cus on sim-pli-fy-ing user
 []


LaTeX Warning: File `fig/pdf/fig1.pdf' not found on input line 550.


! Package pdftex.def Error: File `fig/pdf/fig1.pdf' not found: using
draft sett
ing.

See the pdftex.def package documentation for explanation.
Type  H <return>  for immediate help.
 ...
```

```
l.550 ...ntering\includegraphics{fig/pdf/fig1.pdf}
                                                  \caption{\textbf{Figure
Try typing  <return>  to proceed.
If that doesn't work, type  X <return>  to quit.

LaTeX Font Info:    Trying to load font information for OMS+cmtt on input
line
561.
LaTeX Font Info:    No file OMScmtt.fd. on input line 561.

LaTeX Font Warning: Font shape `OMS/cmtt/m/it' undefined
(Font)              using `OMS/cmsy/m/n' instead
(Font)              for symbol `textbraceleft' on input line 561.

LaTeX Font Info:    Trying to load font information for TS1+bch on input
line 5
72.
(c:/texlive/2023/texmf-dist/tex/latex/psnfss/ts1bch.fd
File: ts1bch.fd 2004/10/18 font definitions for TS1/bch.
) [1{c:/texlive/2023/texmf-
var/fonts/map/pdftex/updmap/pdftex.map}{c:/texlive/2
023/texmf-dist/fonts/enc/dvips/base/8r.enc}


]

Package hyperref Warning: The anchor of a bookmark and its parent's must
not
(hyperref)                be the same. Added a new anchor on input line
579.

LaTeX Font Info:    Trying to load font information for OML+cmtt on input
line
596.
LaTeX Font Info:    No file OMLcmtt.fd. on input line 596.

LaTeX Font Warning: Font shape `OML/cmtt/m/n' undefined
(Font)              using `OML/cmm/m/it' instead
(Font)              for symbol `textless' on input line 596.


LaTeX Warning: File `fig/pdf/fig2.pdf' not found on input line 614.


! Package pdftex.def Error: File `fig/pdf/fig2.pdf' not found: using
draft sett
ing.

See the pdftex.def package documentation for explanation.
Type  H <return>  for immediate help.
 ...

l.614 ...ntering\includegraphics{fig/pdf/fig2.pdf}
                                                  \caption{\textbf{Figure
```

```
Try typing  <return>  to proceed.
If that doesn't work, type  X <return>  to quit.


Underfull \hbox (badness 1448) in paragraph at lines 633--645
\OT1/bch/m/n/10 struc-ture in-cludes project-level at-tributes, a sam-ple
 []


Underfull \hbox (badness 2368) in paragraph at lines 649--675
[]\OT1/bch/m/n/10 To im-prove bi-o-log-i-cal meta-data dis-cov-ery, PE-
Phub
 []

[2]

LaTeX Warning: File `fig/pdf/fig3.pdf' not found on input line 715.


! Package pdftex.def Error: File `fig/pdf/fig3.pdf' not found: using
draft sett
ing.

See the pdftex.def package documentation for explanation.
Type  H <return>  for immediate help.
 ...

l.715 ...ntering\includegraphics{fig/pdf/fig3.pdf}
                                                  \caption{\textbf{Figure
Try typing  <return>  to proceed.
If that doesn't work, type  X <return>  to quit.


Underfull \hbox (badness 1097) in paragraph at lines 749--762
\OT1/bch/m/n/10 find-abil-ity, and in-ter-op-er-abil-ity of bi-o-log-i-
cal sam-
ple
 []


LaTeX Warning: File `fig/pdf/fig4.pdf' not found on input line 765.


! Package pdftex.def Error: File `fig/pdf/fig4.pdf' not found: using
draft sett
ing.

See the pdftex.def package documentation for explanation.
Type  H <return>  for immediate help.
 ...

l.765 \includegraphics{fig/pdf/fig4.pdf}

Try typing  <return>  to proceed.
```

```
If that doesn't work, type  X <return>  to quit.

[3]

Package hyperref Warning: Difference (2) between bookmark levels is
greater
(hyperref)                than one, level fixed on input line 808.


Package hyperref Warning: The anchor of a bookmark and its parent's must
not
(hyperref)                be the same. Added a new anchor on input line
808.


Underfull \hbox (badness 4886) in paragraph at lines 810--819
\OT1/bch/m/n/10 work op-ti-mized for speed and high-performance.
 []


Underfull \hbox (badness 1107) in paragraph at lines 810--819
\OT1/bch/m/n/10 We chose FastAPI for its au-to-matic data val-i-da-tion
 []

[4]
Underfull \hbox (badness 2111) in paragraph at lines 892--904
\OT1/bch/m/n/10 rep-re-sen-ta-tion of the sam-ple met-data to cre-ate a
 []


Underfull \hbox (badness 1389) in paragraph at lines 949--954
\OT1/bch/m/n/10 Cohn SL, Gross-man R. Data Com-mons to Sup-port
 []


Underfull \hbox (badness 3536) in paragraph at lines 949--954
\OT1/bch/m/n/10 Pe-di-atric Can-cer Re-search. Amer-i-can So-ci-ety of
 []


Overfull \hbox (23.80174pt too wide) in paragraph at lines 960--964
\OT1/bch/m/n/10 Science. 2022;11:giac053.
doi:[][]10.1093/gigascience/giac053[]
[].
 []


Underfull \hbox (badness 1603) in paragraph at lines 971--976
[]\OT1/bch/m/n/10 5. Wilkin-son MD, Du-mon-tier M, Aal-bers-berg IjJ,
 []


Underfull \hbox (badness 1552) in paragraph at lines 971--976
\OT1/bch/m/n/10 Ap-ple-ton G, Ax-ton M, Baak A, et al. The FAIR
```

```
 []


Underfull \hbox (badness 3291) in paragraph at lines 971--976
\OT1/bch/m/n/10 Guid-ing Prin-ci-ples for sci-en-tific data man-age-ment
 []


Underfull \hbox (badness 1194) in paragraph at lines 994--999
\OT1/bch/m/n/10 (BD2K) ini-tia-tive. Jour-nal of the Amer-i-can Med-i-
 []

[5]
Underfull \hbox (badness 1281) in paragraph at lines 1000--1004
[]\OT1/bch/m/n/10 10. Leipzig J, N[]ust D, Hoyt CT, Ram K, Green-
 []


Underfull \hbox (badness 4713) in paragraph at lines 1000--1004
\OT1/bch/m/n/10 pu-ta-tional re-search. Pat-terns. 2021;2:100322.
 []


Underfull \hbox (badness 1014) in paragraph at lines 1009--1014
\OT1/bch/m/n/10 sys-tem for in-te-grated ge-nomic datasets. Database.
 []


Underfull \hbox (badness 7869) in paragraph at lines 1015--1020
\OT1/bch/m/n/10 MJ. GeMI: In-ter-ac-tive in-ter-face for transformer-
 []


Underfull \hbox (badness 3884) in paragraph at lines 1015--1020
\OT1/bch/m/n/10 based Ge-nomic Meta-data In-te-gra-tion. Database.
 []


Underfull \hbox (badness 2096) in paragraph at lines 1021--1026
[]\OT1/bch/m/n/10 14. Masseroli M, Pinoli P, Venco F, Kaitoua A,
 []


Underfull \hbox (badness 2707) in paragraph at lines 1021--1026
\OT1/bch/m/n/10 guage: A novel ap-proach to large-scale ge-nomic
 []


Underfull \hbox (badness 3838) in paragraph at lines 1027--1032
\OT1/bch/m/n/10 tween the Gene Ex-pres-sion Om-nibus (GEO) and
 []


Underfull \hbox (badness 4060) in paragraph at lines 1027--1032
```

```
\OT1/bch/m/n/10 Bio-Con-duc-tor. Bioin-for-mat-ics. 2007;23:1846--7.
 []


Underfull \hbox (badness 1708) in paragraph at lines 1033--1039
\OT1/bch/m/n/10 web plat-form for meta-data an-no-ta-tion of ge-nomic
 []


Underfull \hbox (badness 5077) in paragraph at lines 1040--1045
\OT1/bch/m/n/10 Mod-el-ing, In-te-grat-ing Can-cer Ge-nomic Data and
 []


Underfull \hbox (badness 1199) in paragraph at lines 1046--1052
\OT1/bch/m/n/10 of SARS-CoV-2 and other vi-ral species. Database.
 []


Underfull \hbox (badness 4036) in paragraph at lines 1065--1070
\OT1/bch/m/n/10 NC. GE-Ofetch: A command-line tool for down-
 []


Underfull \hbox (badness 10000) in paragraph at lines 1065--1070
\OT1/bch/m/n/10 load-ing data and stan-dard-ized meta-data from
 []


Underfull \hbox (badness 10000) in paragraph at lines 1065--1070
\OT1/bch/m/n/10 GEO and SRA. Bioin-for-mat-ics. 2023;btad069.
 []


Underfull \hbox (badness 2460) in paragraph at lines 1071--1075
[]\OT1/bch/m/n/10 22. Malkov YA, Yashunin DA. Ef-fi-cient and ro-
 []


Underfull \hbox (badness 7504) in paragraph at lines 1071--1075
\OT1/bch/m/n/10 bust ap-prox-i-mate near-est neigh-bor search us-ing
 []


Underfull \hbox (badness 1014) in paragraph at lines 1071--1075
\OT1/bch/m/n/10 Hi-er-ar-chi-cal Nav-i-ga-ble Small World graphs. 2018.
 []


Underfull \hbox (badness 1571) in paragraph at lines 1076--1082
[]\OT1/bch/m/n/10 23. Guo Z, Tzvetkova B, Bassik JM, Bodziak T,
 []
```

```
Underfull \hbox (badness 2547) in paragraph at lines 1076--1082
\OT1/bch/m/n/10 Wo-j-nar BM, Qiao W, et al. RNASe-qMetaDB: A
 []


Underfull \hbox (badness 5681) in paragraph at lines 1076--1082
\OT1/bch/m/n/10 for-mat-ics (Ox-ford, Eng-land). 2015;31:4038--40.
 []

[6]

LaTeX Warning: File `fig/pdf/supplemental.pdf' not found on input line
1106.


! Package pdftex.def Error: File `fig/pdf/supplemental.pdf' not found:
using dr
aft setting.

See the pdftex.def package documentation for explanation.
Type  H <return>  for immediate help.
 ...

l.1106 \includegraphics{fig/pdf/supplemental.pdf}

Try typing  <return>  to proceed.
If that doesn't work, type  X <return>  to quit.


Package hyperref Warning: The anchor of a bookmark and its parent's must
not
(hyperref)                be the same. Added a new anchor on input line
1121.

[7

] (./manuscript_2024-05-10.aux)
 ***********
LaTeX2e <2023-11-01> patch level 1
L3 programming layer <2024-02-20>
 ***********

LaTeX Font Warning: Some font shapes were not available, defaults
substituted.

Package rerunfilecheck Info: File `manuscript_2024-05-10.out' has not
changed.
(rerunfilecheck)             Checksum:
B1D5D803597B1308EA3BA87D3AA1278B;6211.
 )
Here is how much of TeX's memory you used:
 27197 strings out of 474121
 501018 string characters out of 5747949
 1950190 words of memory out of 5000000
```

```
 48968 multiletter control sequences out of 15000+600000
 585652 words of font info for 85 fonts, out of 8000000 for 9000
 1141 hyphenation exceptions out of 8191
 84i,11n,93p,1102b,540s stack positions out of
10000i,1000n,20000p,200000b,200000s
 <c:/Users/adminuser/.texlive2023/texmf-
var/fonts/pk/ljfour/public/ifsym/ifsy
m10.420pk><c:/texlive/2023/texmf-
dist/fonts/type1/bitstrea/charter/bchb8a.pfb><
c:/texlive/2023/texmf-
dist/fonts/type1/bitstrea/charter/bchbi8a.pfb><c:/texlive
/2023/texmf-
dist/fonts/type1/bitstrea/charter/bchr8a.pfb><c:/texlive/2023/texmf
-dist/fonts/type1/bitstrea/charter/bchri8a.pfb><c:/texlive/2023/texmf-
dist/font
s/type1/public/amsfonts/cm/cmitt10.pfb><c:/texlive/2023/texmf-
dist/fonts/type1/
public/amsfonts/cm/cmmi10.pfb><c:/texlive/2023/texmf-
dist/fonts/type1/public/am
sfonts/cm/cmsy8.pfb><c:/texlive/2023/texmf-
dist/fonts/type1/public/amsfonts/cm/
cmtt10.pfb><c:/texlive/2023/texmf-
dist/fonts/type1/public/amsfonts/cm/cmtt9.pfb
><c:/texlive/2023/texmf-
dist/fonts/type1/urw/helvetic/uhvb8a.pfb><c:/texlive/20
23/texmf-dist/fonts/type1/urw/helvetic/uhvr8a.pfb>
Output written on manuscript_2024-05-10.pdf (7 pages, 166698 bytes).
PDF statistics:
 297 PDF objects out of 1000 (max. 8388607)
 261 compressed objects within 3 object streams
 70 named destinations out of 1000 (max. 500000)
 253 words of extra memory for PDF output out of 10000 (max. 10000000)
```

Click here to access/download;LaTeX - Figure (eps, ps, etc.);fig1.pdf

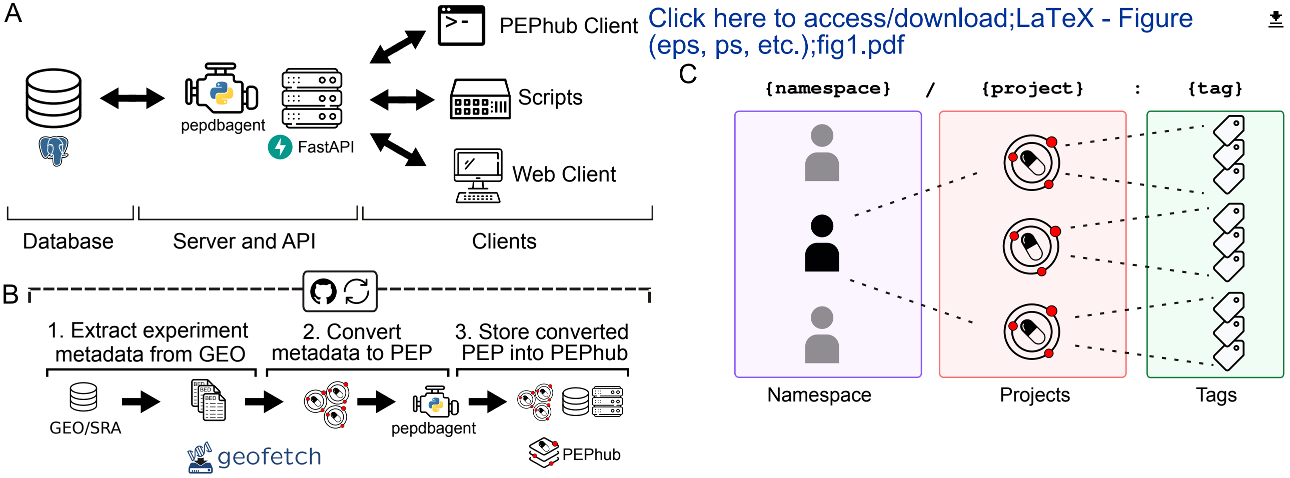

Click here to access/download;LaTeX - Figure (eps, ps, etc.);fig2.pdf

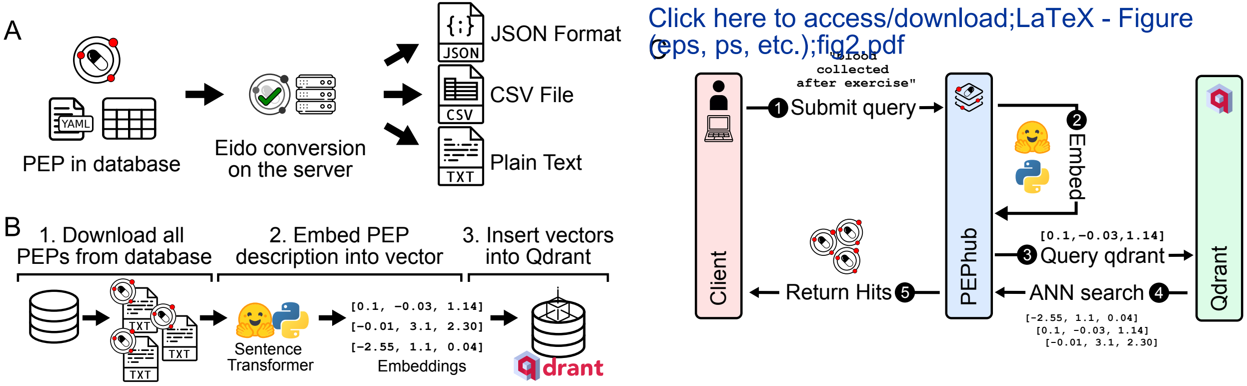

Click here to access/download;LaTeX -

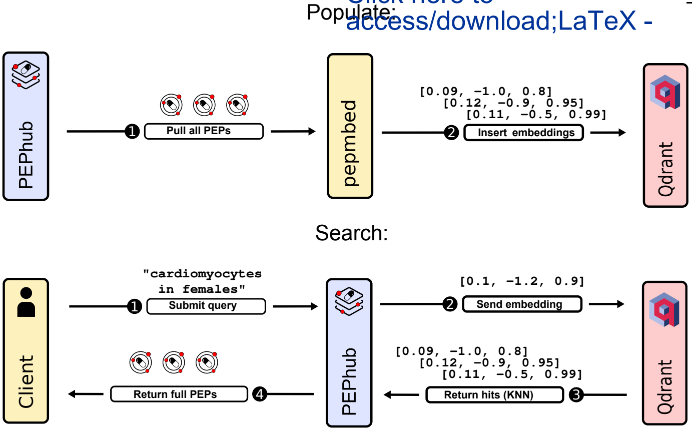

Click here to access/download;LaTeX - Figure (eps, ps, etc.);fig3.pdf

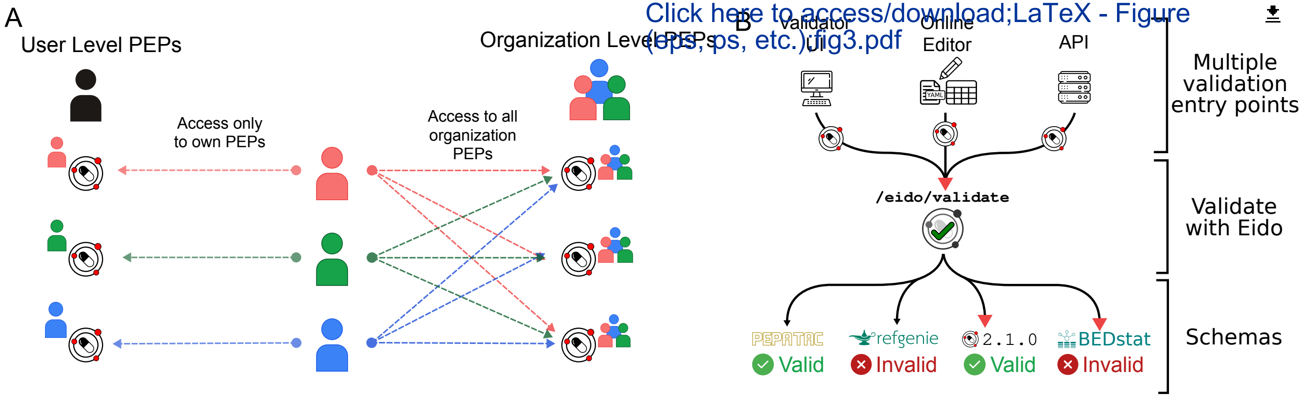

Click here to access/download;LaTeX - Figure (eps, ps, etc.);auth.pdf

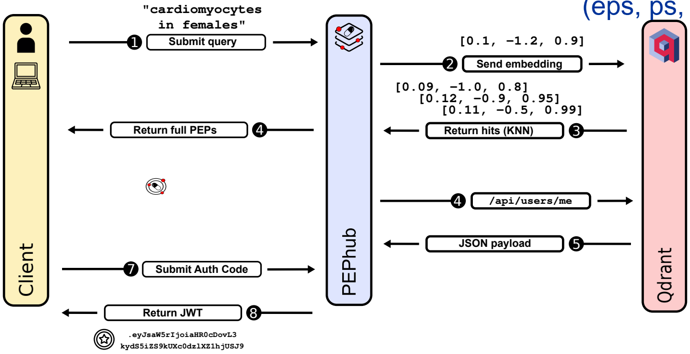

Click here to access/download;LaTe

	Open-Sourced	Self-Hosted	API	Editable Metadata	Command Line Client	ML-Based Search	Has Public Data	Actively Maintained	Public Instance
PEPhub	✓	✓	✓	✓	✓	✓	✓	✓	✓
GenoSurf	✓	✗ [a]	✓	✗	✗	✗	✓	✗	✓
EpiSurf	✓	✗ [a]	✓	✗	✗	✗	✓	✗	✓
OMeta	✓	✗ [a]	✗	✓	✗	✗	✗	✗	✗
GEO	✗	✗	✓	✗	✗	✗	✓	✓	✓
RNASeqMetaDB	✗	✗	✗	✗	✗	✗	✓	✗	✗
LabPipe	✓	✓	✓	✓	✗	✗	✗	✗	✗
METAGENOTE	✓	✗	✗	✓	✗	✗	✗	✗	✓

Click here to access/download;LaTeX - Figure (eps, ps, etc.);metageo_pephub_new.pdf

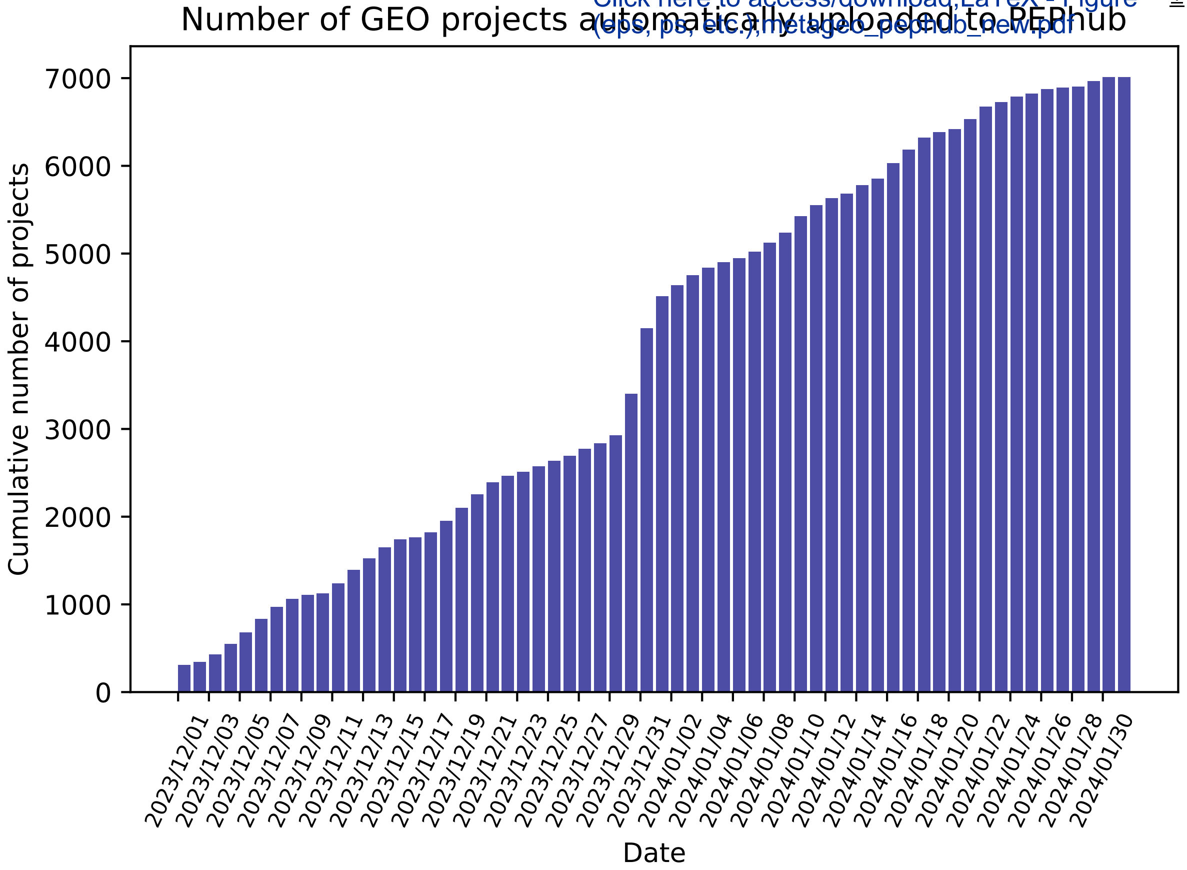

Click here to access/download;LaTeX - Figure (eps, ps, etc.);supplemental.pdf

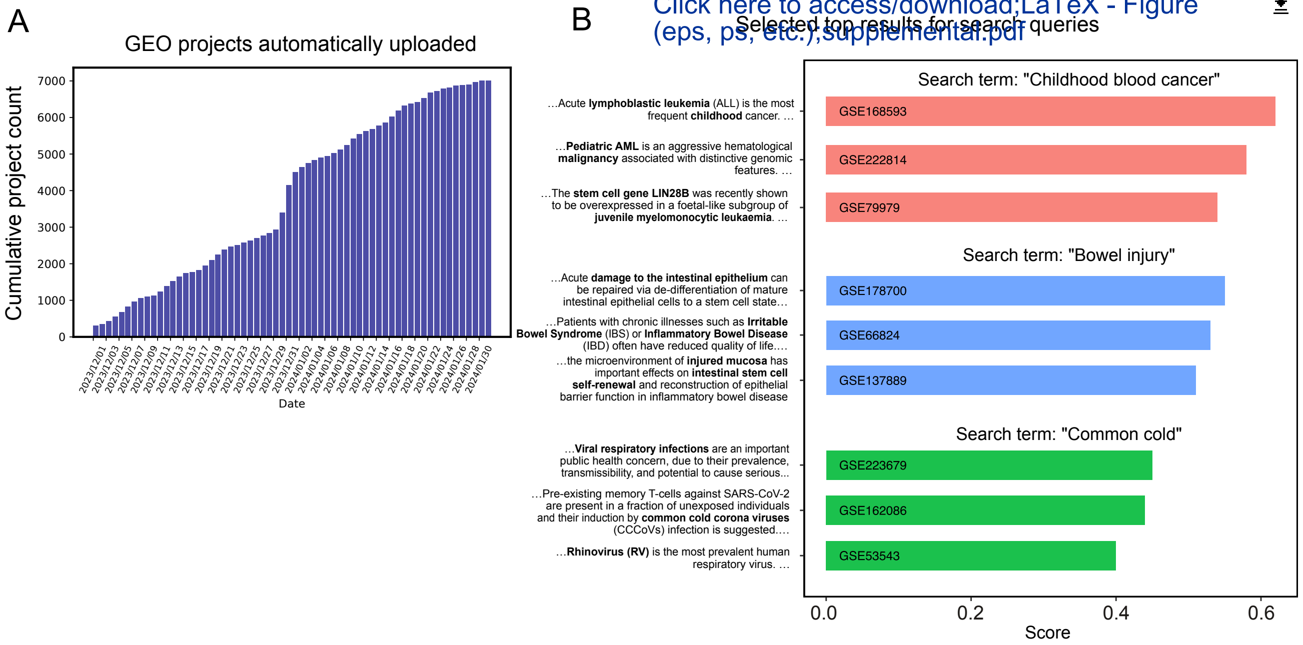

Manuscript

Click here to access/download;Manuscript;manuscript-2024-05-10 (2) (2).pdf

PEPhub: a database, web interface, and API for editing, sharing, and validating biological sample metadata

Nathan J. LeRoy[1, 3], Oleksandr Khoroshevskyi[1], Aaron O'Brien[1], Rafał Stepień[1], Alip Arslan[7], and Nathan C. Sheffield[1-6,✉]

[1]Center for Public Health Genomics, School of Medicine, University of Virginia, 22908, Charlottesville VA
[2]School of Data Science, University of Virginia, Charlottesville VA 22904, Charlottesville VA
[3]Department of Biomedical Engineering, School of Medicine, University of Virginia, 22904, Charlottesville VA
[4]Department of Public Health Sciences, School of Medicine, University of Virginia, 22908, Charlottesville VA
[5]Department of Biochemistry and Molecular Genetics, School of Medicine, University of Virginia, 22908, Charlottesville VA
[6]Child Health Research Center, School of Medicine, University of Virginia, 22908, Charlottesville VA
[7]Department of Computer Science, School of Engineering, University of Virginia, 22908, Charlottesville VA
✉ Correspondence: nsheffield@virginia.edu

Background: As biological data increases, we need additional infrastructure to share it and promote interoperability. While major effort has been put into sharing data, relatively less emphasis is placed on sharing metadata. Yet, sharing metadata is also important, and in some ways has a wider scope than sharing data itself. **Results:** Here, we present PEPhub, an approach to improve sharing and interoperability of biological metadata. PEPhub provides an API, natural language search, and user-friendly web-based sharing and editing of sample metadata tables. We used PEPhub to process more than 100,000 published biological research projects and index them with fast semantic natural language search. PEPhub thus provides a fast and user-friendly way to finding existing biological research data, or to share new data. **Availability:** https://pephub.databio.org

Background

The rapid pace of biological data generation has led to challenges with data sharing, storage, and integration [1–4]. Growing interest in data reusability and interoperability [5, 6] has led to new effort in improving biological data sharing and accessibility [7–9]. However, most effort has focused on biological *data*. Less emphasis has been placed on increasing the availability of biological *metadata* [10, 11].

As such, it is helpful to distinguish between *data* and *metadata*. In biology, *data* consists of experimental measurements or observations, while *metadata* describes the biological sample from which the measurements were derived. The sample metadata may include inherent, experimental, or analytical attributes about the sample. It might also describe the biology, treatments, experimental conditions, and data analysis parameters. Sharing complete biological *metadata* is important not only for integrated analysis, but also for discoverability [6]. There is a critical need for better tools and frameworks for sharing biological metadata.

To this end, tools and repositories have been developed to work with biological metadata [12–16]. However, they suffer from four main limitations: First, while metadata databases exist, they tend to focus on storage and retrieval [12]; none focus on simplifying user upload and editing of their own data. Second, metadata generally lacks a well-defined and supported structure. Previous methods tend to use a structure for a specific tool and data source [16–18], or leave the structure undefined altogether. Third, their search functionality is limited. Metadata search is generally limited to string matching or ontology searches. Finally, existing metadata services cannot easily be rebuilt and redeployed efficiently for custom use [19].

A recent advancement in biological metadata interoperability is Portable Encapsulated Projects (PEP), a framework that provides a standardized metadata structure, metadata validation, and programmatic metadata modifiers [20]. A PEP is a standardized sample table. The PEP framework provides a common infrastructure that links sample tables to analytical tools by removing the need for tedious and manual data preparation, mitigating the problem of metadata interoperability. However, there is no user-friendly web interface and API for sharing sample tables in the PEP ecosystem.

Here, we address these limitations with PEPhub: a database, web interface, search engine, and API for sharing, retrieving, and validating biological sample metadata. PEPhub provides several features that improve biological metadata interoperability, including: user- and machine-oriented interfaces, user editing and sharing, format conversion, metadata validation, natural language search, and containers for custom deployment.

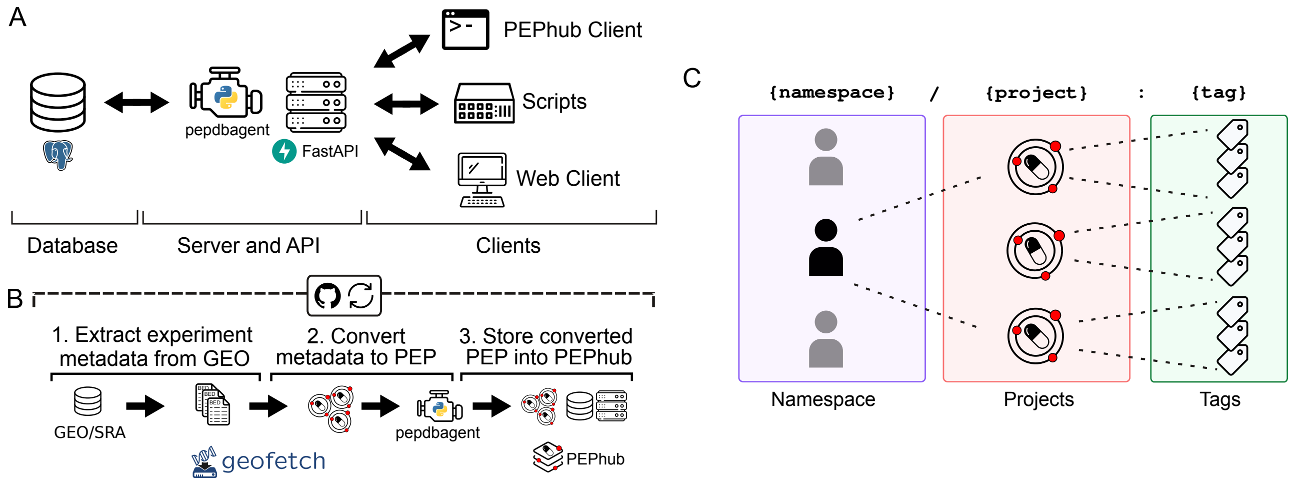

Figure 1. PEPhub high-level architecture and project identification strategy. A. *PEPhub is backed by a Postgres database (left). It interfaces with the PEPhub server through a companion package called `pepdbagent` (middle). Web requests made by the web client or command-line interface are made via HTTP (right).* ***B.*** *Workflow for automated GEO-to-PEPhub transfer using GEOfetch. We take advantage of scheduled Github Actions to automate new discovery of GEO accessions to upload.* ***C.*** *PEPhub employs a {namespace}/{project}:{tag} nomenclature for sample table identification. Namespaces contain projects, which can be further distinguished with tags.*

PEPhub advances the accessibility, discoverability, and reusability of biological sample metadata.

Results and Discussion

PEPhub instance and user interface

Public PEPhub instance

PEPhub is a web service for biological sample metadata. It is implemented as three major components that work together as modules: 1) a FastAPI web service; 2) a PostgreSQL database; 3) the PEPhubClient Python package, which provides Python and command-line interfaces to PEPhub (Figure 1A; Methods). To showcase the PEPhub software, we deployed a publicly available instance (see Availability). We used GEOfetch [21] to populate this public instance with over 150,000 projects (PEPs) derived from the Gene Expression Omnibus (GEO), with automated updates (Figure 1B, S1A; see Methods). PEPhub organizes projects by namespaces, corresponding to a user or organization on GitHub, thereby grouping related projects. PEPs are identified using a registry path in the form of <namespace>/<project_name>:<tag> (Figure 1C). The project name identifies a sample table. This naming convention allows convenient reference and versioning of sample metadata tables.

User- and machine-oriented interfaces

There are two primary interfaces by which users may interact with a PEPhub instance. First, the web interface provides access to PEP metadata for human browsing. It encourages data exploration and collaboration, making it easier for researchers to browse, search, submit, and edit PEPs. Second, the programmatic API allows other programs and scripts to interact with the server through HTTP requests. The API emphasizes the modularity of the PEPhub architecture and promotes interoperability with external software and services.

Format conversion

PEPhub provides programmatic interfaces to convert metadata into multiple formats. The standard PEP structure includes project-level attributes, a sample table, and a subsample table, which allows users to encode sample attributes with multiple values, such as sequencing reads with multiple file paths. By default, PEPhub offers the ability to convert this metadata into JSON, YAML, CSV, and plain-text formats (Figure 2A). To achieve this, PEPhub takes advantage of `eido`, a metadata validation engine written in Python [20]. Metadata conversion increases the interoperability of metadata, allowing it to fit into any analysis pipeline. Further, `eido` lets you write your own conversion functions, expanding the capabilities of a custom PEPhub deployment.

Natural language search

To improve biological metadata discovery, PEPhub provides a powerful natural-language search engine. The search engine is powered by pre-trained sentence transformers and a Qdrant vector database (Methods). We first use a sentence transformer to create low-dimensional vector representations of each PEP from the project-level and sample-level metadata attributes and descriptions. We store the resulting vectors inside a Qdrant vector database instance (Figure 2B). When a user provides a natural language search query, PEPhub transforms the query using the same sentence transformer in real-time, then queries the Qdrant API to retrieve the most semantically similar PEP vectors. Qdrant identifies similar PEPs by calculating nearest neighbors in vector space. PEPhub then returns the results to the client with their associated description and registry path (Figure 2C). PEPhub's search engine uses a *semantic* approach, which provides several advantages:

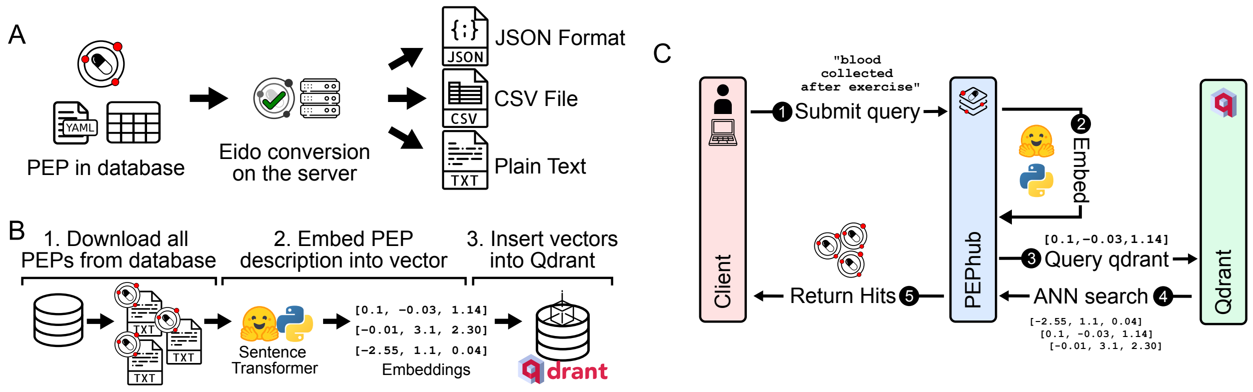

*Figure 2. **Metadata sharing, discovery, and accessibility features.** **A.** PEPhub can convert metadata into `JSON`, `csv`, and `txt` output. **B.** Using a pre-trained sentence transformer, we periodically compute low-dimensional embeddings of all PEPs in PEPhub by mining text descriptions from the metadata. The resulting embeddings are then stored in Qdrant: a vector similarity engine and vector database. These embeddings are then compared against user-submitted queries. **C.** Searching for a PEP in pephub using vector search happens in five steps. First, the user submits a natural language query. Second, this query is embedded in real-time on the server. Third, the resultant vector is used to query Qdrant for nearest neighbors. Fourth, Qdrant responds with the most similar vectors it has stored. Finally, the hits are returned to the client submitting the query.*

First, the system returns results with similar meaning whether or not they include the terms of the original query. Second, it is tolerant of misspellings and is not limited to any ontology or taxonomy. Finally, because each PEP is represented as a vector, we can use high-speed nearest-neighbor algorithms to identify relevant PEPs, making the search very fast [22]. This method scales to millions of PEPs, and the speed is limited only by network speeds. Users may also tune results with limits, offsets, and relevance score cutoffs (Methods). To demonstrate the value of PEPhub's semantic search, we show how some possible search terms like "childhood blood cancer" are able to retrieve more specialized related datasets (Figure S1B, Supplementary Table 1).

Private and collaborative metadata upload and editing

While the natural language search and API access to standard structured metadata from GEO is valuable, one of the most important features of PEPhub is the ability for users to submit and edit their own PEPs. Users can submit and then edit their own PEPs on PEPhub through the API or through the web interface. To facilitate this, PEPhub also provides a robust authentication system. Users authenticate with PEPhub using GitHub, which provides user and organization namespaces. Users have read access to all namespaces but write access only to their namespaces. For PEPs with write access, users may mark them as *private* to restrict read access to only users with write access (Figure 3A). For example, **user1234** can edit all PEPs in the **user1234/** namespace. They may also edit all PEPs in the **org1234** namespace if they are a public member on GitHub. This ensures that only authorized users can access and modify private PEPs. By integrating authentication and authorization features, PEPhub provides a secure and controlled environment for users to interact with and manage their own PEPs while also facilitating the sharing and discovery of public PEPs to support collaborative research efforts.

Metadata validation

PEPhub also provides metadata validation. We use `eido`, a PEP validation tool based on `jsonschema`, to validate on the server [20]. There are three ways to validate metadata through PEPhub. First, you may use the web-validator UI built with the server. With the web-validator UI, you may upload your own PEP or use PEPs stored on PEPhub and validate them against either PEPhub schemas or custom schemas. Custom schemas can be uploaded or pasted directly on the UI. Second, you can take advantage of the built-in metadata builder. When editing your PEPs, PEPhub validates the PEP after each save. The interface will propagate any errors to the user. Finally, there are validation endpoints at `/eido/validate`. These allow programmatic validation of PEPs (Figure 3B).

Comparison to other tools

Currently, several biological metadata management solutions exist to help alleviate the issue of metadata accessibility and interoperability; however, these solutions suffer from one or more limitations. One example is OMeta [19]. While it shares some features with PEPhub, it is not actively maintained, it lacks a public instance, and it lacks any documentation to start a private instance. Another example is RNASeqMetaDB [23]. Like PEPhub, RNASeqMetaDB aims to solve the problem of disorganized and limited access to sample metadata that are often published alongside the data itself; however, it lacks a currently running public instance and the source code is not available to reproduce the results. Finally, EpiSurf [18] and GenoSurf [12] are comprehensive genomics metadata search servers. However, they are limited in three ways: first, they don't permit users to submit their own metadata and the database doesn't appear to be regularly updated. Second, the software is not easily deployable. Third, their search system is based

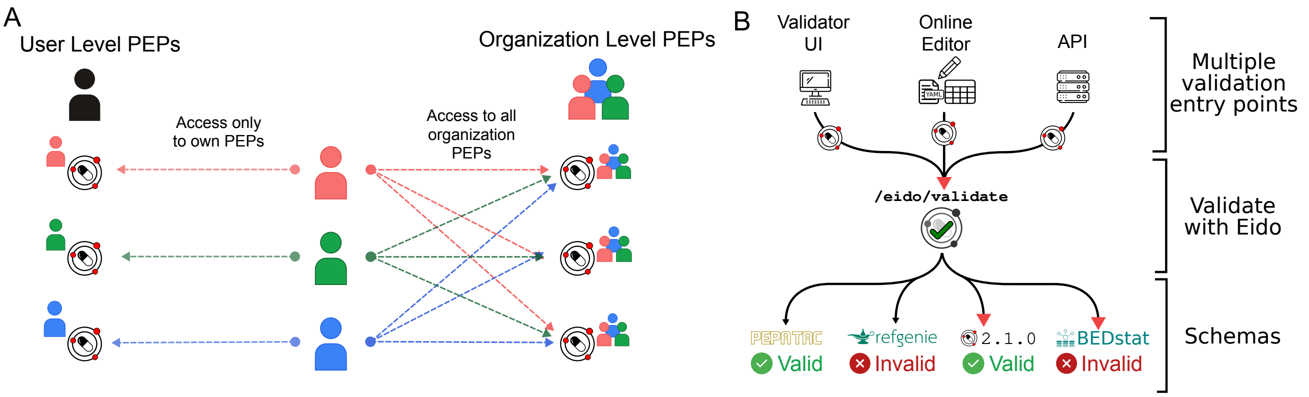

Figure 3. Metadata privacy and validation features. ***A.*** *Users have read access to all namespaces but write access only to their namespaces (left). Other users are not permitted to modify a PEP in any user namespace other than their own. PEPhub implements organizations through GitHub. Members of an organization are automatically granted write access to all PEPs that belong to that organization (right).* ***B.*** *Validation on PEPhub is made easy with the integration of* `eido`*. PEPs in PEPub can be validated using either the web-based validator UI, the metadata builder, or programmatic endpoints.*

	Open-Sourced	Self-Hosted	API	Editable Metadata	Command Line Client	ML-Based Search	Has Public Data	Actively Maintained	Public Instance
PEPhub	✓	✓	✓	✓	✓	✓	✓	✓	✓
GenoSurf	✓	✗ [a]	✓	✗	✗	✗	✓	✗	✓
EpiSurf	✓	✗ [a]	✓	✗	✗	✗	✓	✗	✓
OMeta	✓	✗ [a]	✗	✓	✗	✗	✗	✗	✗
GEO	✗	✗	✓	✗	✗	✗	✓	✓	✓
RNASeqMetaDB	✗	✗	✗	✗	✗	✗	✓	✗	✗
LabPipe	✓	✓	✓	✓	✗	✗	✗	✗	✗
METAGENOTE	✓	✗	✗	✓	✗	✗	✗	✗	✓

Figure 4. Metadata management comparison chart. *PEPhub compares favorably to alternative metadata management systems. *a: While open-source, no clear documentation exists for self hosting an instance.*

solely on biological ontologies, limiting the search space and flexibility of the search system.

PEPhub has several features that make it unique: First, it prioritizes allowing users to edit sample metadata stored on the server. This critical design decision positions PEPhub as not just as a place to *find* metadata, but as a place to manage and share your own. Second, it provides a full database, web API, and user interface for metadata management. Third, it provides the only metadata search engine that takes advantage of pre-trained sentence transformers for a powerful semantic search system. Fourth, it is the only tool that is open-sourced, automatically updated, actively maintained, and provides clear instructions to deploy a private instance. Together, these features make PEPhub a unique, flexible, tool that promotes the accessibility, findability, and interoperability of biological sample metadata.

PEPhub may also be compared to a Laboratory Information Management System (LIMS), a broad term with several interpretations. One difference is that LIMS tend to target management challenges related to physical sample handling in a wet lab, such as connecting to machinery, ordering reagents, and tracking physical samples through an experimental protocol. PEPhub could fulfill some of these functions, but in general, PEPhub targets a later phase of the experimental process: after data is generated, and the samples need to be analyzed and shared. PEPhub's strengths are the ability to share data easily, in a more universal form, a public API, standardization, and searching. Thus, in many cases, it may make sense for PEPhub to live alongside a LIMS.

Future development of PEPhub

We have several plans for PEPhub development. First, with some basic adapters, PEPhub could simplify the process of submitting data to public repositories, such as SRA or GEO. We are interested in working with interested parties to explore how to simplify the data submission process. Second, we plan to extend PEPhub to serve as a source for data analysis. We already have adopted our pipeline engine, looper, to retrieve sample tables from PEPhub. We plan to extend this functionality to other pipeline engines, such as Snakemake. Finally, PEPhub has potential to serve as a pipeline management dashboard, wherein processes could send updates to the server as pipelines run. We are currently developing software called `pipestat`, which provides a standardized mechanism for pipelines to report results, and we are exploring ways to link PEPhub with running pipelines such that they can be monitored directly from the web interface.

Methods: Implementation and deployment details

FastAPI web service

The PEPhub server is built with FastAPI, a web framework optimized for speed and high-performance. FastAPI is specifically designed for developing APIs. We chose FastAPI for its automatic data validation capabilities, built-in API documentation, and because using Python allows us to interface with existing Python infrastructure for metadata management we developed previously [20]. The FastAPI application uses our companion package `pepdbagent` to interface with a Postgres database. The user interface is built using React.js and TypeScript, and is packaged with the server.

`pepdbagent` companion package

To manage project creation, fetching, deletion and insertion into the database, we developed a companion package called `pepdbagent`. `pepdbagent` acts as a simple wrapper around the popular Python object-relational mapper (ORM) `SQLAlchemy` to provide a convenient API for managing projects in our database. Both PEPhub itself and all maintenance scripts use `pepdbagent` to manage the PEPs stored inside the database.

PostgreSQL database

Postgres is well-suited for storing the structured and unstructured data found in PEPs because it excels at both relational and document storage. The PEPhub database is comprised of three tables. The first table, `projects` stores the PEPs metadata and PEPs configuration. The `projects` table consists of twelve columns to store data like the project timestamp, project id, and the project configuration as a JSON blob. The other two tables: `samples`, and `subsamples` store samples and subsamples that are linked with `project.id` to a specific project in the `project` table. We host the public PEPhub database instance on the Amazon Web Services Relational Database Service (AWS RDS).

PEPhubClient Python and command-line interface

To facilitate command-line interaction and third-party tools using PEPhub, we have developed PEPhubClient. PEPhubClient is a command-line interface and Python API that leverages the machine-oriented interfaces of PEPhub. Namely, the public API. PEPhubClient makes it easy to push and pull PEPs to any PEPhub instance. The command-line interface supports authentication to make authorized requests. This includes working with private PEPs, downloading PEPs, editing PEPs, and submitting PEPs. The PEPhubClient CLI is implemented in Python using the `typer` library.

Containers for custom deployment

To standardize deployment and promote interoperability, we've packaged the PEPhub server and database as docker containers. These containers are made available on dockerhub. This makes it easy to launch your own instance of PEPhub.

Populating PEPhub with biological sample metadata from GEO

To populate PEPhub, we developed a pipeline to ingest sample metadata from GEO. Our pipeline uploads PEPs from GEO in two steps. First, it identifies experiments that were added or updated in certain period of time to the Gene Expression Omnibus[7] using the GEOfetch Python API [21]. Second, GEOfetch downloads, formats and produces PEPs from GEO experiments that are later uploaded to the PEPhub database. Our database now stores more than 150,000 high-throughput sample tables from the last 10 years from Gene Expression Omnibus. We developed a pipeline that uses GitHub `schedule` actions to automate the download, formatting, and upload or re-upload of new project releases on GEO. Moreover, the pipeline includes an automatic check for successful previous uploads, ensuring that all GEO projects are consistently updated on PEPhub without the need for manual intervention.

Natural language search

To support the text mining and embedding pipeline, we developed a companion tool called `pepembed` that embeds a database of PEPs and inserts them into a Qdrant database instance. For each PEP in the database `pepembed` does three things: first, it *flattens* the `yaml` representation of the sample metdata to create a continuous string; second, it utilizes an embedding model (e.g. sentence transformer) to produce a low-dimensional vector representation of this text; finally, `pepembed` will insert this vector in a Qdrant instance along with that PEPs namespace, name, and tag. We leverage GitHub actions to run indexing tasks periodically to ensure that all PEPs stay properly indexed, even if their data change. `pepembed` is open-source and available on GitHub.

Authentication and authorization

PEPhub supports two authentication flows: authorization code flow and device code flow. Both take advantage of GitHub's OAuth services. In addition, both authentication flows require users to login with GitHub via a web browser, upon which a code is returned. This code is then exchanged for a JSON Web Token (JWT) via a `POST` request which can be used to make subsequently authorized requests. While very similar, both flows exist to make it as easy as possible to integrate third-party software with a PEPhub instance.

Funding

This work was supported by the National Institute of General Medical Sciences grant R35-GM128636 (NCS) and National Human Genome Research Institute grant R01-HG012558 (NCS). Funders had no role in study design, data collection, analysis, or publication.

Availability of supporting source code and requirements

Project name: PEPhub
Project home page: https://pephub.databio.org
Operating system: Platform independent
Programming language: Python
License: BSD-2
bio.tools ID: pephub
SciCrunch RRID: SCR_024892

Data Availability

All supporting data and materials are available in the GigaScience GigaDB database [24].

Conflict of interest statement

NCS is a consultant for InVitro Cell Research, LLC.

References

1. Volchenboum SL, Cox SM, Heath A, Resnick A, Cohn SL, Grossman R. Data Commons to Support Pediatric Cancer Research. American Society of Clinical Oncology Educational Book. 2017;746–52. doi:10.1200/EDBK_175029.

2. Bui AAT, Van Horn JD. Envisioning the future of 'big data' biomedicine. Journal of Biomedical Informatics. 2017;69:115–7. doi:10.1016/j.jbi.2017.03.017.

3. Armit C, Tuli MA, Hunter CI. A Decade of GigaScience: GigaDB and the Open Data Movement. GigaScience. 2022;11:giac053. doi:10.1093/gigascience/giac053.

4. Xue B, Khoroshevskyi O, Gomez RA, Sheffield NC. Opportunities and challenges in sharing and reusing genomic interval data. Frontiers in Genetics. 2023;14. doi:10.3389/fgene.2023.1155809.

5. Wilkinson MD, Dumontier M, Aalbersberg IjJ, Appleton G, Axton M, Baak A, et al. The FAIR Guiding Principles for scientific data management and stewardship. Scientific Data. 2016;3:160018. doi:10.1038/sdata.2016.18.

6. Sheffield NC, Bonazzi VR, Bourne PE, Burdett T, Clark T, Grossman RL, et al. From biomedical cloud platforms to microservices: Next steps in FAIR data and analysis. Scientific Data. 2022;9:553. doi:10.1038/s41597-022-01619-5.

7. Edgar R, Domrachev M, Lash AE. Gene Expression Omnibus: NCBI gene expression and hybridization array data repository. Nucleic Acids Research. 2002;30:207–10.

8. Sloan CA, Chan ET, Davidson JM, Malladi VS, Strattan JS, Hitz BC, et al. ENCODE data at the ENCODE portal. Nucleic Acids Research. 2016;44:D726–32. doi:10.1093/nar/gkv1160.

9. Bourne PE, Bonazzi V, Dunn M, Green ED, Guyer M, Komatsoulis G, et al. The NIH Big Data to Knowledge (BD2K) initiative. Journal of the American Medical Informatics Association : JAMIA. 2015;22:1114. doi:10.1093/jamia/ocv136.

10. Leipzig J, Nüst D, Hoyt CT, Ram K, Greenberg J. The role of metadata in reproducible computational research. Patterns. 2021;2:100322. doi:10.1016/j.patter.2021.100322.

11. Sheffield N, LeRoy N, Khoroshevskyi O. Challenges to sharing sample metadata in computational genomics. Frontiers in Genetics. 2023;14.

12. Canakoglu A, Bernasconi A, Colombo A, Masseroli M, Ceri S. GenoSurf: Metadata driven semantic search system for integrated genomic datasets. Database. 2019;2019:baz132. doi:10.1093/database/baz132.

13. Serna Garcia G, Leone M, Bernasconi A, Carman MJ. GeMI: Interactive interface for transformer-based Genomic Metadata Integration. Database. 2022;2022:baac036. doi:10.1093/database/baac036.

14. Masseroli M, Pinoli P, Venco F, Kaitoua A, Jalili V, Palluzzi F, et al. GenoMetric Query Language: A novel approach to large-scale genomic data management. Bioinformatics. 2015;31:1881–8. doi:10.1093/bioinformatics/btv048.

15. Davis S, Meltzer PS. GEOquery: A bridge between the Gene Expression Omnibus (GEO) and BioConductor. Bioinformatics. 2007;23:1846–7. doi:10.1093/bioinformatics/btm254.

16. Quiñones M, Liou DT, Shyu C, Kim W, Vujkovic-Cvijin I, Belkaid Y, et al. "METAGENOTE: A simplified web platform for metadata annotation of genomic samples and streamlined submission to NCBI's sequence read archive." BMC Bioinformatics. 2020;21:378. doi:10.1186/s12859-020-03694-0.

17. Cappelli E, Cumbo F, Bernasconi A, Canakoglu A, Ceri S, Masseroli M, et al. OpenGDC: Unifying, Modeling, Integrating Cancer Genomic Data and Clinical Metadata. Applied Sciences. 2020;10:6367. doi:10.3390/app10186367.

18. Bernasconi A, Cilibrasi L, Al Khalaf R, Alfonsi T, Ceri S, Pinoli P, et al. EpiSurf: Metadata-driven search server for analyzing amino acid changes within epitopes of SARS-CoV-2 and other viral species. Database. 2021;2021:baab059. doi:10.1093/database/baab059.

19. Singh I, Kuscuoglu M, Harkins DM, Sutton G, Fouts DE, Nelson KE. OMeta: An ontology-based, data-driven metadata tracking system. BMC bioinformatics. 2019;20:8. doi:10.1186/s12859-018-2580-9.

20. Sheffield NC, Stolarczyk M, Reuter VP, Rendeiro AF. Linking big biomedical datasets to modular analysis with

portable encapsulated projects. GigaScience. 2021;10. doi:10.1093/gigascience/giab077.

21. Khoroshevskyi O, LeRoy N, Reuter VP, Sheffield NC. GEOfetch: A command-line tool for downloading data and standardized metadata from GEO and SRA. Bioinformatics. 2023;btad069. doi:10.1093/bioinformatics/btad069.

22. Malkov YA, Yashunin DA. Efficient and robust approximate nearest neighbor search using Hierarchical Navigable Small World graphs. 2018. doi:10.48550/arXiv.1603.09320.

23. Guo Z, Tzvetkova B, Bassik JM, Bodziak T, Wojnar BM, Qiao W, et al. RNASeqMetaDB: A database and web server for navigating metadata of publicly available mouse RNA-Seq datasets. Bioinformatics (Oxford, England). 2015;31:4038–40. doi:10.1093/bioinformatics/btv503.

24. LeRoy NJ, Khoroshevskyi O, O'Brien A, Stepień R, Arslan A, Sheffield NC. Supporting data for "PEPhub: A database, web interface, and API for editing, sharing, and validating biological sample metadata". GigaScience Database. 2024. doi:10.5524/102510.

Supplemental figures

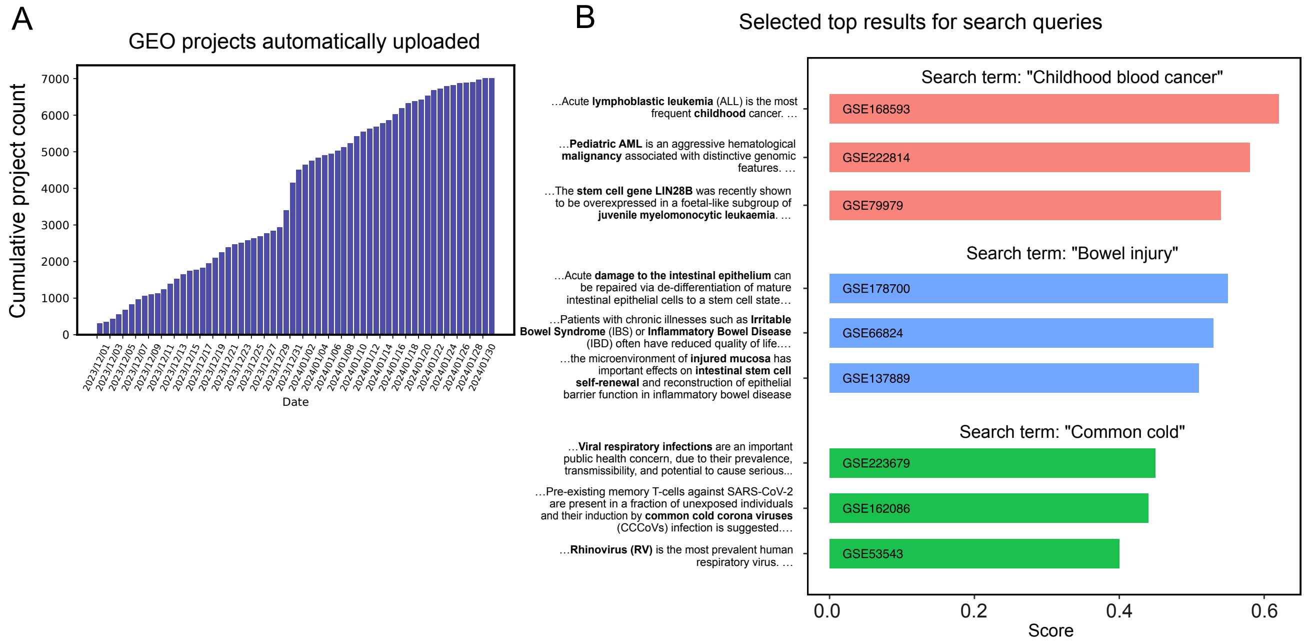

Supplementary Figure S1. Analysis of PEP metadata and search. *A) Barchart showing the cumulative number of new biological sample tables added to PEPhub from GEO, automatically. PEPhub automatically indexed more than 1000 new projects during this 3-week demo span. B) Illustrative search result scores selected from the top 10 responses returned by the PEPhub semantic search engine for biological search terms shown*

Supplemental text

Search result examples

Illustrative search results from PEPhub. Search results from GEO and PEPhub for common queries. The PEPhub search engine returns results that are more relevant to the query than the GEO search engine. Moreover, the results returned by PEPhub are more diverse.

Search term: "Childhood blood cancer"

GEO results:

- . . . effects of MZ1 on multiple molecular subtypes of B-cell acute lymphoblastic leukemia cells (GSE217540)
- . . . PAF1 and FACT to drive high density enhancer interactions in leukemia (GSE202451)
- . . . PAF1 and FACT to drive high density enhancer interactions in leukemia. . . (GSE202450)
- . . . PAF1 and FACT to drive high density enhancer interactions in leukemia. . . (GSE202449)

PEPhub results:

- . . . genomic alterations in radiation-related breast cancer among childhood cancer survivors. . . (GSE62940)
- . . . analysis of CD10+/CD19+ pre B lymphoblasts from bone marrow and peripheral blood of B-ALL patients (GSE168593)
- . . . DNA methylation profiling predicts relapse in childhood B-cell acute lymphoblastic leukemia (GSE39141)
- . . . analysis of pediatric histiocytic sarcomas and anteceding hematologic malignancies (GSE109904)

Search term: "Bowel injury"

GEO results:

- tumors from mice fed diets excluding methionine/tryptophan/niacin (GSE246627)
- tumors from mice fed diets excluding methionine/tryptophan/niacin (GSE246626)
- Airway Microfold (M) Cells Emerge in the Post-IAV Lung (GSE244279)

PEPhub results:

- Colonic mucosal injury responses (GSE164918)

Search term: “Common cold”

GEO results:

- ATAC-Seq of Batf-deficient pDC Transcriptomes... (GSE178410)
- Deterministic reprogramming of neutrophils in tumors (GSE244536)
- ...Pancreatic Tumors Reveal Distinct Compartmentalisation of Neutrophil Subsets (GSE244534)
- ...of neutrophil subsets in a mouse model of pancreatic cancer (GSE244531)

PEPhub results:

- A longitudinal study of natural respiratory viral infections (GSE223679)
- ...temperature variation controls pre-mRNA processing and transcription of anti-viral genes (GSE193639)
- Influenzavirus serotype association to global whole blood transcriptional changes (GSE29385)
- The immune response and microbiota profiles during co-infection with P. vivax... (GSE144792)
