# Supplementary figures and images for: PEPhub: a database, web interface, and API for editing, sharing, and validating biological sample metadata
Source: Gigascience. 2024 Jul 11;13:giae033. doi: 10.1093/gigascience/giae033 (PMC11238423; doi:10.1093/gigascience/giae033)

A

GEO projects automatically uploaded

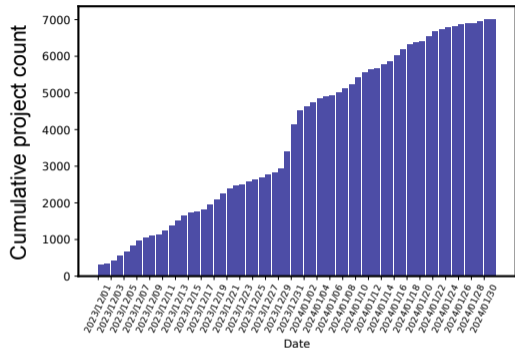

B

Selected top results for search queries

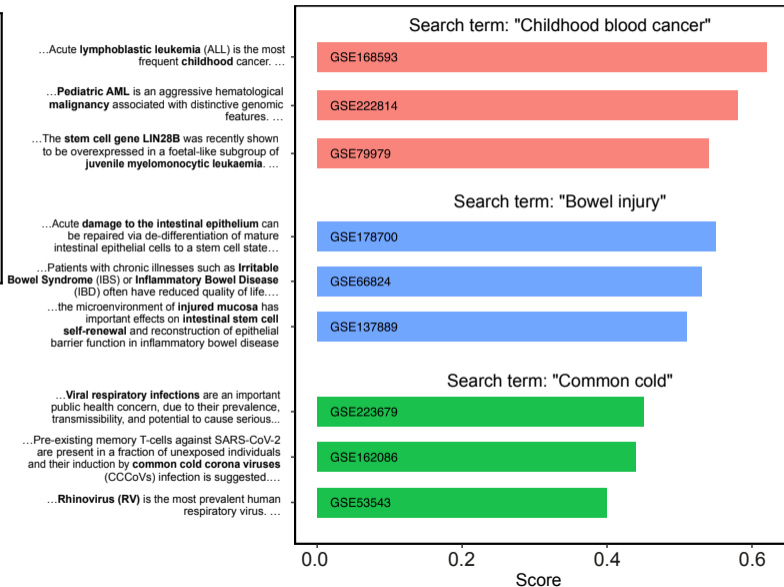

Supplement: giae033_Supplemental_File [file giae033_supplemental_file.pdf]
