# Supplementary material for: PEPhub: a database, web interface, and API for editing, sharing, and validating biological sample metadata
Source: Gigascience. 2024 Jul 11;13:giae033. doi: 10.1093/gigascience/giae033 (PMC11238423; doi:10.1093/gigascience/giae033)
Supplement: giae033_Response_to_Reviewer_Comments_Revision_2 [file giae033_response_to_reviewer_comments_revision_2.pdf]

Author's Response To Reviewer Comments

ok
