# Supplementary material for: PEPhub: a database, web interface, and API for editing, sharing, and validating biological sample metadata
Source: Gigascience. 2024 Jul 11;13:giae033. doi: 10.1093/gigascience/giae033 (PMC11238423; doi:10.1093/gigascience/giae033)
Supplement: giae033_Reviewer_1_Report_Original_Submission — Jeremy Leipzig -- 9/4/2023 Reviewed [file giae033_reviewer_1_report_original_submission.pdf]

Reviewer Report

Title: PEPhub: a database, web interface, and API for editing, sharing, and validating biological sample metadata

Version: Original Submission **Date:** 9/4/2023

Reviewer name: Jeremy Leipzig

Reviewer Comments to Author:

Metadata describes the who, what, where, when, and why of an experiment. Sample metadata is arguably the most important of these, but not the only type. LeRoy et al describes a user-centric sample metadata management system with extensibility, support for multiple interface modalities, and fuzzy semantic search.This system and portal, PEPHub, bridges the gaps between LIMS, which are tightly bound to the wet lab, metadata fetchers like GEOfetch (from the same group) or pysradb, and public portals like MetaSRA and the others listed in . Then and both of which don't allow you to roll your own portal internally, and whose search criteria are not fuzzy or semantic.People have been storing metadata in bespoke databases for decades, but not in an interoperable mature fashion. The PepHUB portal builds on some existing Pep standards by the same group, introducing a restful API and GUI.I find this paper a novel and compelling submission but would like the following minor revisions:1. Typically in SRA a sample refers to a dna sample drawn from a tissue sample (ie BioSample) and then runs describe sequencing attempts on those dna samples, and files are produced from each of the runs. It is unclear to me how someone working in an internal lab using PEPHub would know how to extract the file locations of sequence files associated with a sample if these are many-to-one. In the GEO example provided I can click on the SRX link to see the runs and files but how would this work for an internally generated entry? I need the authors to explain this either as a response or in the text.2. I think the paper has to briefly describe how the authors envision how PEPhub should interface with or replaces a LIMS for labs that are producing their own data and describe how it can help accelerate the SRA submission process for these data generating labs.3. Change "Bernasconi2021" to META-BASE in the text4. Some of the search confidence measures show an absurd level of significant digits (e.g.56.99999999999999% Please round that as it's only used for sorting.

Level of Interest

Please indicate how interesting you found the manuscript: Choose an item.

Quality of Written English

Please indicate the quality of language in the manuscript: Choose an item.

Declaration of Competing Interests

Please complete a declaration of competing interests, considering the following questions:

- Have you in the past five years received reimbursements, fees, funding, or salary from an organisation that may in any way gain or lose financially from the publication of this manuscript, either now or in the future?
- Do you hold any stocks or shares in an organisation that may in any way gain or lose financially from the publication of this manuscript, either now or in the future?
- Do you hold or are you currently applying for any patents relating to the content of the manuscript?
- Have you received reimbursements, fees, funding, or salary from an organization that holds or has applied for patents relating to the content of the manuscript?
- Do you have any other financial competing interests?
- Do you have any non-financial competing interests in relation to this paper?

If you can answer no to all of the above, write 'I declare that I have no competing interests' below. If your reply is yes to any, please give details below.

I declare that I have no competing interests

I agree to the open peer review policy of the journal. I understand that my name will be included on my report to the authors and, if the manuscript is accepted for publication, my named report including any attachments I upload will be posted on the website along with the authors' responses. I agree for my report to be made available under an Open Access Creative Commons CC-BY license (http://creativecommons.org/licenses/by/4.0/). I understand that any comments which I do not wish to be included in my named report can be included as confidential comments to the editors, which will not be published.

Choose an item.

To further support our reviewers, we have joined with Publons, where you can gain additional credit to further highlight your hard work (see: https://publons.com/journal/530/gigascience). On publication of this paper, your review will be automatically added to Publons, you can then choose whether or not to claim your Publons credit. I understand this statement.

Yes Choose an item.
