# Supplementary material for: PEPhub: a database, web interface, and API for editing, sharing, and validating biological sample metadata
Source: Gigascience. 2024 Jul 11;13:giae033. doi: 10.1093/gigascience/giae033 (PMC11238423; doi:10.1093/gigascience/giae033)
Supplement: giae033_Reviewer_2_Report_Original_Submission — Weiwen Wang -- 10/11/2023 Reviewed [file giae033_reviewer_2_report_original_submission.pdf]

Reviewer Report

Title: PEPhub: a database, web interface, and API for editing, sharing, and validating biological sample metadata

Version: Original Submission **Date:** 10/11/2023

Reviewer name: Weiwen Wang

Reviewer Comments to Author:

This manuscript by LeRoy et al. introduces PEPhub, a database aimed at enhancing the sharing and interoperability of biological metadata using the PEP framework. One of the key highlights of this manuscript is the visualization of the PEP framework, which improves the adoption of the PEP framework, facilitating the reuse of metadata. Additionally, PEPhub integrates data from GEO, making it convenient for users to access and utilize. Furthermore, PEPhub offers metadata validation, allowing users to quickly compare their PEP with other PEPhub schemas. Another notable feature is the natural language search, which further enhances the user experience. Overall, PEPhub provides a comprehensive solution that promotes efficient metadata sharing, while leveraging the impact of the PEP framework in organizing large-scale biological research projects.While this manuscript was interesting to read, I have several concerns regarding its "semantic" search system and the interaction of PEPHub.1. The authors mentioned their use of a tool called "pepembed" to embed PEP descriptions into vectors. However, I was unable to locate the tool on GitHub, and there is limited information in the Method section regarding this. Could the authors provide additional details regarding the process of embedding vectors?2. The authors implemented semantic search as an advantage of PEPhub. However, they did not evaluate the effectiveness of their natural language search engine, such as assessing accuracy, recall rate, or F1 score. It would be beneficial for the authors to perform an evaluation of their natural language search engine and provide metrics to demonstrate its performance. This would enhance the credibility and reliability of their claims regarding the advantages of natural language search in PEPhub.3. It would be more beneficial to include the metadata in the search system rather than solely relying on the project description. For instance, when I searched for SRX17165287 (https://pephub.databio.org/geo/gse211736?tag=default), no results were returned.4. When creating a new PEP, it appears that I can submit two samples with identical values. According to the PEP framework guidelines, it is mentioned that "Typically, samples should have unique values in the sample table index column". Therefore, the authors should enhance their metadata validation system to enforce this uniqueness constraint.Additionally, if I enter two identical values in the sample field and then attempt to add a SUBSAMPLE, an error occurs. However, when I modify one of the samples, I am able to save it successfully.5. The error messages should provide more specific guidance. Currently, when attempting to save metadata with an incorrect format, all error messages are displayed as: "Unknown error occurred: Unknown".6. PEPhub should consider providing user guidelines or examples on how to fill in subsample metadata and any relevant rules associated with it.7. In the Validation module, what are the rules for validation? Does it only check for the required column names in the schema, or does it also validate the content of the metadata, such as whether the metadata is in the

correct format (e.g., int or string)? Additionally, it would be beneficial to provide an option to download the relevant schema and clearly specify the required column names in the schema. This would enable users to better organize their PEP to comply with the schema format and ensure that their metadata is accurately validated.8. This version of PEPHub primarily focuses on metadata. Have the authors considered any plans to expand this database to include data/pipeline management within the PEP framework? It would be valuable for the authors to discuss their future plans for PEPHub in this manuscript.Some minor concerns:1. When searching for content within a specific namespace, it would be beneficial for the pagination bar at the bottom of the webpage to display the number of pages. Now there are only Previous/Next buttons.2. As a web service, it is better to show the supporting browsers, such as Google Chrome (version xxx and above), Firefox (version xxx and above). I failed to open PEPHub website using an old version of Chrome.

Level of Interest

Please indicate how interesting you found the manuscript: Choose an item.

Quality of Written English

Please indicate the quality of language in the manuscript: Choose an item.

Declaration of Competing Interests

Please complete a declaration of competing interests, considering the following questions:

- Have you in the past five years received reimbursements, fees, funding, or salary from an organisation that may in any way gain or lose financially from the publication of this manuscript, either now or in the future?
- Do you hold any stocks or shares in an organisation that may in any way gain or lose financially from the publication of this manuscript, either now or in the future?
- Do you hold or are you currently applying for any patents relating to the content of the manuscript?
- Have you received reimbursements, fees, funding, or salary from an organization that holds or has applied for patents relating to the content of the manuscript?
- Do you have any other financial competing interests?
- Do you have any non-financial competing interests in relation to this paper?

If you can answer no to all of the above, write 'I declare that I have no competing interests' below. If your reply is yes to any, please give details below.

I declare that I have no competing interests.

I agree to the open peer review policy of the journal. I understand that my name will be included on my report to the authors and, if the manuscript is accepted for publication, my named report including any attachments I upload will be posted on the website along with the authors' responses. I agree for my report to be made available under an Open Access Creative Commons CC-BY license (http://creativecommons.org/licenses/by/4.0/). I understand that any comments which I do not wish to be included in my named report can be included as confidential comments to the editors, which will not be published.

Choose an item.

To further support our reviewers, we have joined with Publons, where you can gain additional credit to further highlight your hard work (see: https://publons.com/journal/530/gigascience). On publication of this paper, your review will be automatically added to Publons, you can then choose whether or not to claim your Publons credit. I understand this statement.

Yes Choose an item.
